# Comparative Proteomic Profiling: Cellular Metabolisms Are Mainly Affected in *Senecavirus* A-Inoculated Cells at an Early Stage of Infection

**DOI:** 10.3390/v13061036

**Published:** 2021-05-31

**Authors:** Fuxiao Liu, Bo Ni, Rong Wei

**Affiliations:** 1 College of Veterinary Medicine, Qingdao Agricultural University, Qingdao 266109, China; laudawn@126.com; 2 Surveillance Laboratory of Livestock Diseases, China Animal Health and Epidemiology Center, Qingdao 266032, China; nibo@cahec.cn

**Keywords:** *Senecavirus* A, proteomics, differentially expressed protein, enrichment analysis, metabolism, pathway

## Abstract

*Senecavirus* A (SVA), also known as Seneca Valley virus, belongs to the genus *Senecavirus* in the family *Picornaviridae*. SVA can cause vesicular disease and epidemic transient neonatal losses in pigs. This virus efficiently propagates in some non-pig-derived cells, like the baby hamster kidney (BHK) cell line and its derivate (BSR-T7/5). Conventionally, a few proteins or only one protein is selected for exploiting a given mechanism concerning cellular regulation after SVA infection in vitro. Proteomics plays a vital role in the analysis of protein profiling, protein-protein interactions, and protein-directed metabolisms, among others. Tandem mass tag-labeled liquid chromatography-tandem mass spectrometry combined with the parallel reaction monitoring technique is increasingly used for proteomic research. In this study, this combined method was used to uncover separately proteomic profiles of SVA- and non-infected BSR-T7/5 cells. Furthermore, both proteomic profiles were compared with each other. The proteomic profiling showed that a total of 361 differentially expressed proteins were identified, out of which, 305 and 56 were upregulated and downregulated in SVA-infected cells at 12 h post-inoculation, respectively. GO (Gene Ontology) and KEGG (Kyoto Encyclopedia of Genes and Genomes) enrichment analyses showed that cellular metabolisms were affected mainly in SVA-inoculated cells at an early stage of infection. Therefore, an integrated metabolic atlas remains to be explored via metabolomic methods.

## 1. Introduction

*Senecavirus* A (SVA), previously known as Seneca Valley virus, is an emerging pathogen in China [1,2]. SVA infection is characterized by porcine vesicular lesions on coronary bands as well as snout and oral cavities, clinically indistinguishable from those caused by foot-and-mouth disease virus, swine vesicular disease virus and vesicular stomatitis virus [3,4]. SVA is classified into the genus *Senecavirus* in the family *Picornaviridae*. Its genome is a positive-sense, single-stranded and nonsegmented RNA, approximately 7300 nucleotides in length, with a 3′ poly (A) tail but without a 5′ capped structure. The genome contains 5′ and 3′ untranslated regions and a single long open reading frame of polyprotein precursor [5]. Morphologically, mature virion is a non-enveloped icosahedral particle with a diameter of approximately 27 nm. Purified virion reveals a sphere-like shape as evidenced by transmission electron microscopy [6]. Some pig-derived cells, such as testis (CRL-1746) and kidney (PK-15) cell lines, are susceptible to SVA infection. SVA can also propagate efficiently in some non-pig-derived cells, like the baby hamster kidney (BHK) cell line and its derivate (BSR-T7/5). Additionally, SVA is an oncolytic virus, with selective tropism for some tumors with neuroendocrine characteristics [7,8,9].

SVA infection triggers a variety of metabolic and biochemical changes in its host cells through virus-specific or -nonspecific mechanisms [10,11,12,13]. For example, SVA has been demonstrated to have the ability to induce autophagy or (and) apoptosis of host cells. The SVA-induced autophagy was evidenced by detecting autophagosome formation, GFP-LC3 puncta and accumulation of LC3-II proteins in cultured PK-15 and BHK-21 cells [10]. The SVA-induced apoptosis resulted from the SVA 3C protease, by which the NF-κB-p65 was cleaved at the late stage of infection [13]. Additionally, the innate immune system plays an important role in SVA infection. Wen et al. (2019) demonstrated that SVA replication could induce the degradation of RIG-I in HEK-293T, SW620 and SK6 cells. Overexpression of RIG-I significantly inhibited SVA propagation. The viral 2C and 3C proteins notably reduced Sev- or RIG-I-induced interferon-β production [12].

According to conventional methods, a few host and (or) virus proteins are generally selected for exploiting a given mechanism of cellular regulation against viral infection. Integrated omics can assist in deciphering complex networks in vitro or in vivo and especially in unveiling the interaction among related molecules to affect disease outcome [14]. Proteomic research plays a crucial role in analyzing protein-protein interactions, profiles and kinetics of protein expression, as well as complex regulatory mechanisms. Owing to its high-efficiency, -sensitivity and -throughput characteristics, the tandem mass tag (TMT)-labeled method has been broadly used for quantitative analysis of proteomics [15,16,17]. Parallel reaction monitoring (PRM) is a novel technique, is better than conventional methods to verify proteins, and provides reliable results of quantitative proteomics [18,19,20]. Additionally, with the development of bioinformatics, the efficiency of large-scale data processing has been greatly improved in the field of proteomic studies. The strategy, TMT-based quantitative analysis with PRM verification, has been gradually adopted for proteomic profiling [21,22,23].

We recently rescued a wild-type SVA (CH-LX-01-2016) from its cDNA clone using reverse genetics. The rescued virus could rapidly replicate in BSR-T7/5 cells and induced typical cytopathic effect (CPE) as early as 24 hpi, or even earlier [24]. In the present study, in order to uncover a proteomic profile of SVA-infected cells for further comparison to that of non-infected cells, the rescued SVA at passage 5 (P5) was used to inoculate BSR-T7/5 cells, subsequently subjected to a series of treatments for the proteomic analysis using TMT-labeled nano-liquid chromatography-tandem mass spectrometry (LC-MS/MS) followed by PRM verification. Comparative proteomic analysis showed that cellular metabolisms were affected mainly at the early stage of SVA infection.

## 2. Materials and Methods

### 2.1. Cell Line and Virus

The BSR-T7/5 cells [25], derived from the BHK cell line, were cultured at 37 °C with 5% CO_2_ in Dulbecco’s modified Eagle’s medium (DMEM), supplemented with 4% fetal bovine serum and containing penicillin (100 U/mL), streptomycin (100 µg/mL), amphotericin B (0.25 µg/mL) and G418 (500 µg/mL). The wild-type SVA was rescued previously from a cDNA clone [24], genetically derived from an isolate, CH-LX-01-2016 (Genbank access No.: KX751945) [26].

### 2.2. Sample Preparation

BSR-T7/5 cells were seeded into several T175 flasks for culture at 37 °C. Cell monolayers at 90% confluency were separately inoculated with the P5 SVA (MOI = 2.5), and the others served as non-infected controls. There were three SVA-infected samples (S1, S2 and S3) and three non-infected controls (C1, C2 and C3). At 12 h post-inoculation (hpi), SVA- and non-infected supernatants were removed from flasks. Cell monolayers were gently washed with D-PBS three times and then independently harvested into sterile 50 mL centrifuge tubes using cell scrapers. After centrifugation, cell pellets were suspended in 300 μL of SDT-lysis buffer (4% SDS, 100 mM DTT, 150 mM Tris-HCl pH 8.0), followed by boiling for 5 min, ultrasonic treatment for 2 min, and boiling for another 5 min. Undissolved cellular debris was removed by centrifugation at 15,000× *g* for 20 min. The supernatants were collected and quantified with the BCA Protein Assay Kit (Bio-Rad, Richmond, VA, USA), followed by SDS-PAGE analysis.

### 2.3. Protein Digestion

Digestion of protein (300 μg for each sample) was performed according to the procedure of filter-aided sample preparation (FASP), as described previously [27]. Briefly, 300 µg of protein was added to DTT at a final concentration of 100 mM, boiled for 5 min, and then cooled to room temperature. The DTT and other low-molecular-weight components were removed using 200 μL of UA buffer (8 M urea, 150 mM Tris-HCl pH 8.0) by repeated ultrafiltration (Microcon units, 10 kD), facilitated by centrifugation at 12,000× *g* for 15 min. A total of 100 μL of IAA (50 mM iodoacetamide in UA buffer) was added to the concentrate, shaken at 600 rpm for 1 min, and incubated for 30 min in darkness, followed by centrifugation at 12,000× *g* for 10 min. A total of 100 μL of UA buffer was added to the concentrate, followed by centrifugation three times at 12,000× *g* for 10 min; then, 100 μL of TEAB buffer was added to the concentrate, followed by centrifugation three times at 14,000× *g* for 10 min. Forty μL of trypsin buffer (6 µg trypsin in 40 µL TEAB buffer) was added to the concentrate, subsequently shaken at 600 rpm for 1 min, and incubated at 37 °C for 16 h. The digests were collected by centrifugation at 12,000× *g* for 10 min using another Microcon device. The collected peptides were resolved in 0.1% TFA solution, and desalted on C18 Cartridges.

### 2.4. TMT Labeling of Peptides

Peptides were labeled with TMT reagents (Thermo Fisher, Waltham, MA, USA), as described previously [28]. Briefly, the collected peptides were lyophilized, and then reconstituted further in 100 μL of 200 mM TEAB solution. For labeling, each TMT reagent was dissolved in 41 μL of anhydrous acetonitrile using a vortex agitator for 5 min, followed by centrifugation. Then, 41 μL of the TMT reagent was mixed with 40 μL of the peptide solution and incubated at room temperature for 1 h. The reaction was terminated by addition of 8 μL of 5% hydroxylamine, followed by incubation for 15 min. Finally, the labeled peptide samples were lyophilized and stored at −80 °C.

### 2.5. High-pH Reversed-Phase Fractionation

TMT-labeled peptide mixture was fractionated as described by Gu et al. (2020), with slight modifications [29]. A Waters XBridge BEH130 column (C18, 3.5 μm, 2.1 × 150 mm) was used on an Agilent 1290 HPLC operating at 0.3 mL/min. The buffer A of mobile phase consisted of 10 mM ammonium formate with water, and the buffer B consisted of 10 mM ammonium formate with 90% (*v*/*v*) acetonitrile. Both buffers were adjusted to pH 10 with ammonium hydroxide. A total of fifteen fractions were collected using a 2 min interval with 30 min gradient and were further combined into ten fractions by a concatenation strategy. The fractions were lyophilized and resuspended in 0.1% FA for nano-LC-MS/MS analysis.

### 2.6. Nano-LC-MS/MS Analysis

The nano-LC-MS/MS analysis was performed on a Q-Exactive HF-X mass spectrometer that was coupled to Easy nLC 1200 system (Thermo Fisher, Waltham, MA, USA). Solution A was 0.1% formic acid aqueous solution; solution B was a mixed solution of 0.1% formic acid, 80% acetonitrile and water. The column was first equilibrated with 100% solution A. The sample was injected into a Trap column (100 µm × 20 mm, 5 µm C18, Dr. Maisch GmbH, Ammerbuch-Entringen, Germany) and subjected to gradient separation through a chromatography column (75 µm × 150 mm, 3 µm C18, Dr. Maisch GmbH, Ammerbuch-Entringen, Germany) at a flow rate of 300 nL/min. The linear gradient was set as follows: 0–3 min, linear gradient from 2 to 7% buffer B; 3–48 min, linear gradient from 7 to 35% buffer B; 48–53 min, linear gradient from 35 to 90% buffer B; 53–60 min, buffer B maintained at 90%.

The peptides were separated and subjected to a data-dependent acquisition (DDA) analysis for 60 min using the Q-Exactive HF-X mass spectrometer. The parameters were set as follows: detection mode: positive mode; parent ion scanning range: 300–1800 *m*/*z*; first-order MS resolution: 60,000 at *m*/*z* 200; AGC target: 3e6; first-level maximum IT: 50 ms. Peptide secondary MS was obtained as follows: for each full scan, MS data was acquired using a top-20 method for dynamically choosing the most intense parent ion from second-order MS (MS2) scan. The parameters were set as follows: MS2 resolution: 15,000 at *m*/*z* 200; AGC target: 1e5; level-2 maximum IT: 50 ms; MS2 activation type: HCD; isolation window: 1.6 *m*/*z*; normalized collision energy: 32 eV.

### 2.7. Integrated Analysis of Proteomic Data

Proteome Discoverer^TM^ Software (version 2.4, San Jose, CA, USA) [30] was used to search MS/MS spectra against the database UniProt Cricetulus griseus 56565-20201207.fasta using the SEQUEST HT search engine (downloaded on 7 December 2020 and including 56565 protein sequences). The search parameters used were as follows: Type: Reporter ion MS2; Isobaric labels: TMT 6plex; Enzyme: Trypsin; Reporter mass tolerance: 0.005 Da; Max Missed Cleavages: 2; Peptide Tolerance: 10 ppm; MS/MS Tolerance: 0.02 Da; Fixed modifications: Carbamidomethyl (C); Variable modifications: Oxidation (M), Acetyl (Protein N-term), Deamidation (N, Q), TMT (K) and TMT (N-term); Database: UniProt Cricetulus griseus 56565-20201207.fasta; Database pattern: Target-Reverse; Percolator (FDR): ≤0.01; Protein quantification: Razor and unique peptides were used for protein quantification.

### 2.8. Quantitative Analysis by LC-PRM/MS

To verify the expression levels of different proteins obtained by the TMT-labeled proteomic analysis, a total of twenty proteins were selected for further quantitative analysis by LC-PRM/MS, as described by Li et al. (2019), with slight modifications [23]. Briefly, 2 µg of peptide from each sample was taken for LC-PRM/MS analysis. After sample loading, chromatographic separation was performed using the EASY-nLC nano-HPLC system (Thermo Fisher, Waltham, MA, USA), with two buffers. Solution A was 0.1% formic acid aqueous solution, and solution B was a mixture of 0.1% formic acid, 95% acetonitrile and water. The column was first equilibrated with 95% solution A. The sample was injected into a Trap column (100 µm × 20 mm, 5 µm C18, Dr. Maisch GmbH, Ammerbuch-Entringen, Germany) and then subjected to gradient separation through a chromatography column (75 µm × 150 mm, 3 µm C18, Dr. Maisch GmbH, Ammerbuch-Entringen, Germany) at a flow rate of 300 nL/min. The liquid phase separation gradient was as follows: 0–5 min, linear gradient of B liquid from 2 to 5%; 5–45 min, linear gradient of B liquid from 5 to 23%; 45–50 min, linear gradient of B liquid from 23 to 40%; 50–52 min, linear gradient of B liquid from 40 to 100%; 52–60 min, B liquid maintained at 100%.

The peptides were separated and then subjected to targeted PRM/MS using the Q-Exactive HF-X mass spectrometer (Thermo Fisher, Waltham, MA, USA) for 60 min. The parameters were set as follows: detection mode: positive mode; parent ion scanning range: 300–1200 *m*/*z*; first-order MS resolution: 60,000 at *m*/*z* 200; AGC target: 3e6; first-level maximum IT: 50 ms. Peptide secondary MS was obtained as follows: for each full scan, target peptides of precursor m/z were sequentially selected based on the inclusion list for MS2 scan. The parameters were set as follows: MS2 resolution: 17,500 at *m*/*z* 200; AGC target: 1e6; level-2 maximum IT: 100 ms; MS2 activation type: HCD; isolation window: 1.6 Th; normalized collision energy: 27 eV. The raw data were analyzed using the Skyline 4.1 [31] to obtain the signal intensities of individual peptide sequences.

## 3. Results

### 3.1. Proteomic Profiles of SVA- and Non-Infected Cells

**Figure 1:**
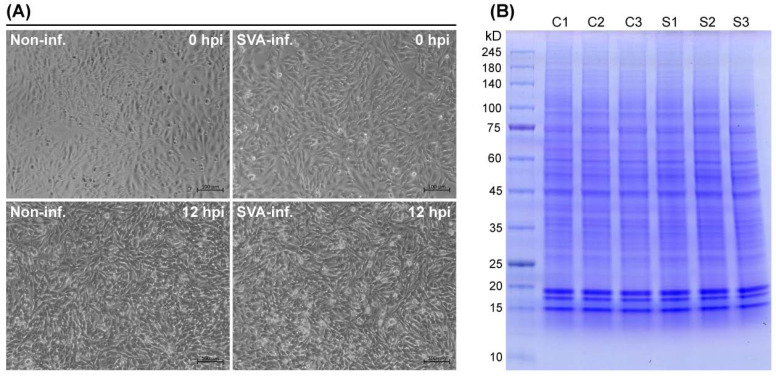
Sample preparation for SDS-PAGE analysis. SVA- and non-infected BSR-T7/5 cells at 0 and 12 hpi (**A**). Cell samples are collected from six groups for further lysis, followed by SDS-PAGE analysis (**B**).

SVA induced no typical CPE on BSR-T7/5 cells at 12 hpi (Figure 1A). Cells were independently collected from the six groups (S1, S2, S3, C1, C2 and C3) and then subjected to a series of treatments for quantitative analysis, showing that protein concentrations of group S1, S2, S3, C1, C2 and C3 were 22.72, 23.92, 17.56, 15.33, 12.15 and 13.55 μg/μL, respectively, as measured by the BCA Protein Assay Kit. There was no observable difference among these six groups through the SDS-PAGE analysis (Figure 1B). To explore differences of protein expressions between SVA- and non-infected cells, quantitative proteomic analysis was carried out to characterize the protein samples from the six groups using the TMT-labeled nano-LC-MS/MS.

**Figure 2:**
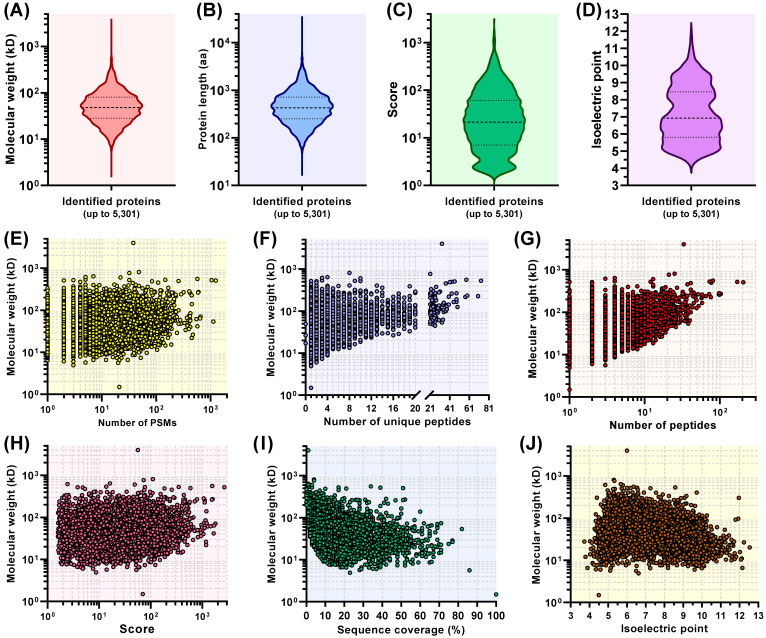
Comprehensive proteomic profiling of six groups. Violin plot-exhibited distributions of molecular weight (**A**), protein length (**B**), score (**C**) and isoelectric point (**D**) for all 5301 identified proteins. Corresponding relations between the molecular weight and the number of PSMs (**E**), the number of unique peptides (**F**), the number of peptides (**G**), score (**H**), sequence coverage (**I**) and isoelectric point (**J**) for each identified protein.

A total of 5301 proteins were identified and quantified across the six groups (Supplementary file S1). Protein-related data were processed and comprehensively evaluated for obtaining proteomic profiles. Figure 2A–D represented actual distributions of molecular weight, protein length, score and isoelectric point for all identified proteins, respectively. The titin (UniProt accession No.: G3HAC6) and histone H1 (UniProt accession No.: Q9QUY8) were determined to have the maximum (4004.6 kD) and minimum (1.5 kD) molecular weights, respectively. Figure 2E–J revealed corresponding relations between the molecular weight and the number of peptide-spectrum matches (PSMs), the number of unique peptides, the number of peptides, score, sequence coverage as well as isoelectric point for each identified protein, respectively.

### 3.2. Analysis of Differentially Expressed Proteins (DEPs)

**Figure 3:**
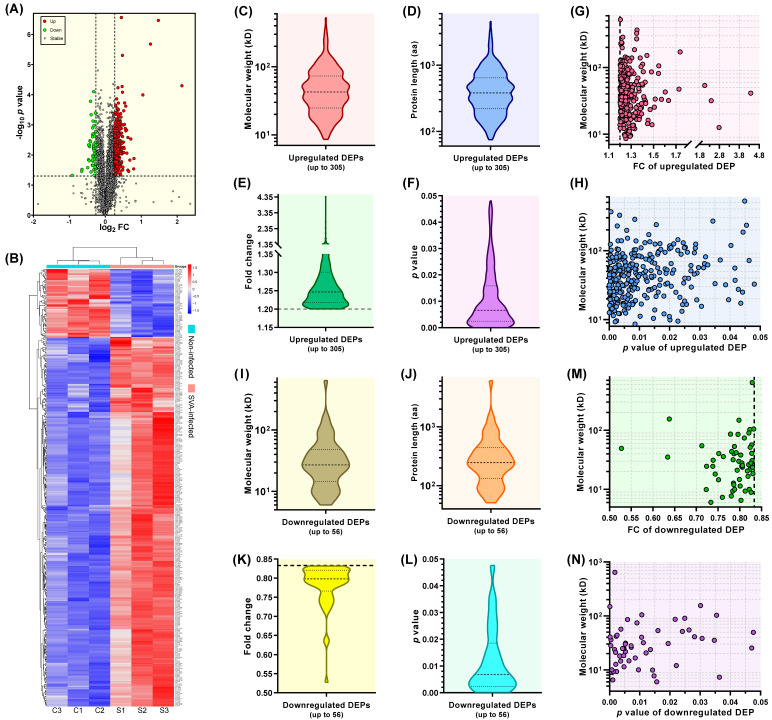
Profiles of upregulated and downregulated DEPs. Volcano plot of all 5301 proteins identified in the six groups (**A**). Up: upregulated DEPs (FC > 1.2 and *p* value < 0.05); Down: downregulated DEPs (FC < 0.833 and *p* value < 0.05); Stable: proteins neither significantly upregulated nor significantly downregulated (0.833 ≤ FC ≤ 1.2 or *p* value ≥ 0.05). Heatmap with hierarchical clustering of 361 DEPs (FC > 1.2 or < 0.833 and *p* value < 0.05) (**B**). The intensity of color reflects the degree of change. Red: highly expressed DEPs; blue: lowly expressed DEPs. Violin-plot-exhibited distributions of molecular weight (**C**), protein length (**D**), FC (**E**) and *p* value (**F**) for all 305 upregulated DEPs. Corresponding relations between the molecular weight and FC (**G**) and *p* value (**H**) for each upregulated DEP. Violin-plot-exhibited distributions of molecular weight (**I**), protein length (**J**), FC (**K**) and *p* value (**L**) for all 56 downregulated DEPs. Corresponding relations between the molecular weight and FC (**M**) as well as *p* value (**N**) for each downregulated DEP.

The volcano plot was generated by the GraphPad Prism software (Version 8.0) to display the *p* value versus the fold change (FC) for all 5301 identified proteins (Figure 3A). Using a 1.2-fold increase or decrease in protein expression as a benchmark for a significant change, a total of 361 DEPs (*p* < 0.05) were identified, out of which, 305 and 56 were upregulated (Supplementary file S2) and downregulated (Supplementary file S3) in SVA-infected cells at 12 hpi, respectively. The heatmap showed hierarchical clustering of 361 DEPs (FC > 1.2 or < 0.833 and *p* value < 0.05) (Figure 3B). Figure 3C–F represented actual distributions of molecular weight, protein length, FC and *p* value for all upregulated proteins, respectively. Figure 3G,H revealed corresponding relations between the molecular weight and the FC as well as *p* value for each upregulated protein, respectively. Figure 3I–L represented actual distributions of molecular weight, protein length, FC and *p* value for all downregulated proteins, respectively. Figure 3M,N revealed corresponding relations between the molecular weight and the FC as well as *p* value for each downregulated protein, respectively.

### 3.3. GO (Gene Ontogy) Enrichment Analysis

**Figure 4:**
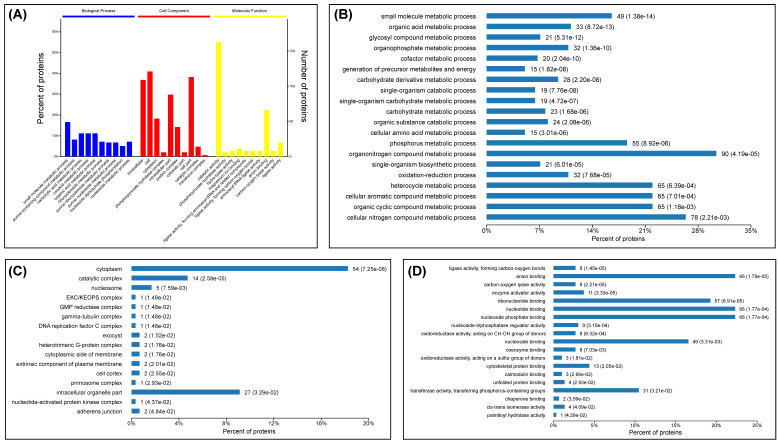
GO enrichment analysis of statistically significant DEPs in SVA-infected cells at 12 hpi. Top 10 statistically significant GO terms of three categories (biological process, cell component and molecular function) (**A**). Major GO terms that are significantly enriched in biological processes (**B**), cell component (**C**), and molecular function (**D**). Each bar is followed by the number of DEPs with the corresponding *p* value.

DEPs were subjected to analysis in the GO database. According to the GO enrichment analysis, 248, 147 and 258 DEPs were assigned to the categories “biological process (BP)” (Supplementary file S4), “cell component (CC)” (Supplementary file S5) and “molecular function (MF)” (Supplementary file S6), respectively. Diverse biological changes were identified in multiple GO terms at 12 hpi: the numbers of statistically significant (*p* < 0.05) BP, CC and MF enrichments were 262, 55 and 146, respectively (Supplementary files S4–S6). The top 10 statistically significant GO terms were separately selected from these three categories and are shown in Figure 4A. Major significant enrichments of the BP, CC and MF are shown in Figure 4B–D, respectively. The most significant enrichments of BP, CC and MF concerned the small molecule metabolic process (GO: 0044281, *p* value: 1.38E-14, including 49 DEPs), the intracellular (GO: 0005622, *p* value: 7.04E-12, including 109 DEPs) and the catalytic activity (GO: 0003824, *p* value: 2.73E-11, including 163 DEPs), respectively (Supplementary S4–S6).

### 3.4. KEGG (Kyoto Encyclopedia of Genes and Genomes) Enrichment Analysis

**Figure 5:**
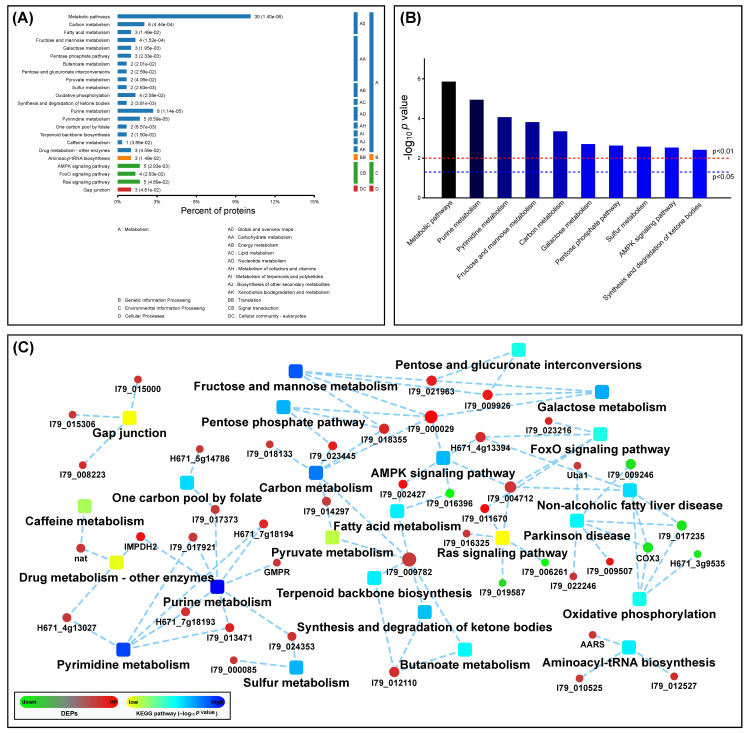
KEGG pathways with significant enrichments of DEPs in SVA-infected cells at 12 hpi. Classification of KEGG pathway enrichments (**A**). The KEGG pathways are classified into four categories: metabolism, genetic information processing, environmental information processing and cellular processes. Each bar is followed by the number of DEPs with the corresponding *p* value. Top 10 statistically significant KEGG pathways (**B**). Two *p* values, 0.05 and 0.01, are independently marked with dashed lines. Network map of interactions among DEPs (**C**). Twenty-four KEGG pathways and forty-three DEPs are marked with squares and circles, respectively. Upregulated and downregulated DEPs are marked with red and green circles, respectively.

DEPs were subjected to analysis in the KEGG database. The results (Supplementary file S7) showed that the KEGG pathways were classified into four categories: A (metabolism), B (genetic information processing), C (environmental information processing) and D (cellular processes) (Figure 5A). The metabolism was predominant in all four categories and contained the most KEGG pathways, up to 17 (not including the “metabolic pathways”). In contrast, there was only one KEGG pathway, separately identified in category B and D. The “metabolic pathways” (Pathway ID: cge01100; *p* value: 1.4 × 10^−6^) was identified to contain the highest number of DEPs, up to 30. Other important KEGG enrichments, classified into the four KEGG categories, are shown in Figure 5A. The top 10 KEGG signaling pathways contained seven metabolism-related ones (including the “metabolic pathways”) (Figure 5B), implying that the metabolic change was predominant in SVA-infected cells.

### 3.5. Protein-Protein Interaction (PPI) Analysis

As shown in Supplementary file S7, a total of 25 KEGG pathways showed their *p* values < 0.05. To clarify the relations between DEPs and KEGG paths in cells, 24 KEGG pathways (not including the “metabolic pathways”) were further analyzed by searching the STRING database (Version 11.0) [32]. The resulting network map of PPI is illustrated in Figure 5C. Out of 24 KEGG pathways, 11 were metabolism-related ones, accounting for nearly 46%. A total of 43 DEPs, including 36 upregulated and 7 downregulated ones, were identified to be related to these 24 KEGG pathways. The DEP with the highest FC was ATP-dependent 6-phosphofructokinase (gene ID: I79_000029), involved in five KEGG pathways. The purine metabolism was linked to the most DEPs, up to eight upregulated ones, and simultaneously possessed the lowest *p* value. The pyrimidine metabolism had the second lowest *p* value, and was linked to five upregulated DEPs. Out of seven downregulated DEPs, only one (gene ID: I79_016396) was related to the metabolism (fatty acid metabolism), and the other six mainly concerned non-metabolic pathways, such as the oxidative phosphorylation and Parkinson’s disease.

### 3.6. PRM of Twenty Upregulated DEPs

**Figure 6:**
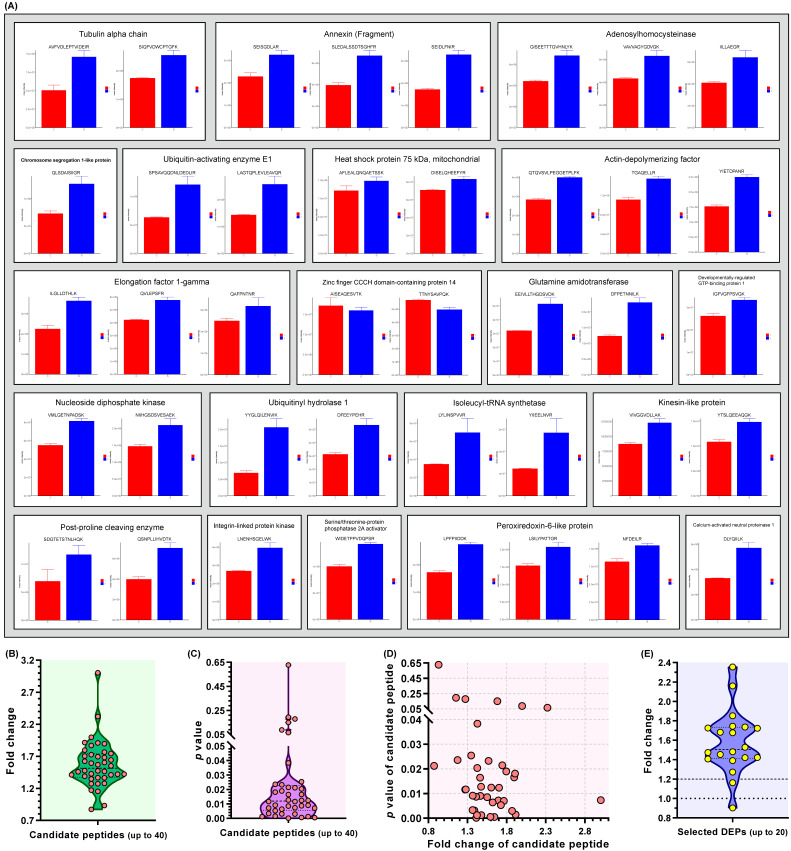
PRM analysis of 20 upregulated DEPs in SVA-infected cells at 12 hpi. Comparison of mass intensities corresponding to 40 candidate peptides between the S and C groups (**A**). Each DEP is involved in one to three candidate peptides, framed by a rectangle. Violin-plot-exhibited distributions of FC (**B**) and *p* value (**C**) for all 40 candidate peptides. Corresponding relation between FC and *p* value for each candidate peptide (**D**). Violin-plot-exhibited distribution of FC for all 20 DEPs (**E**).

PRM mass spectrometry was used to verify the target peptides found in the TMT-labeled nano-LC-MS/MS analysis of the SVA-infected cells. To confirm the proteomic data, 20 upregulated DEPs (Supplementary file S8) were selected for LC-PRM/MS analysis on all six groups. PRM-related data, including the original AUC, normalized AUC, peptide quantification, protein quantification and average basepeak, are listed in Supplementary S9. Forty optimal peptides were selected as candidates, for which Skyline analysis results are shown in Supplementary files S10 and S11. The normalized peak area of peptide segment was used to analyze quantitatively the target peptide segments from different samples. Mass intensities of 40 candidate peptides were compared between the S and C groups (Figure 6A). Figure 6B,C represented actual distributions of FC and *p* values for these 40 candidate peptides, respectively. Figure 6D showed the relation between both parameters of each candidate peptide. The PRM quantitative results (Supplementary file S9, Sheet-protein quantification) showed similar trends to those of TMT-based proteomic analysis on 19 candidate proteins, except the zinc finger CCCH domain-containing protein 14 (gene ID: I79_007785), whose FC was less than 1.0 (Figure 6E), implying that the TMT-based proteomic data were reliable. Out of the 19 DEPs that were confirmed to be upregulated, the heat shock protein 75 kDa (mitochondrial) (gene ID: I79_016693) revealed its FC value to be less than 1.2 (Figure 6E) but still with the upregulated trend.

## 4. Discussion

SVA is an emerging virus that has recently affected numerous pig farms in China [33,34,35,36]. The SVA CH-LX-01-2016 was a China isolate [26], for which a reverse genetics system was developed in our earlier study. Using this system, we rescued one wild-type [24] and two reporter-tagged [37,38] SVAs. The wild-type SVA, initially recovered from its cDNA clone, contained no point mutation at P0. Even though the P0 SVA was serially passaged in vitro for 10 times, there were only four single-nucleotide polymorphisms identified in viral genome [24]. Therefore, the P5 SVA stock was speculated to contain no more than four low-frequency point mutations. In other words, it was a relatively “pure” stock without its own quasispecies. Due to no impact of viral quasispecies at P5 on cells, it was appropriate for the analysis of intracellular proteome after virus infection. Prior to SVA inoculation, the BSR-T7/5 cells were additionally subjected to PCR detection to confirm no mycoplasma contamination in cell culture (data not shown). SVA-induced robust CPEs, like cell rounding, lysis and detachment, can appear as early as 24 hpi, or even earlier [24]. In order to reduce the interference of CPEs with protein profiling, cell samples were collected from both S and C groups as early as 12 hpi.

Proteomic analysis plays a crucial role in revealing cellular metabolisms, viral replication, antiviral responses, and virus–host interactions at the protein level [21,39,40]. Using the technique of TMT-labeled nano-LC-MS/MS in this study, a total of 5301 proteins were identified and quantified across the six groups (Supplementary file S1). Most of these proteins showed their molecular weights ranging from 20 to 150 kD (Figure 2A). Comparative proteomic analysis indicated 361 DEPs, including 305 upregulated and 56 downregulated ones in cells at 12 hpi. The BP, CC and MF were quantified by the GO annotation and enrichment analysis, suggesting that DEPs were significantly enriched (*p* < 0.05) in BP and MF. KEGG pathway annotation and enrichment analysis revealed that a total of 25 statistically significant pathways were identified (Supplementary file S7), and DEPs were mainly enriched in the metabolisms, up to 11, including purine, pyrimidine, fructose and mannose, carbon, galactose, sulfur, fatty acid, butanoate, caffeine, pyruvate and drug (other enzymes) metabolisms. Based on the results of GO and KEGG enrichment analyses, it can be concluded that cellular metabolisms are mainly affected in SVA-infected cells at 12 hpi.

The TMT as a chemical label facilitates sample multiplexing in MS-based quantification and identification of biological macromolecules [41]. The TMT-labeled nano-LC-MS/MS facilitated our comparative proteomic analysis between SVA- and non-infected cells. More recently, Li et al. (2020) used another method, iTRAQ-labeled LS-MS/MS, to analyze comparatively the proteomes between SVA (isolate, SVV-CH-SD)- and non-infected testicular cells at 24 hpi [11]. Since they use a different labeling strategy, SVA strain, cell line and hpi from those in our current study, proteomic data are incomparable between these two studies. Besides the proteomic analysis, Wang et al. (2020) recently reported a transcriptomic profile of SVA-infected pig kidney cells. Type I interferons were demonstrated to be upregulated at the highest level, implying that they played a critical role in immune responses against SVA infection at an early stage in a host [42]. However, we did not observe a similar result concerning the significant upregulation of type I interferons, possibly attributed to the non-pig-derived cell line used for SVA propagation in our study.

The PRM assay has emerged as an alternative method of targeted quantification. It can be performed both in the high resolution and in the high mass accuracy mode on a mass spectrometer [43]. In this study, the PRM technique was used for analysis of 20 upregulated DEPs with high FC values to verify the proteomic profile. Nineteen candidate DEPs were demonstrated to have similar trends to those of the TMT-labeled nano-LC-MS/MS analysis, suggesting that the comparative proteomic data were reliable and could be used for further research. A total of 305 upregulated and 56 downregulated DEPs were identified in the TMT-labeled nano-LC-MS/MS analysis. Due to lack of interesting proteins in the list of downregulated DEPs, we did not perform the PRM analysis on downregulated DEPs. Out of all DEPs, the glyceraldehyde-3-phosphate dehydrogenase (GAPDH) was found to be upregulated (Supplementary file S2, FC value: 1.30; *p* value: 0.0015). In general, the GAPDH is known to be stably expressed at high levels in most tissues and cells and is therefore widely used as an internal control for Western blotting. Interestingly, our study demonstrated that the GAPDH was an unreliable internal control for the comparative analysis on protein quantification of BSR-T7/5 cells infected with SVA.

To sum up, in the present study, the TMT-labeled nano-LC-MS/MS was used to uncover the proteomic atlases of SVA- and non-infected cells at the early stage of infection. The LC-PRM/MS subsequently confirmed that the proteomic data were reliable. The comparative proteomic analysis revealed a total of 305 upregulated and 56 downregulated DEPs. Out of these, a large number of DEPs were involved in cellular metabolisms, suggesting that SVA, at its early stage of infection, mainly interferes with various metabolic pathways in BSR-T7/5 cells. An integrated metabolic atlas remains to be explored via metabolomic methods.

## Data Availability

The datasets supporting the conclusions of this article are included within the article and its additional files. The mass spectrometry proteomics data have been deposited to the ProteomeXchange Consortium via the PRIDE partner repository with the dataset identifier PXD024947.

## Supplementary Materials

The following are available online at https://www.mdpi.com/article/10.3390/v13061036/s1, Supplementary file S1: Proteomic profile, Supplementary file S2: Upregulated DEPs, Supplementary file S3: Downregulated DEPs, Supplementary file S4: Biological process, Supplementary file S5: Cell component, Supplementary file S6: molecular function, Supplementary S7: KEGG pathways, Supplementary file S8: Upregulated DEPs, Supplementary file S9: PRM-related data, Supplementary file S10: Secondary spectra of candidate peptides, Supplementary file S11: Skyline analyses of candidate peptides.
