# Supplementary material for: Comparative Proteomic Profiling: Cellular Metabolisms Are Mainly Affected in Senecavirus A-Inoculated Cells at an Early Stage of Infection
Source: Viruses. 2021 May 31;13(6):1036. doi: 10.3390/v13061036 (PMC8226903; doi:10.3390/v13061036)
Supplement: Supplementary file 1 [file viruses-13-01036-s001.zip › viruses-1201879-supplementary/Supplementary S10.pdf]

Secondary spectra of candidate peptides

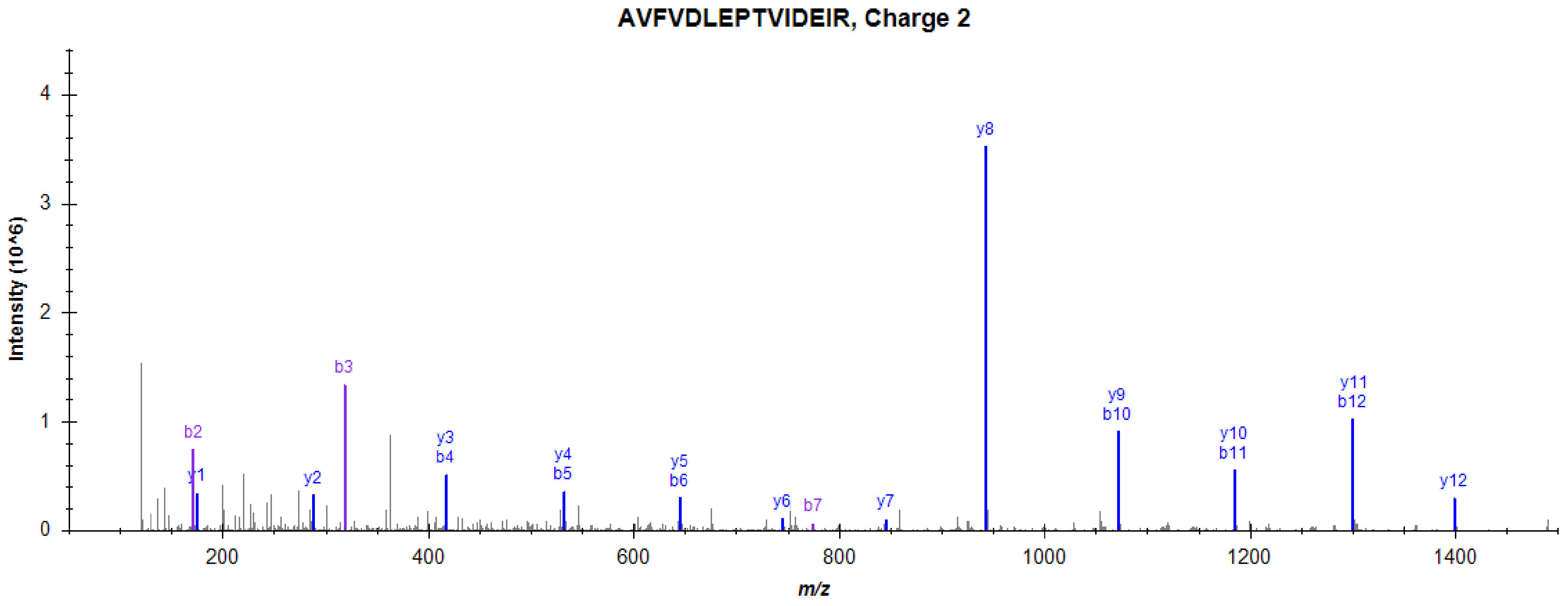

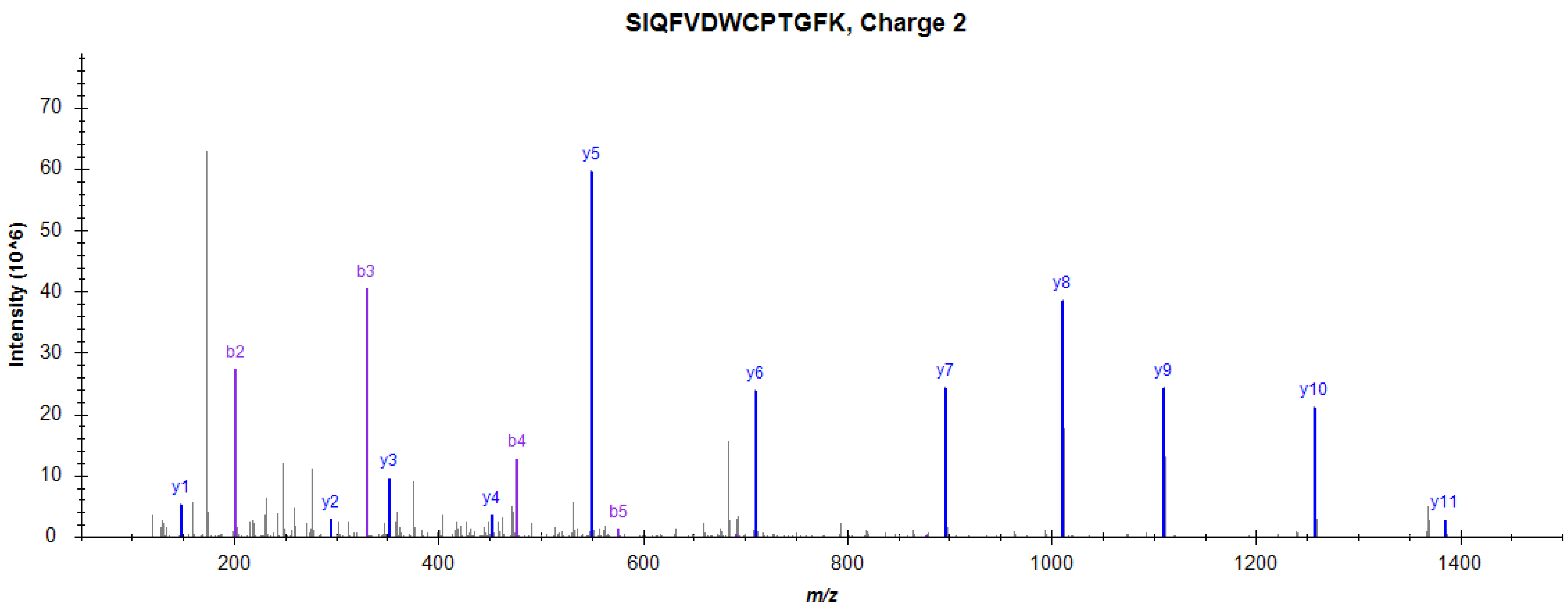

SIQFVDWCPTGFK, Charge 2
Intensity (10^6)
m/z
y1
b2
y2
b3
y3
y4
b4
y5
b5
y6
y7
y8
y9
y10
y11
0
10
20
30
40
50
60
70
200
400
600
800
1000
1200
1400

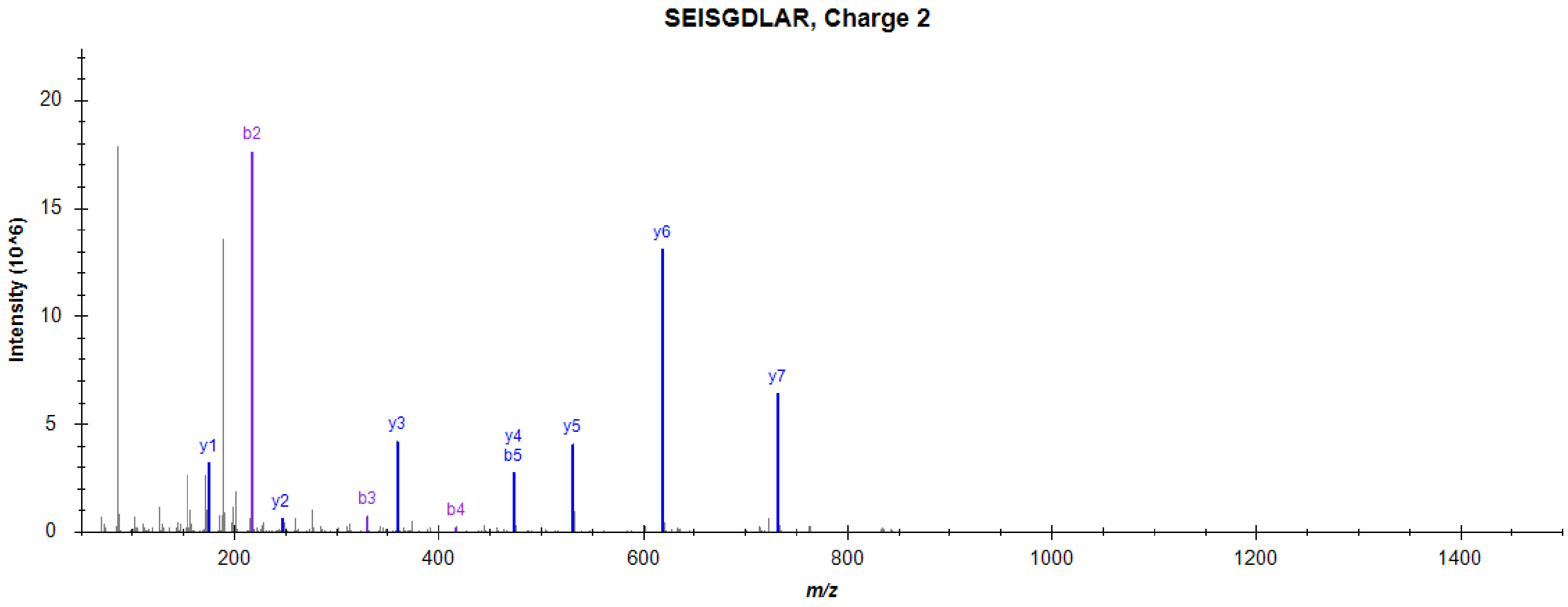

SEISGDLAR, Charge 2
Intensity (10^6)
m/z
b2
y1
y2
b3
y3
b4
y4
b5
y5
y6
y7
0
5
10
15
20
200
400
600
800
1000
1200
1400

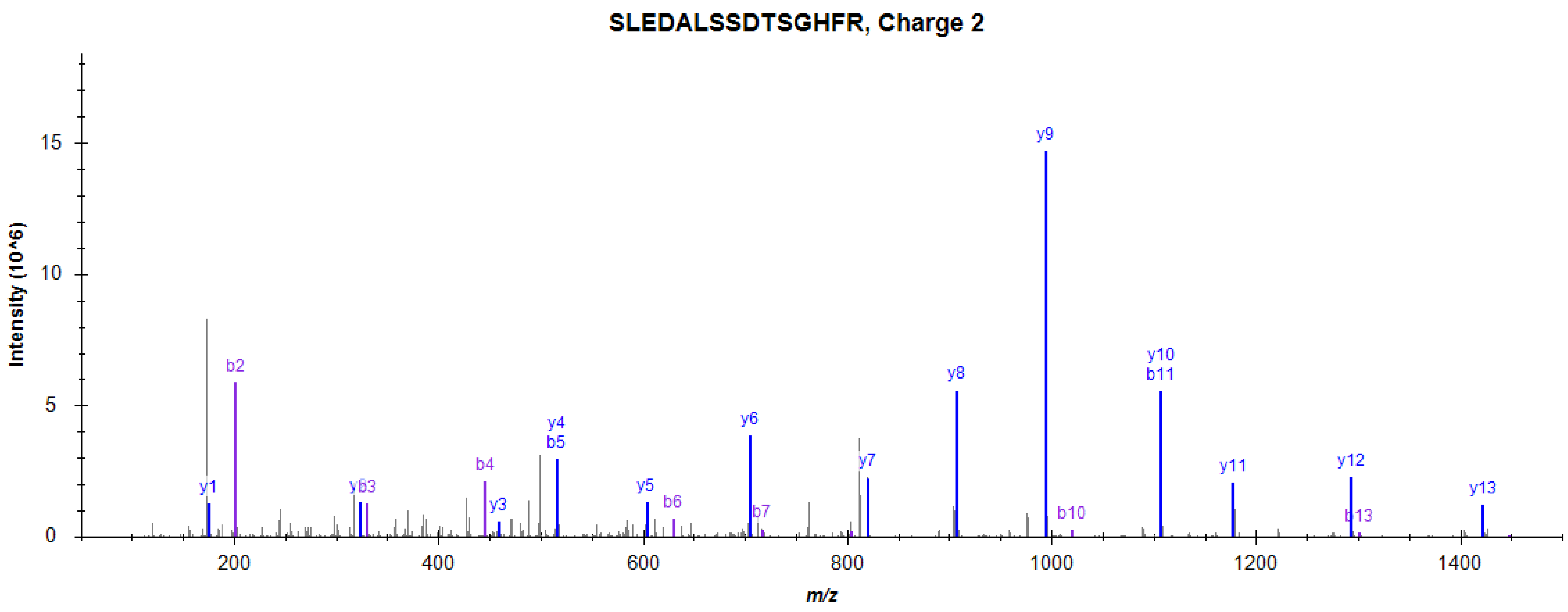
SLEDALSSDTSGHFR, Charge 2
Intensity (10^6)
m/z
0
5
10
15
200
400
600
800
1000
1200
1400
y1
b2
b3
b4
y3
y4
b5
y5
b6
y6
b7
y7
y8
y9
b10
y10
b11
y11
y12
b13
y13

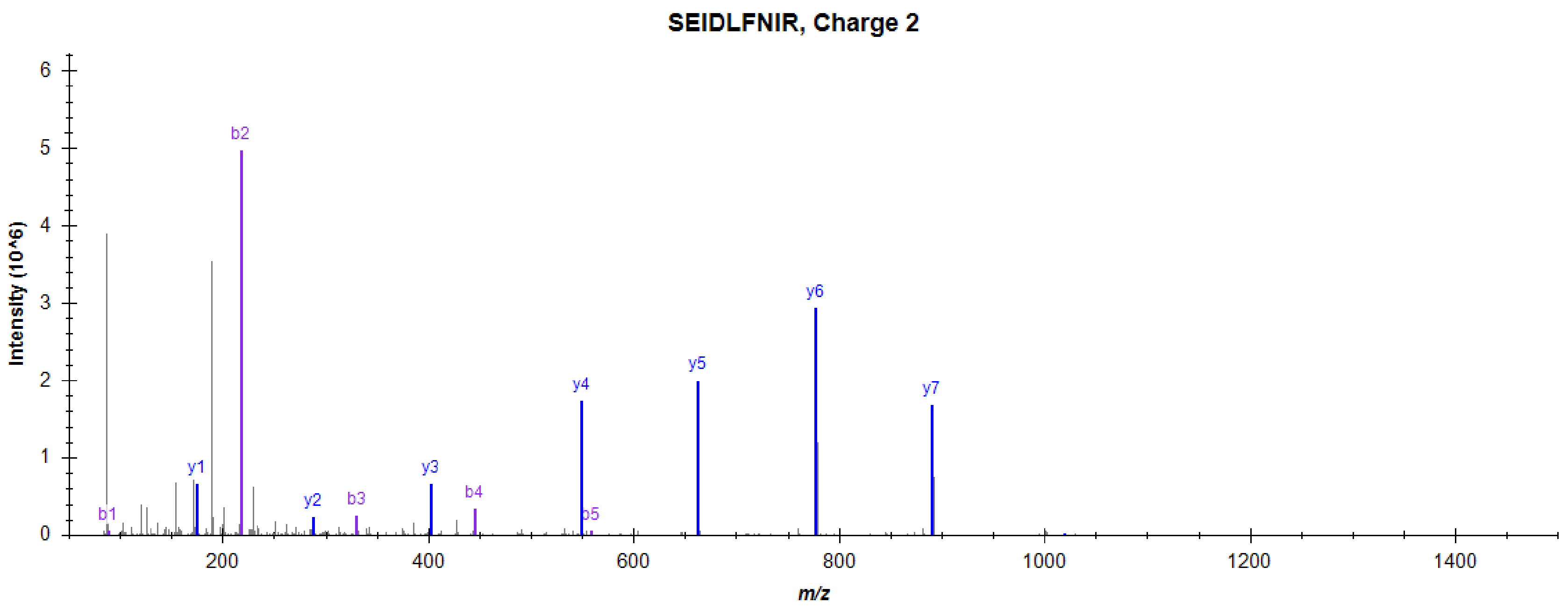
SEIDLFNIR, Charge 2
Intensity (10^6)
m/z
0
1
2
3
4
5
6
200
400
600
800
1000
1200
1400
b1
y1
b2
y2
b3
y3
b4
y4
b5
y5
y6
y7

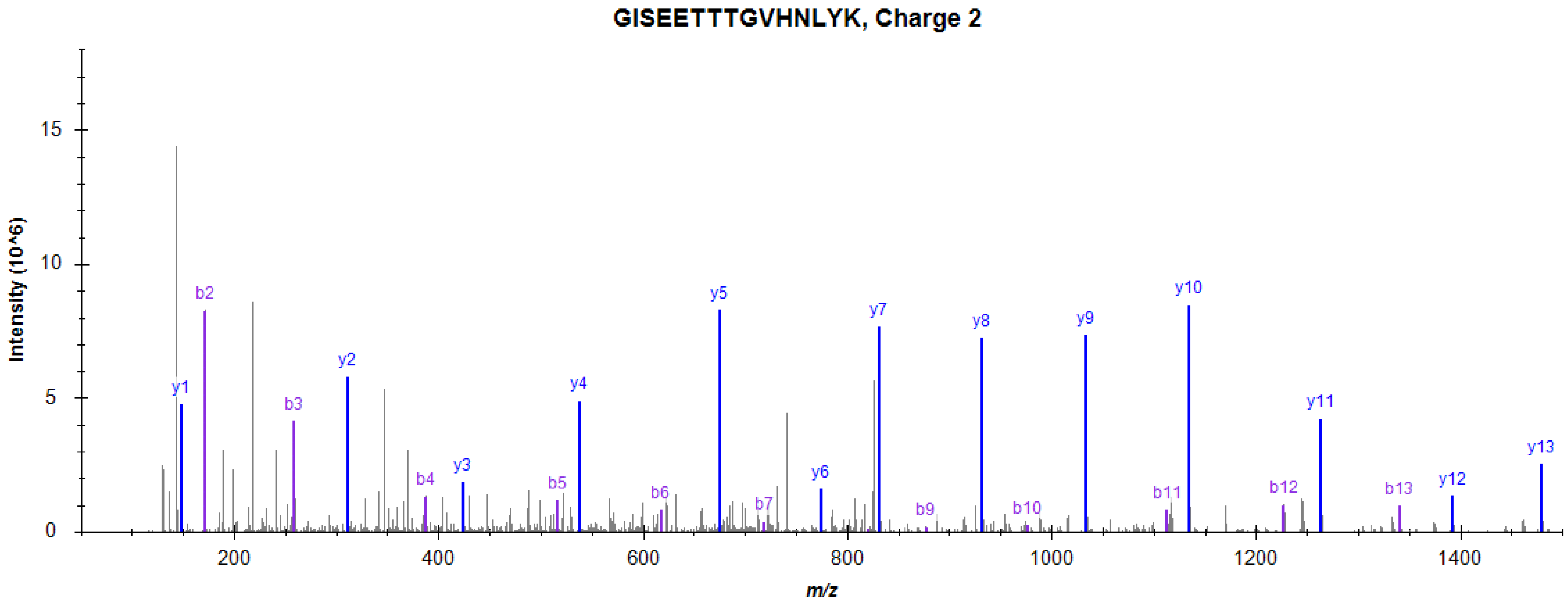

GISEETTTGVHNLYK, Charge 2
Intensity (10^6)
m/z
0
5
10
15
200
400
600
800
1000
1200
1400
y1
b2
b3
y2
b4
y3
y4
b5
b6
y5
b7
y6
y7
b9
y8
b10
y9
b11
y10
b12
y11
b13
y12
y13

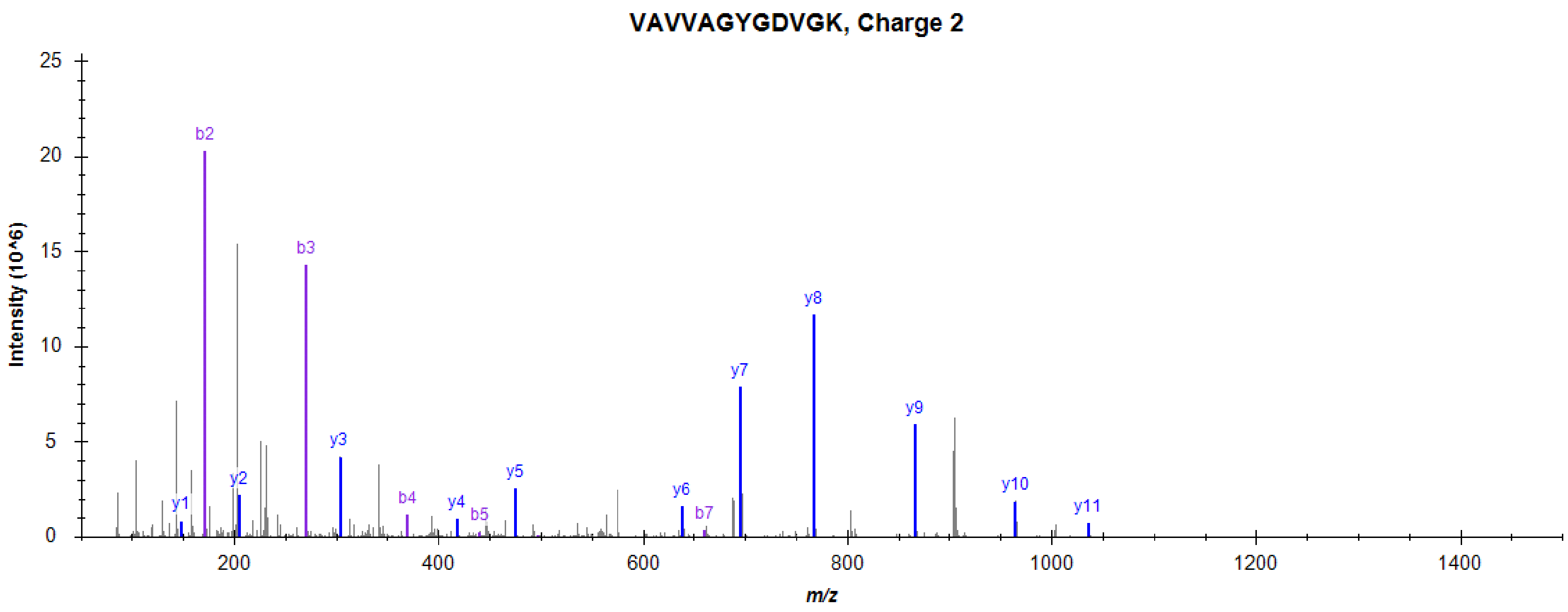

VAVVAGYGDVGK, Charge 2
Intensity (10^6)
m/z
b2
b3
b4
b5
b7
y1
y2
y3
y4
y5
y6
y7
y8
y9
y10
y11
0
5
10
15
20
25
200
400
600
800
1000
1200
1400

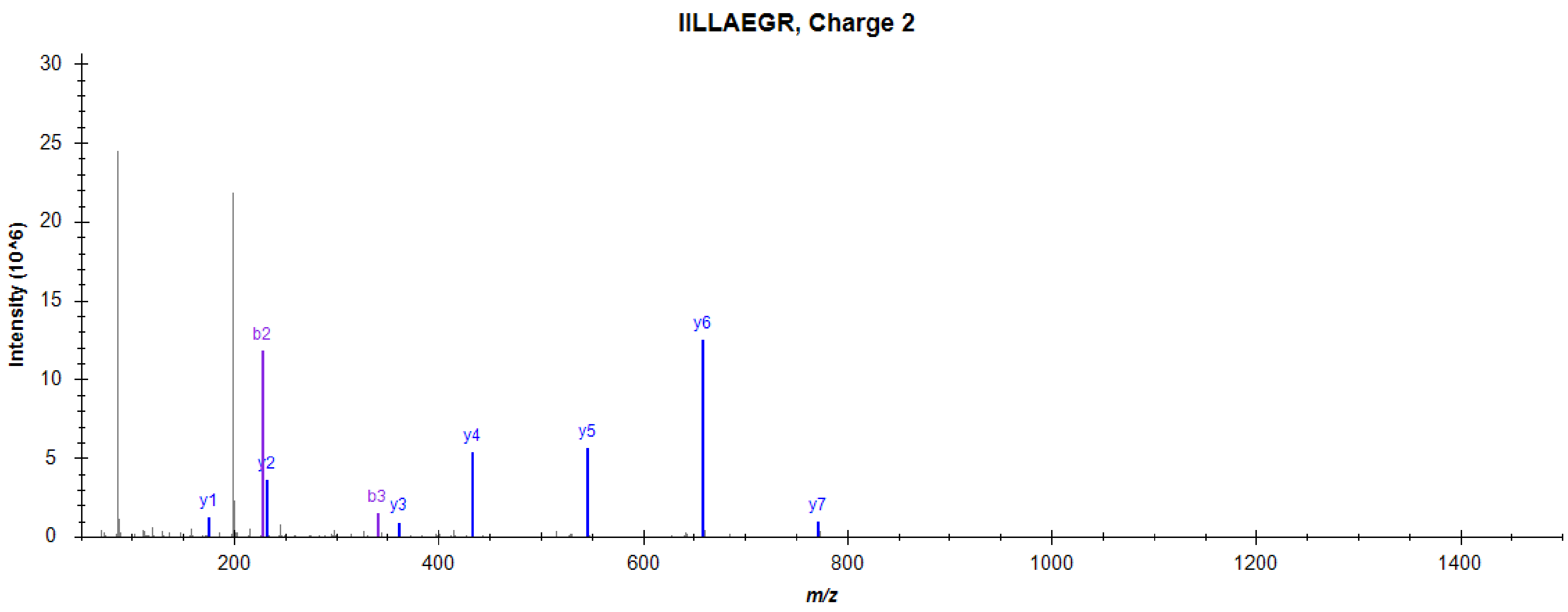
IILLAEGR, Charge 2
Intensity (10^6)
m/z
b2
y1
y2
b3
y3
y4
y5
y6
y7
0
5
10
15
20
25
30
200
400
600
800
1000
1200
1400

QLSDAISIIGR, Charge 2

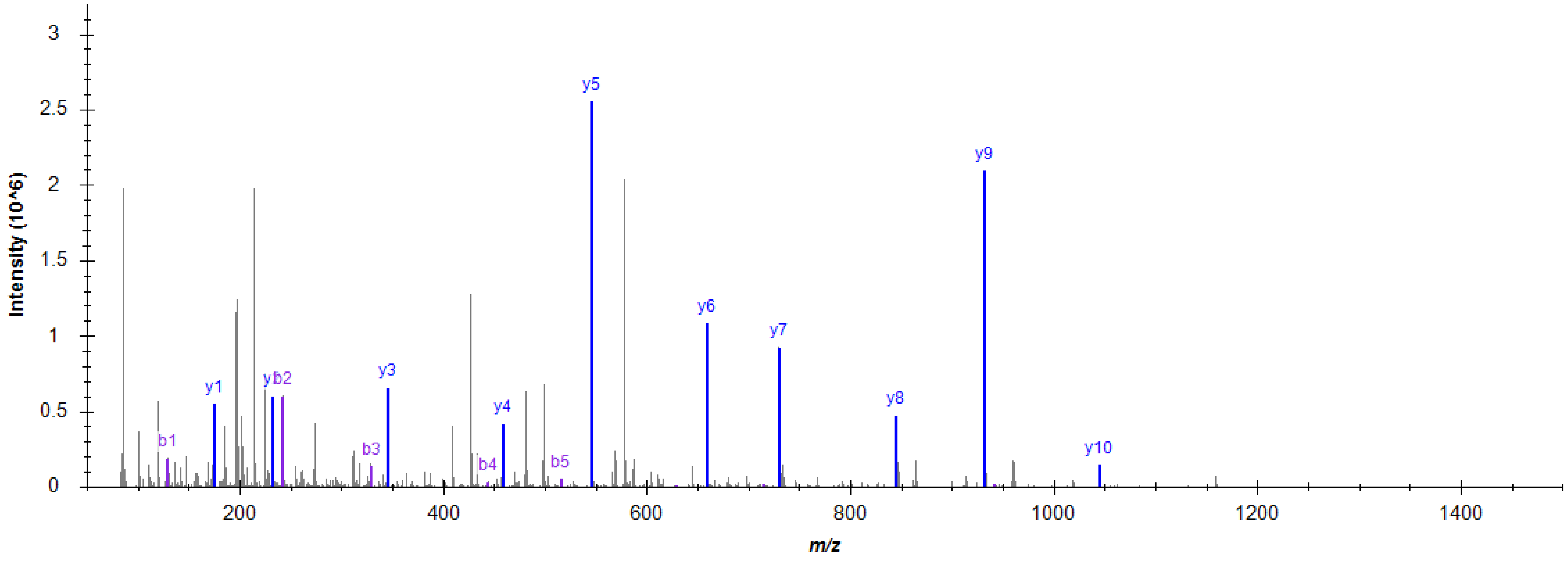

SPSAVQQDNLDEDLIR, Charge 2

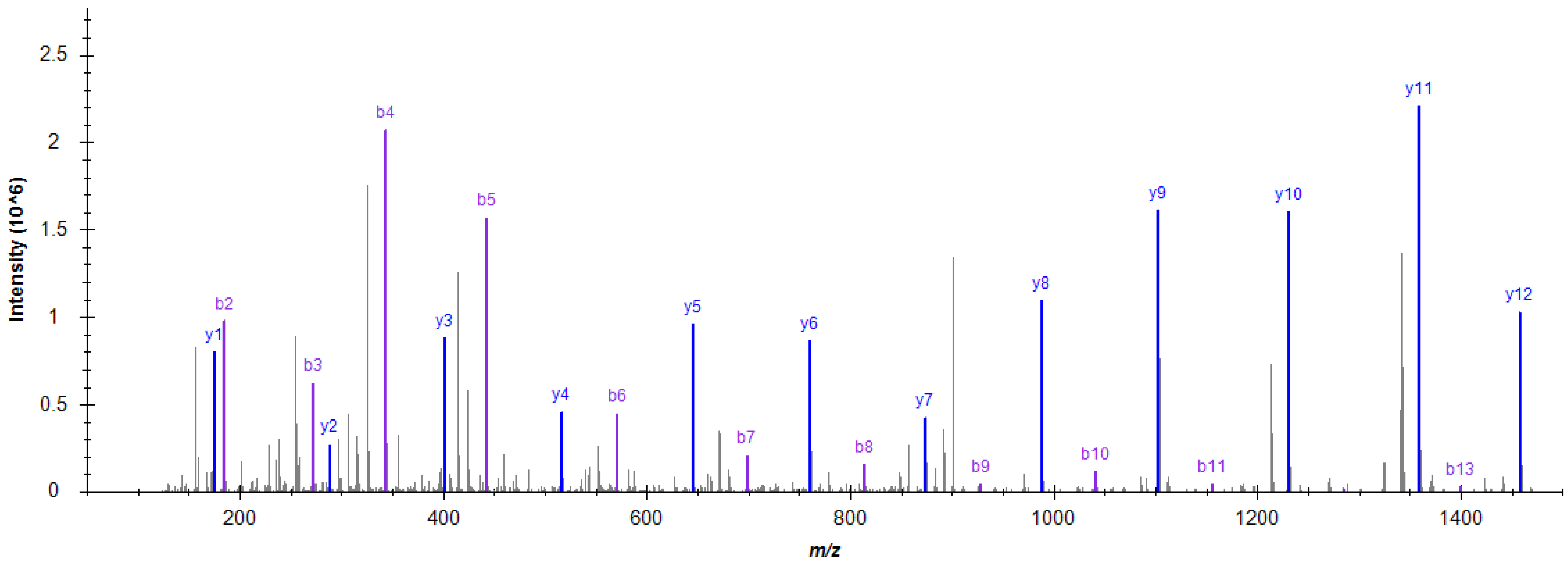

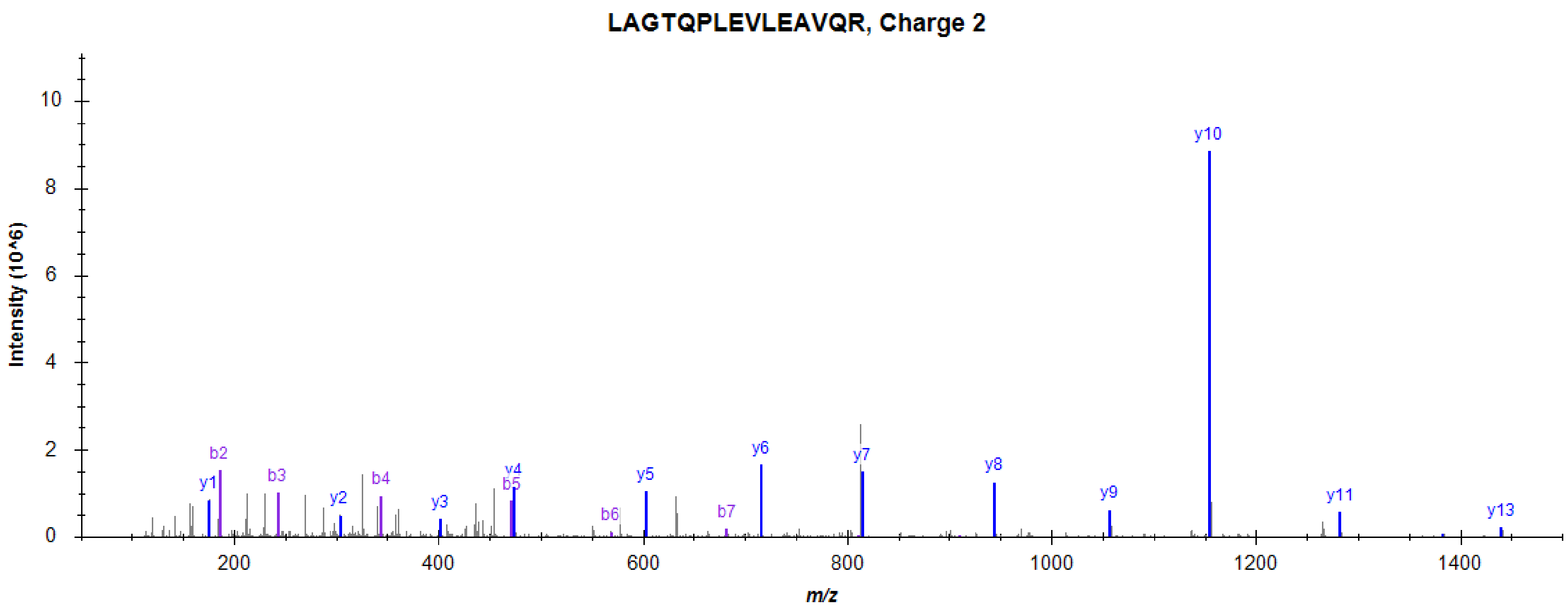
LAGTQPLEVLEAVQR, Charge 2
Intensity (10^6)
m/z
0
2
4
6
8
10
200
400
600
800
1000
1200
1400
y1
b2
b3
y2
b4
y3
y4
b5
b6
y5
y6
b7
y7
y8
y9
y10
y11
y13

AFLEALQNQAETSSK, Charge 2

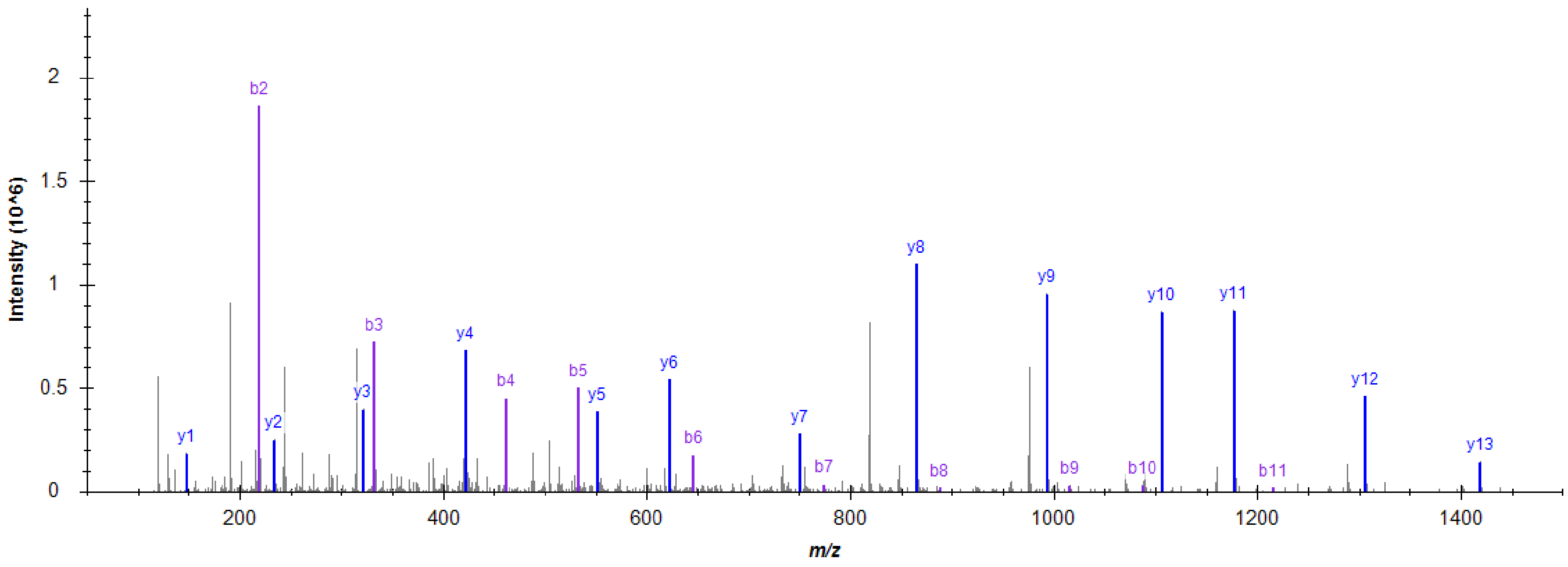

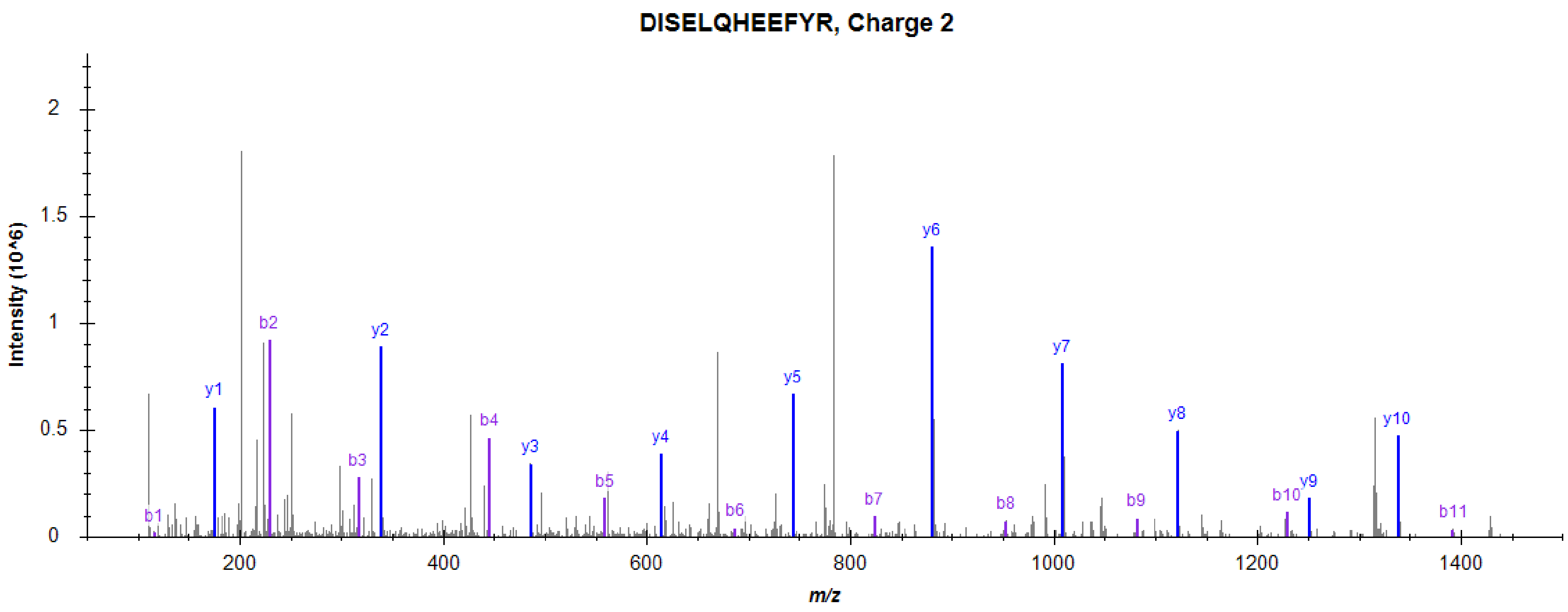
DISELQHEEFYR, Charge 2
Intensity (10^6)
m/z
0
0.5
1
1.5
2
200
400
600
800
1000
1200
1400
y1
y2
y3
y4
y5
y6
y7
y8
y9
y10
b1
b2
b3
b4
b5
b6
b7
b8
b9
b10
b11

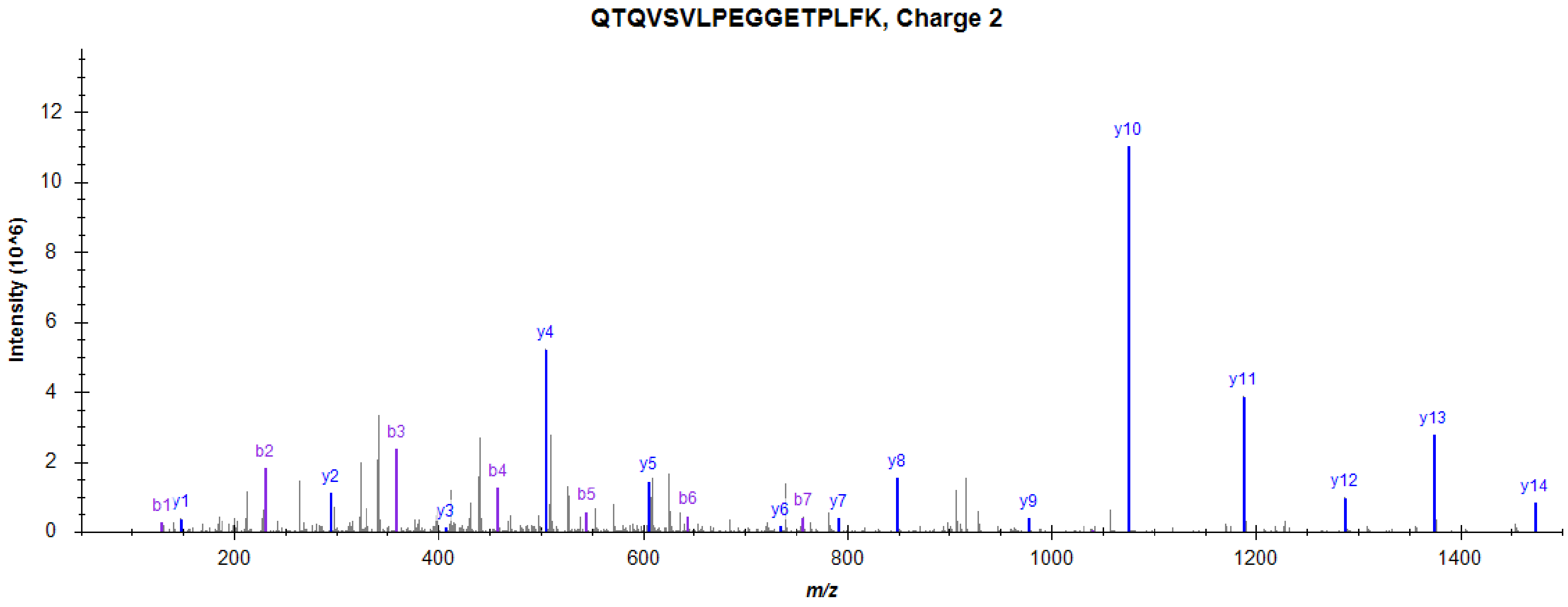
QTQVSVLPEGGETPLFK, Charge 2
Intensity (10^6)
m/z
0
2
4
6
8
10
12
200
400
600
800
1000
1200
1400
b1
y1
b2
y2
b3
y3
b4
y4
b5
y5
b6
y6
b7
y7
y8
y9
y10
y11
y12
y13
y14

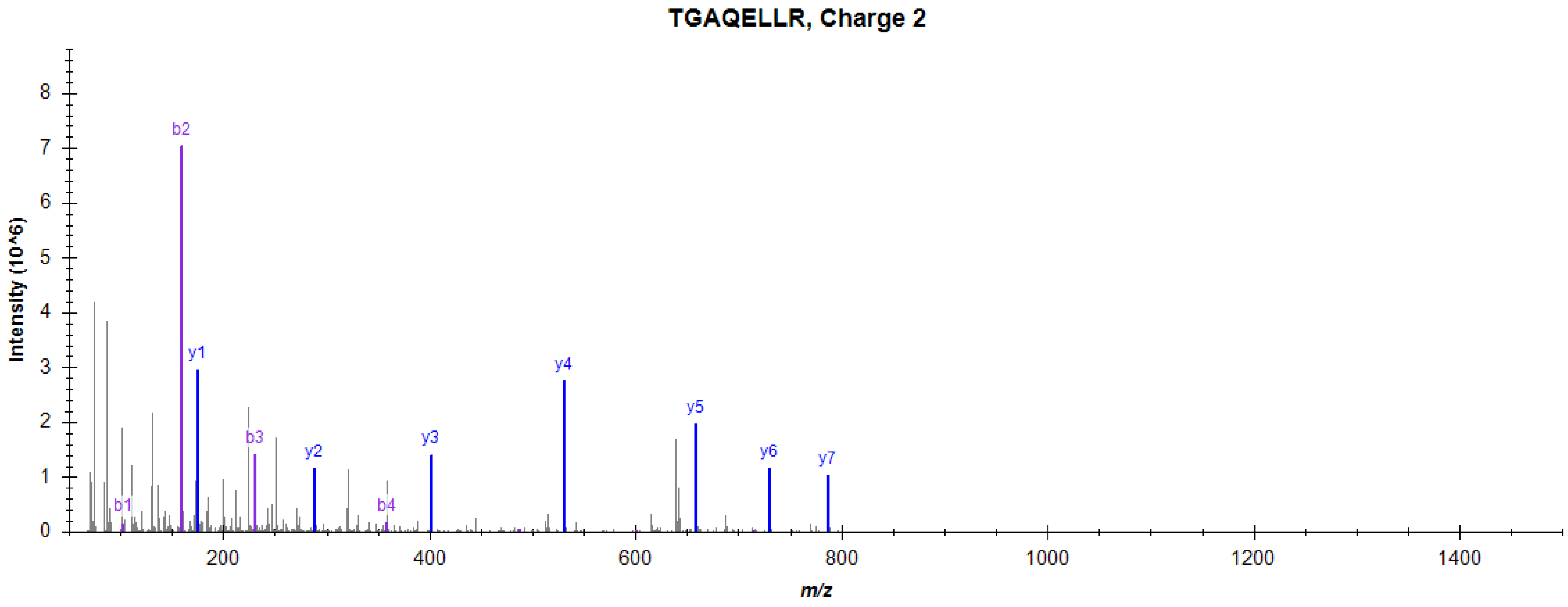
TGAQELLR, Charge 2
Intensity (10^6)
m/z
b1
b2
b3
b4
y1
y2
y3
y4
y5
y6
y7
0
1
2
3
4
5
6
7
8
200
400
600
800
1000
1200
1400

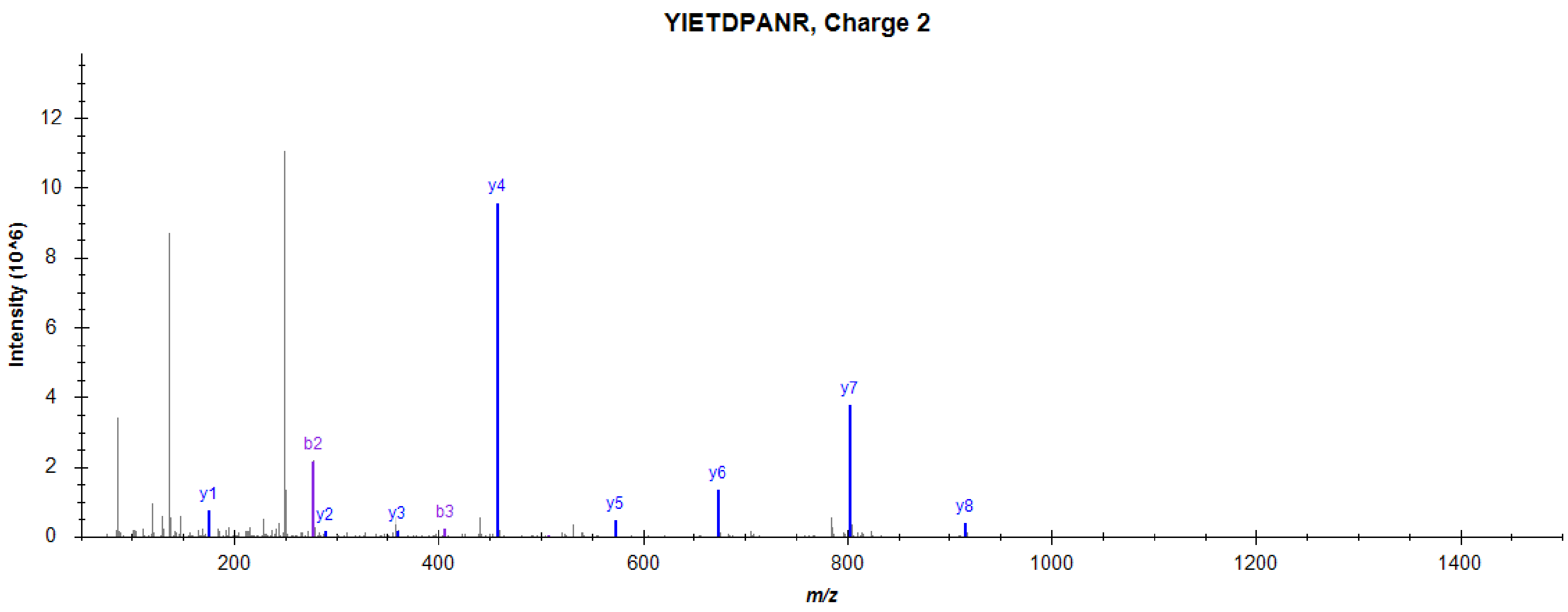
YIETDPANR, Charge 2
Intensity (10^6)
m/z
y1
y2
y3
b2
b3
y4
y5
y6
y7
y8
0
2
4
6
8
10
12
200
400
600
800
1000
1200
1400

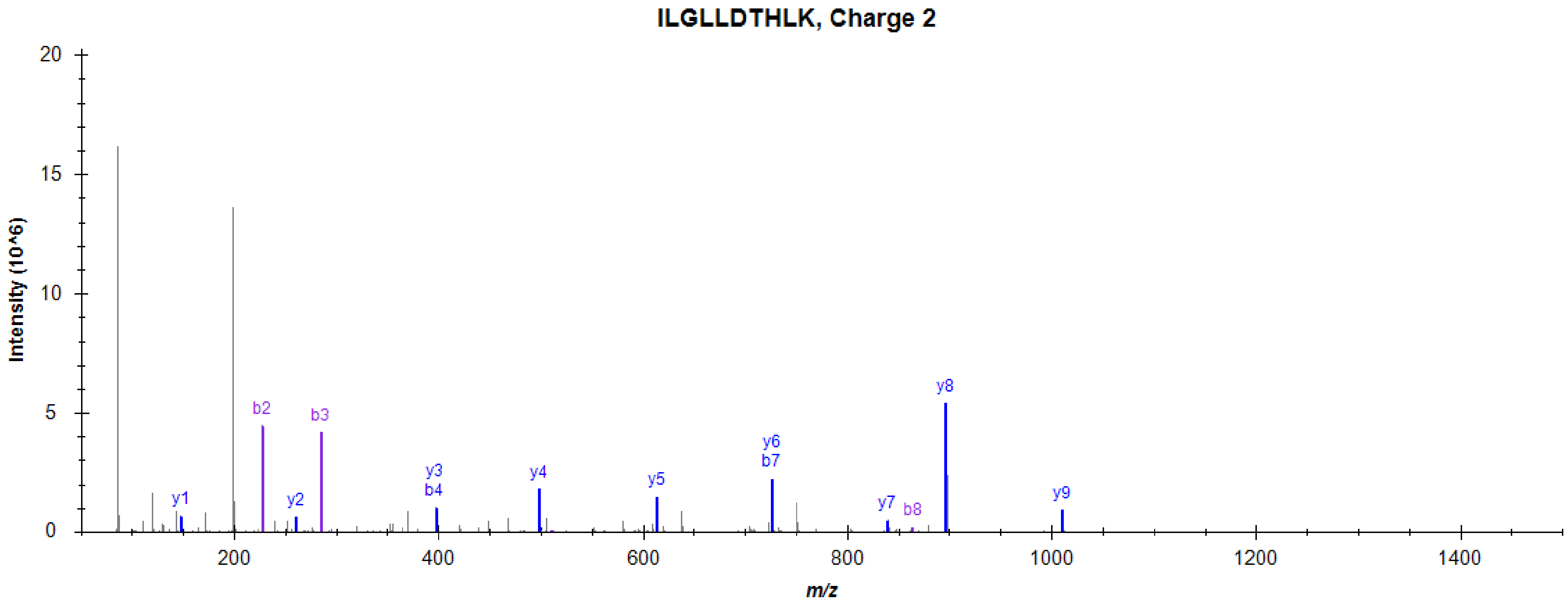

ILGLLDTHLK, Charge 2
Intensity (10^6)
m/z
20
15
10
5
0
200
400
600
800
1000
1200
1400
b2
b3
y1
y2
y3
b4
y4
y5
y6
b7
y7
b8
y8
y9

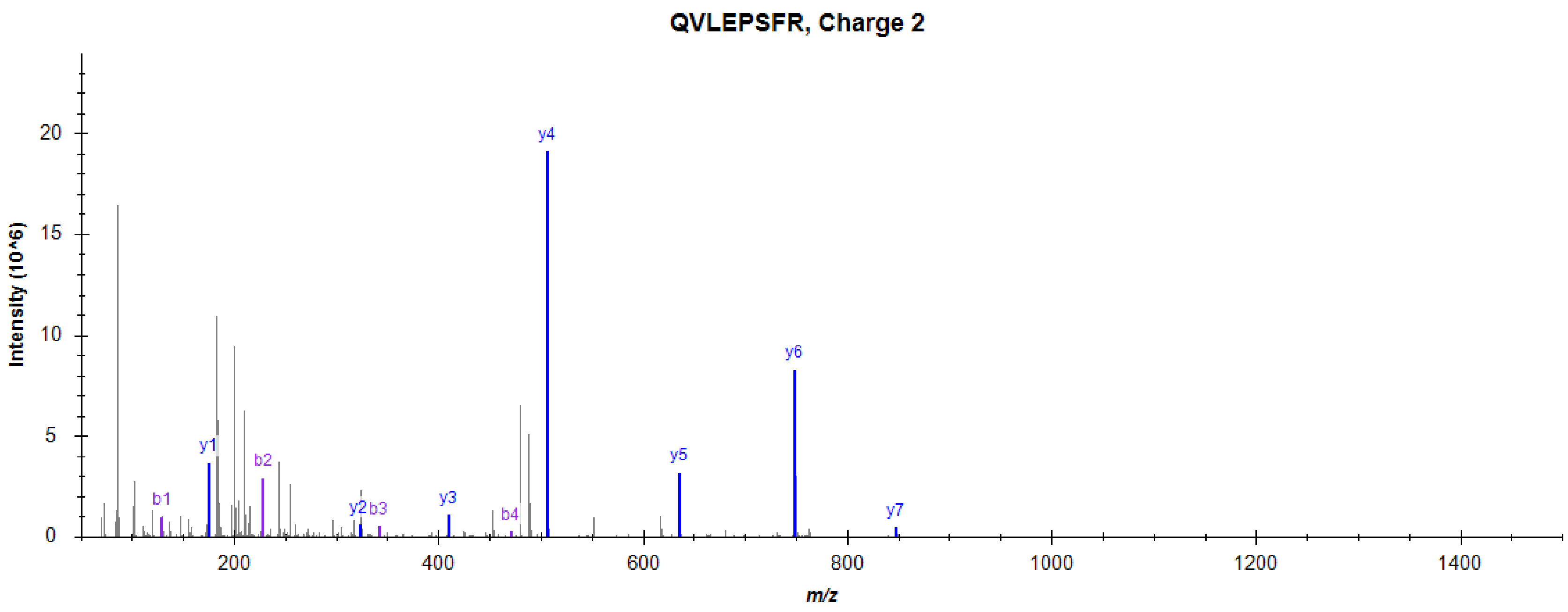
QVLEPSFR, Charge 2
Intensity (10^6)
m/z
0
5
10
15
20
200
400
600
800
1000
1200
1400
b1
y1
b2
y2
b3
y3
b4
y4
y5
y6
y7

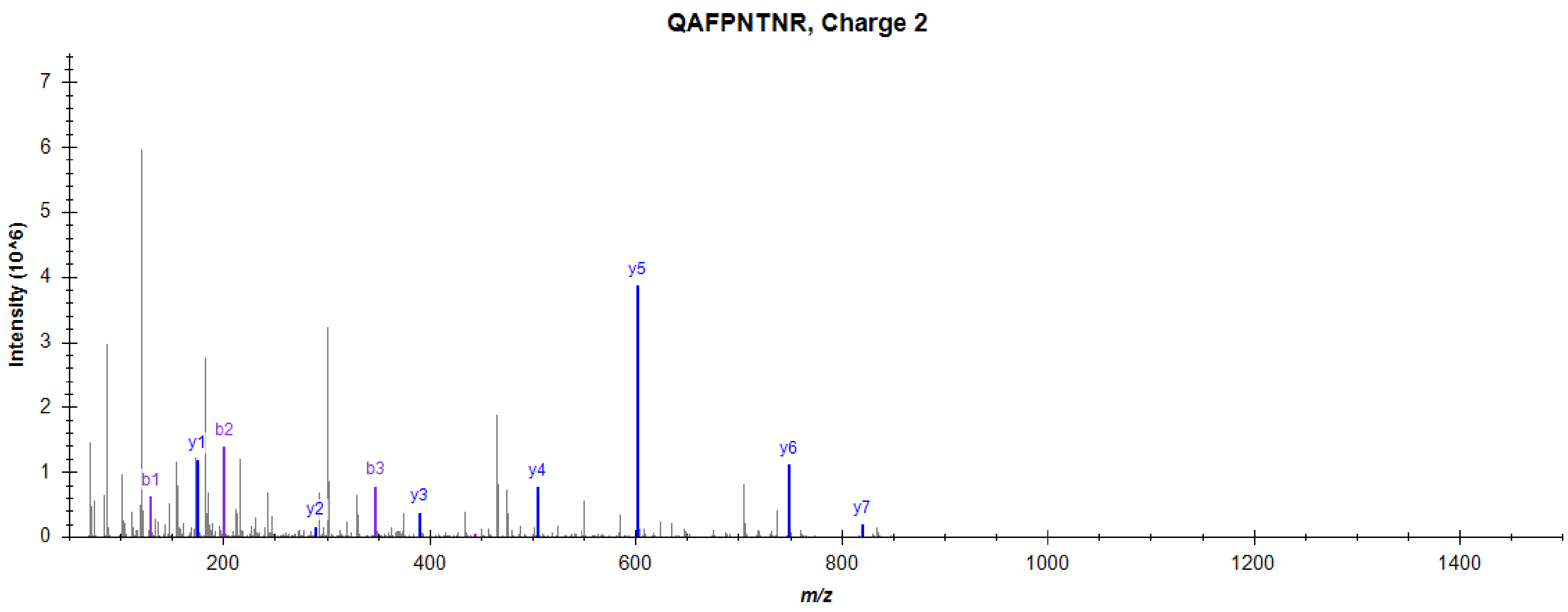
QAFPNTNR, Charge 2
Intensity (10^6)
m/z
0
1
2
3
4
5
6
7
200
400
600
800
1000
1200
1400
b1
y1
b2
y2
b3
y3
y4
y5
y6
y7

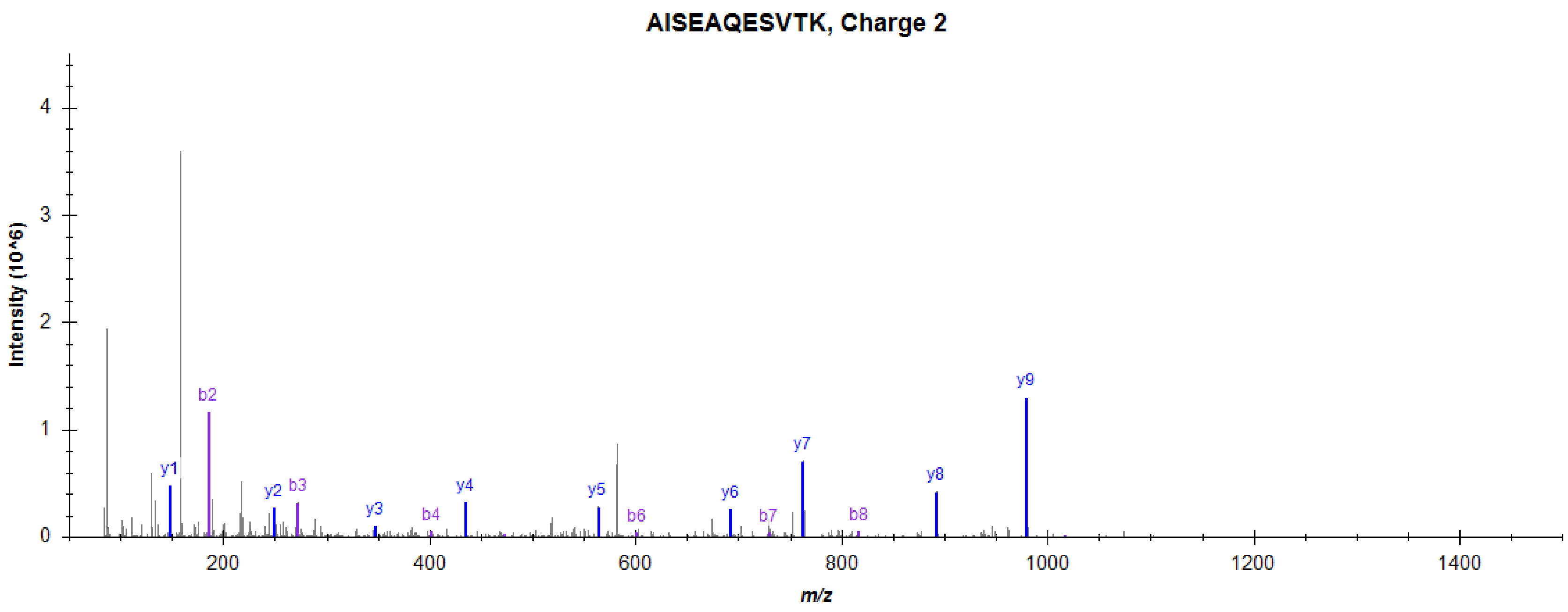
AISEAQESVTK, Charge 2
Intensity (10^6)
m/z
0
1
2
3
4
200
400
600
800
1000
1200
1400
y1
b2
y2
b3
y3
b4
y4
y5
b6
y6
b7
y7
b8
y8
y9

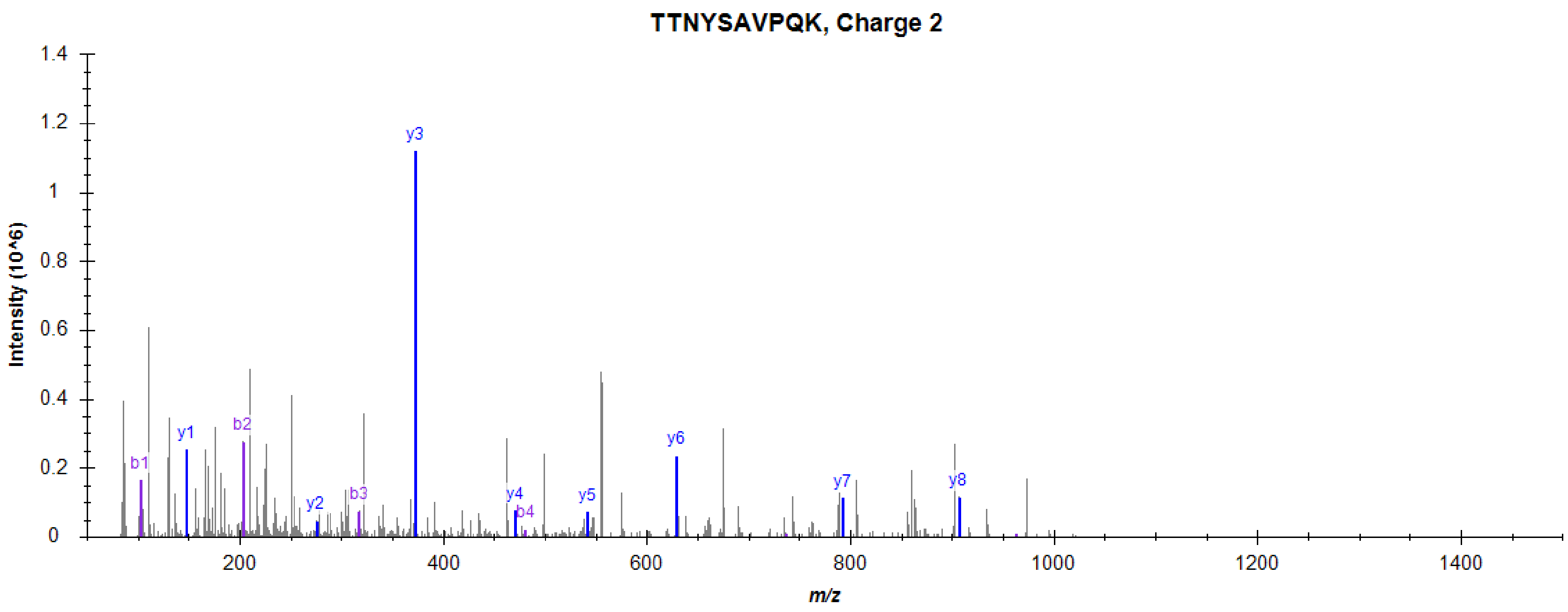
TTNYSAVPQK, Charge 2
Intensity (10^6)
m/z
y3
y1
b2
b1
y2
b3
y4
b4
y5
y6
y7
y8
0
0.2
0.4
0.6
0.8
1
1.2
1.4
200
400
600
800
1000
1200
1400

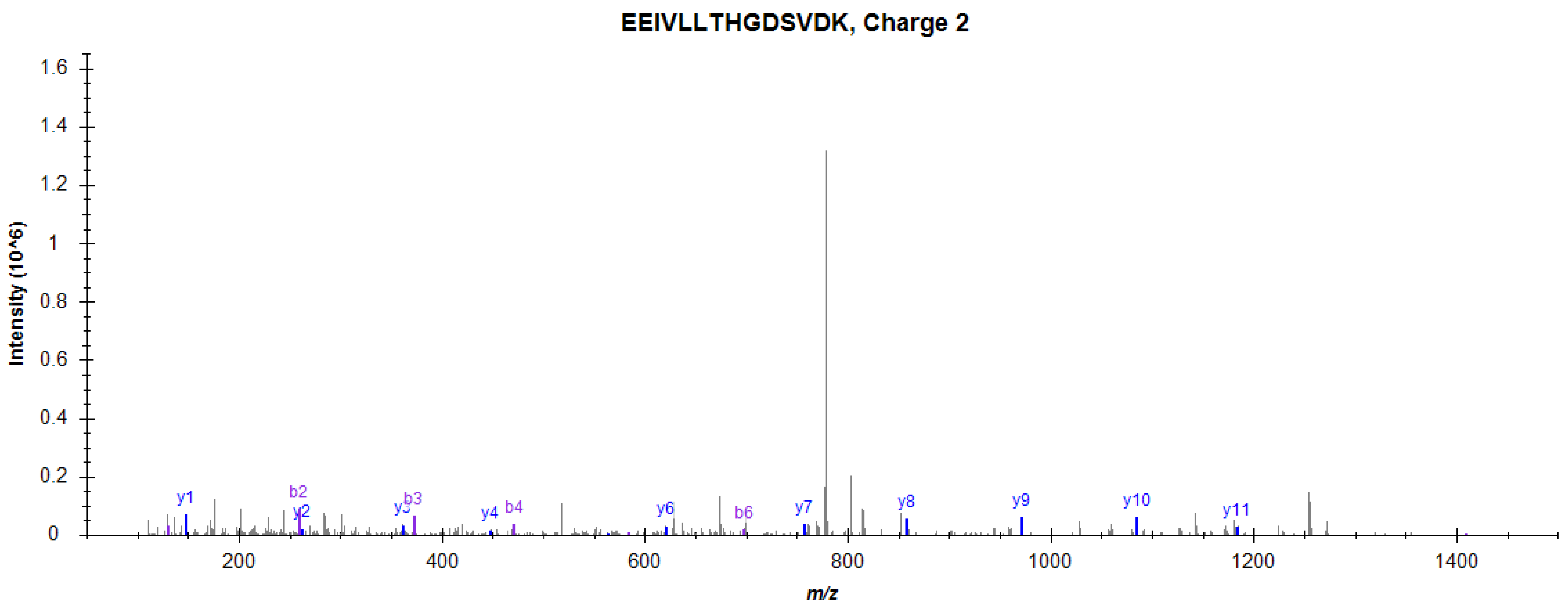

EEIVLLTHGDSVDK, Charge 2
Intensity (10^6)
m/z
y1
b2
y2
y3
b3
y4
b4
y6
b6
y7
y8
y9
y10
y11

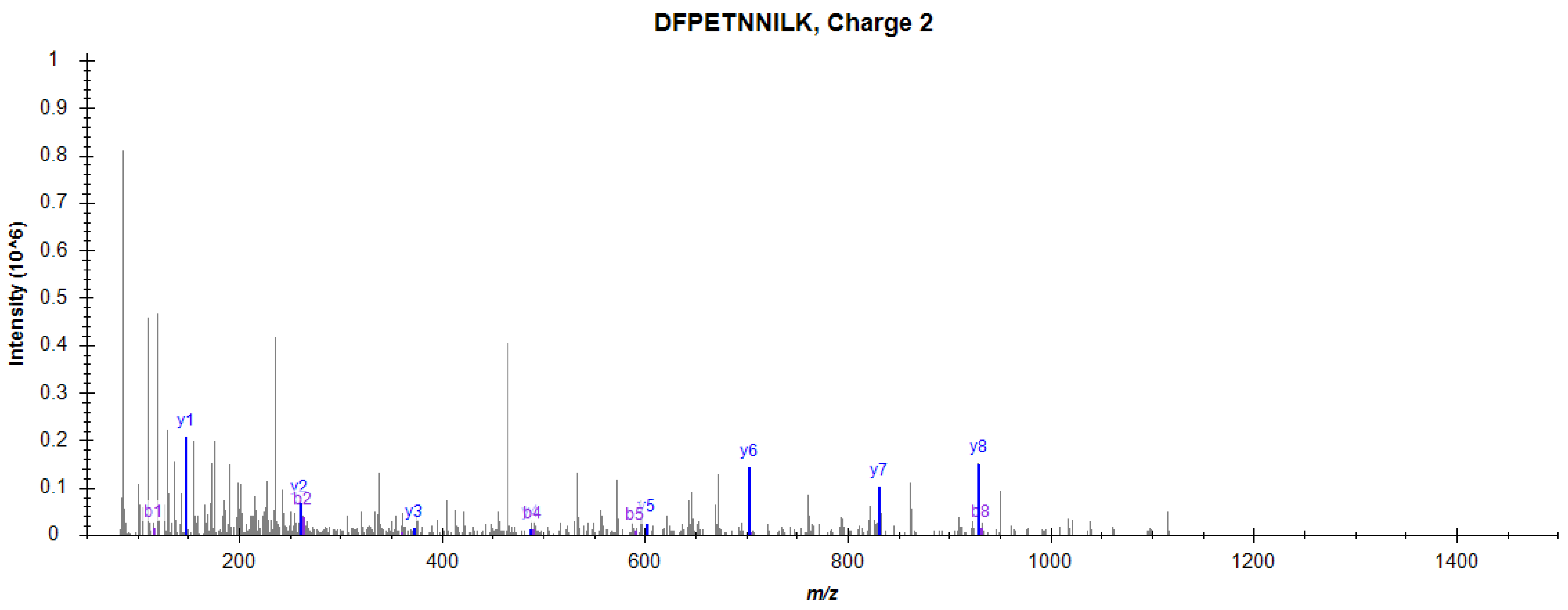
DFPETNNILK, Charge 2
Intensity (10^6)
1
0.9
0.8
0.7
0.6
0.5
0.4
0.3
0.2
0.1
0
y1
b1
y2
b2
y3
b4
b5
y5
y6
y7
y8
b8
200
400
600
800
1000
1200
1400
m/z

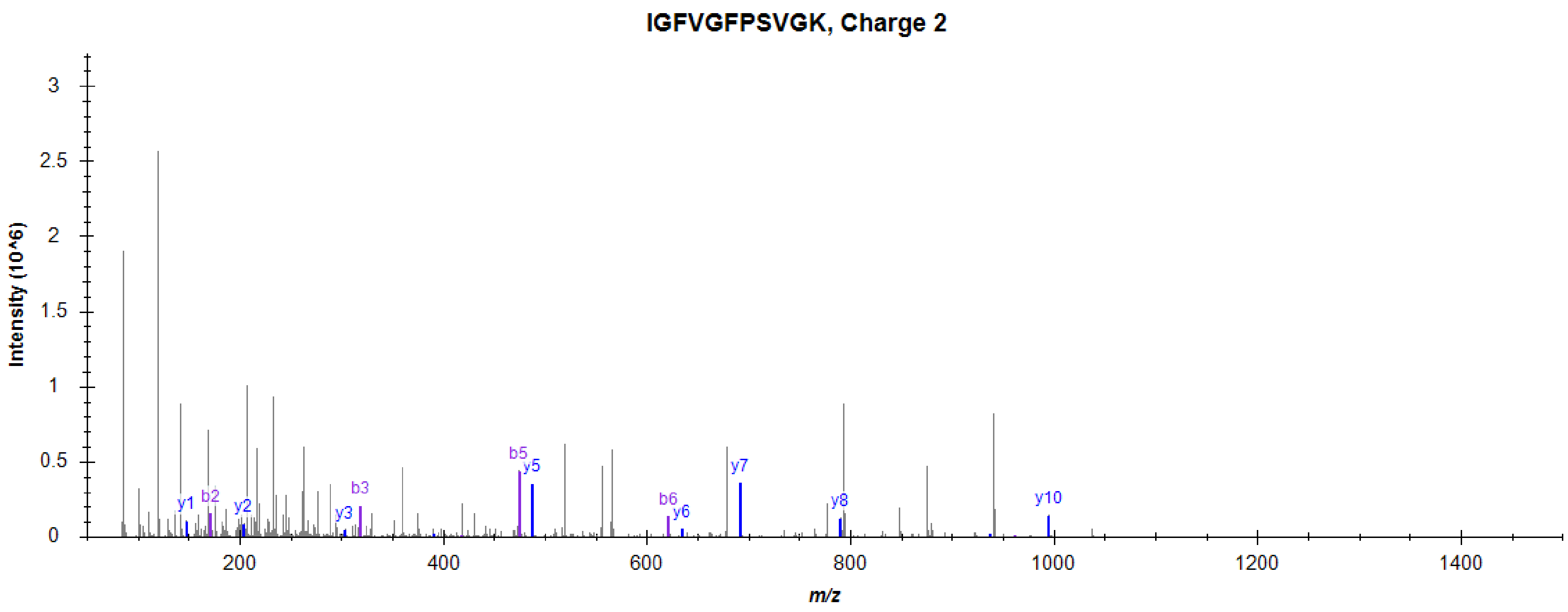
IGFVGFPSVGK, Charge 2
Intensity (10^6)
m/z
y1
b2
y2
y3
b3
b5
y5
b6
y6
y7
y8
y10

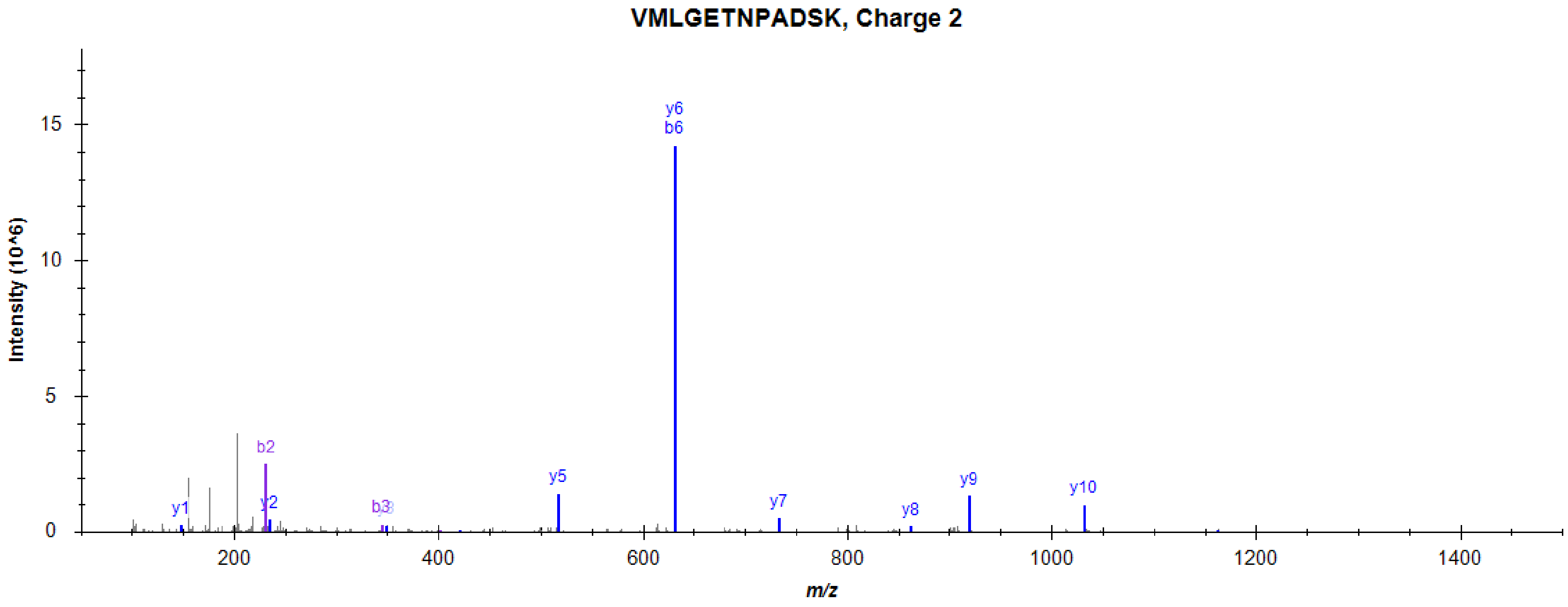
VMLGETNPADSK, Charge 2
Intensity (10^6)
m/z
0
5
10
15
200
400
600
800
1000
1200
1400
y6
b6
b2
y1
y2
b3
y5
y7
y8
y9
y10

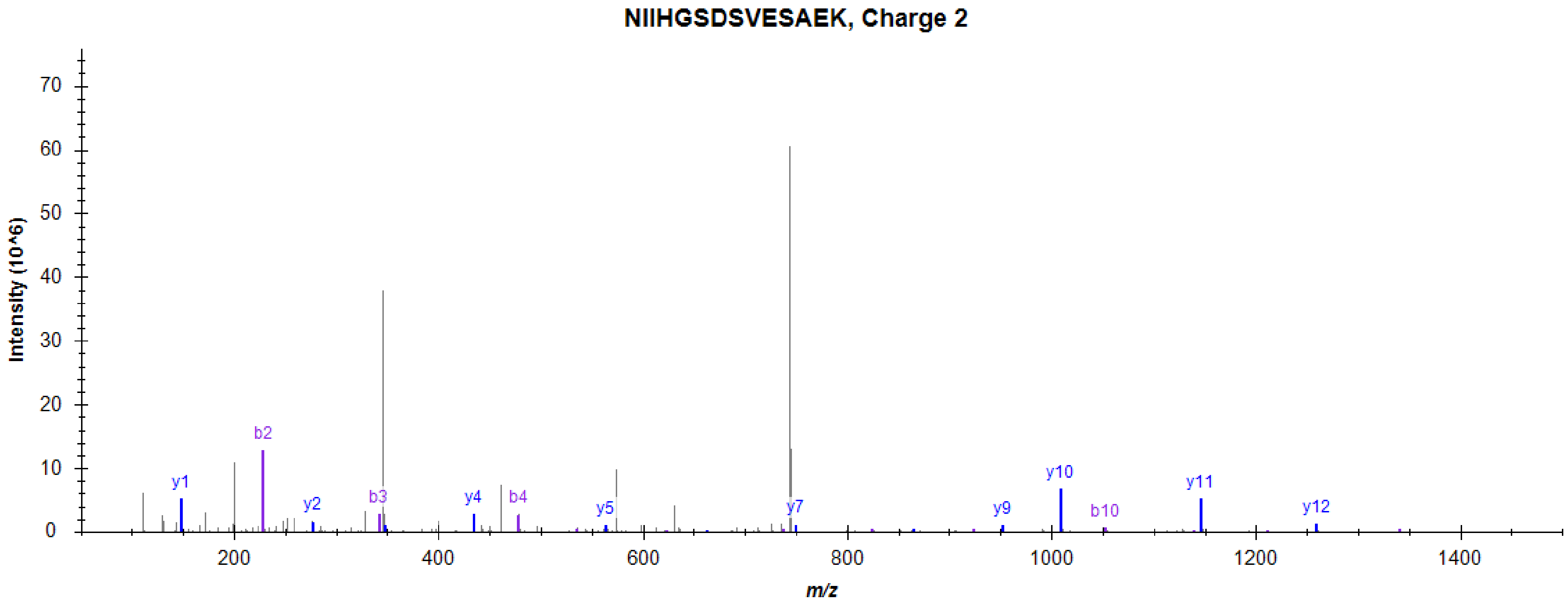

NIIHGSDSVESAEK, Charge 2
Intensity (10^6)
m/z
0
10
20
30
40
50
60
70
200
400
600
800
1000
1200
1400
y1
b2
y2
b3
y4
b4
y5
y7
y9
y10
b10
y11
y12

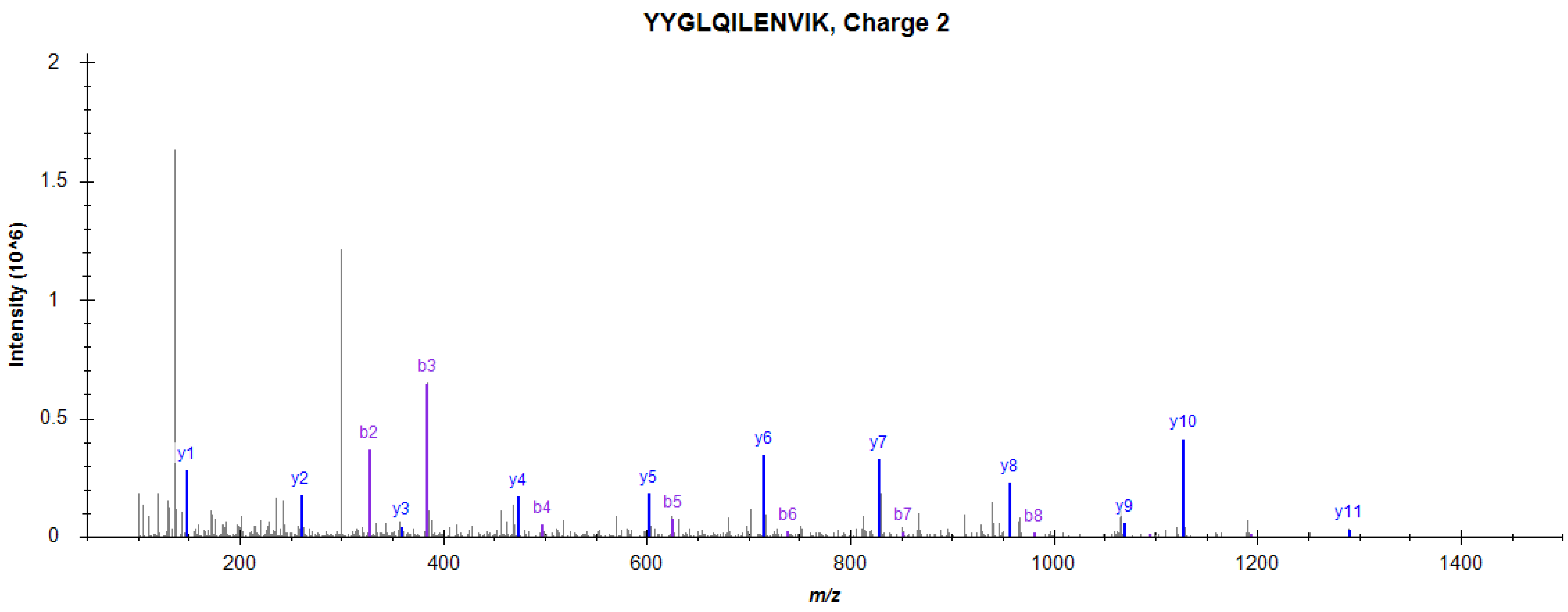
YYGLQILENVIK, Charge 2
Intensity (10^6)
2
1.5
1
0.5
0
y1
y2
b2
y3
b3
y4
b4
y5
b5
y6
b6
y7
b7
y8
b8
y9
y10
y11
200
400
600
800
1000
1200
1400
m/z

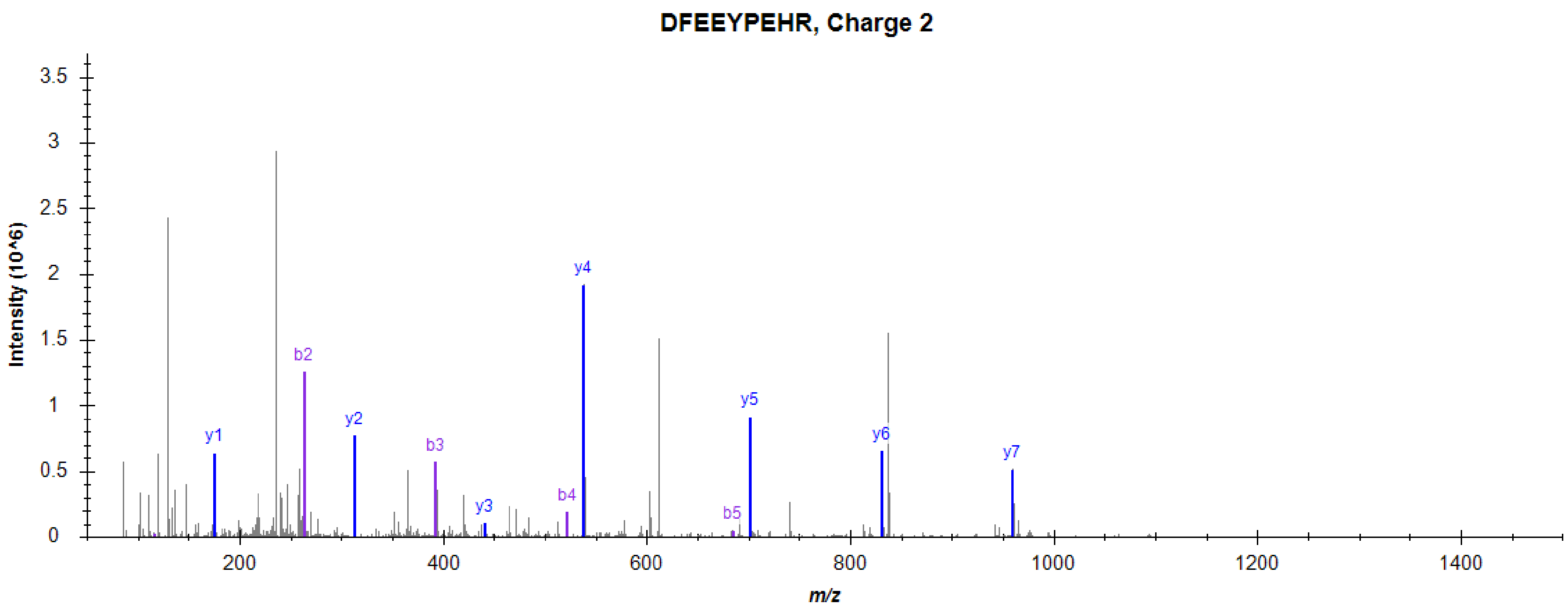

DFEEYPEHR, Charge 2
Intensity (10^6)
3.5
3
2.5
2
1.5
1
0.5
0
y1
b2
y2
b3
y3
b4
y4
b5
y5
y6
y7
200
400
600
800
1000
1200
1400
m/z

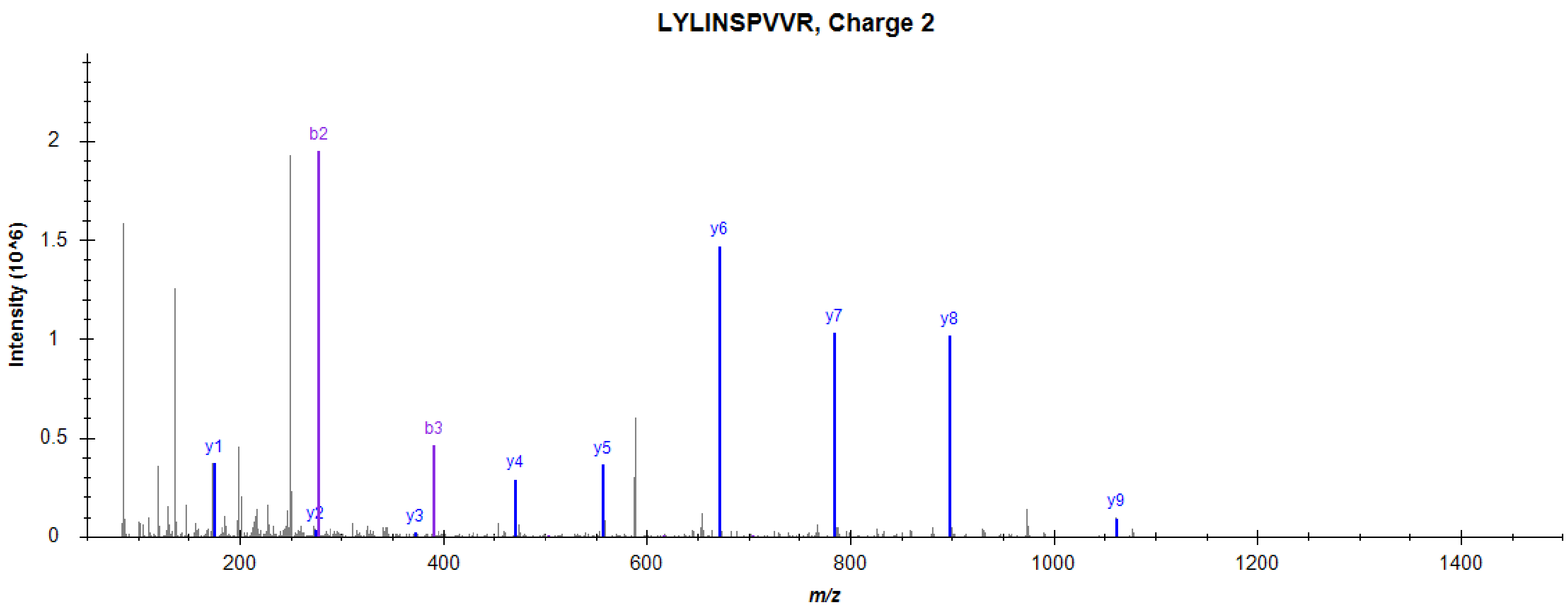
LYLINSPVVR, Charge 2
Intensity (10^6)
m/z
b2
b3
y1
y2
y3
y4
y5
y6
y7
y8
y9
0
0.5
1
1.5
2
200
400
600
800
1000
1200
1400

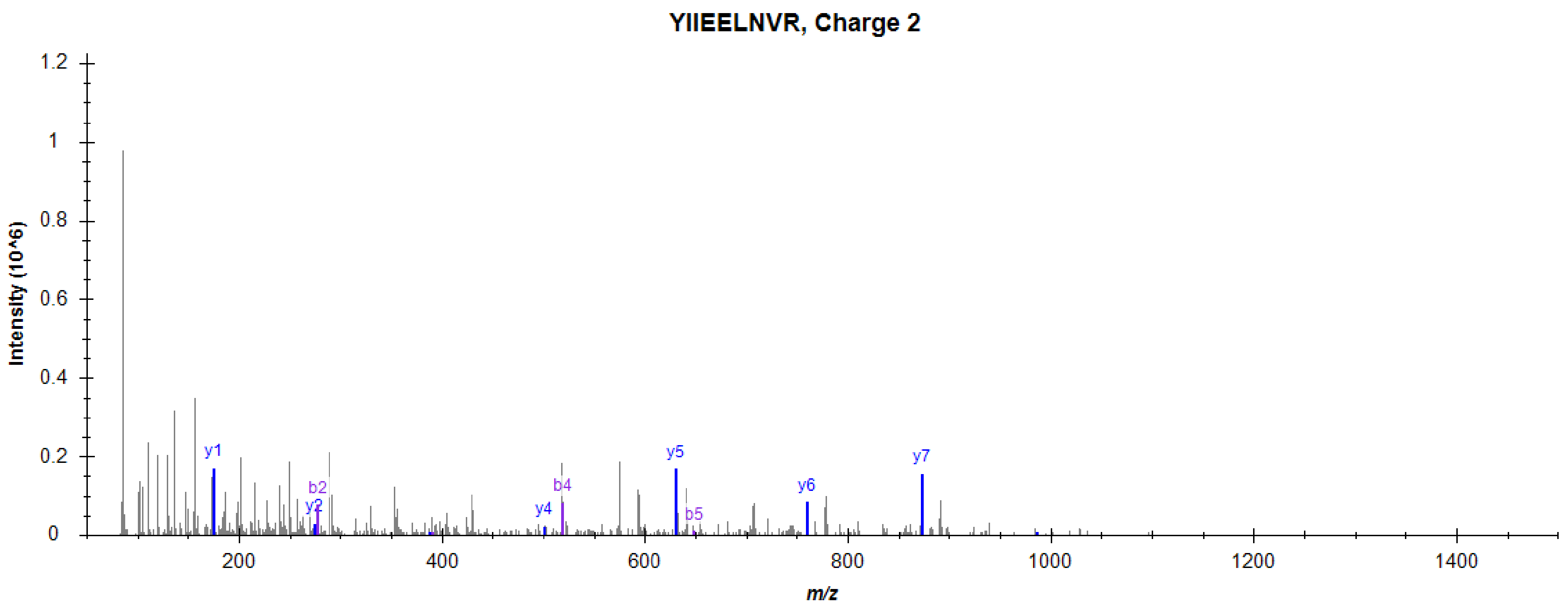
YIIEELNVR, Charge 2
Intensity (10^6)
1.2
1
0.8
0.6
0.4
0.2
0
y1
b2
y2
y4
b4
y5
b5
y6
y7
200
400
600
800
1000
1200
1400
m/z

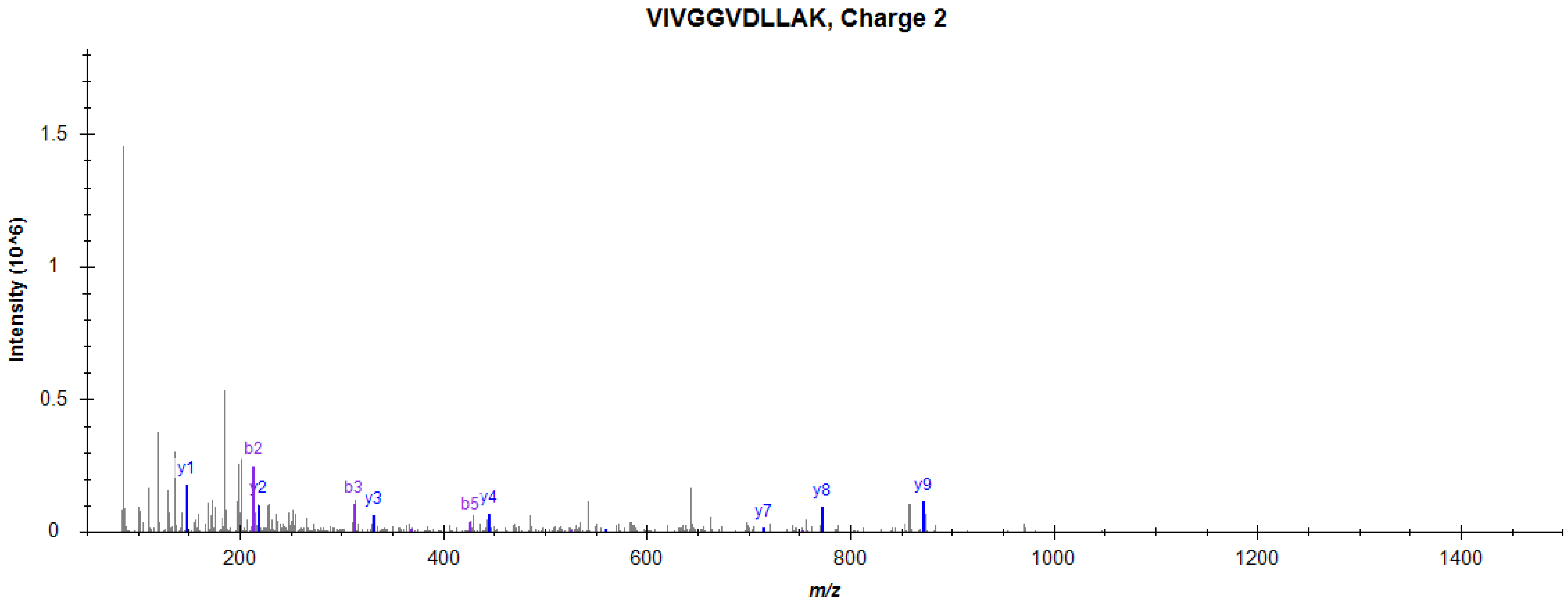

VIVGGVDLLAK, Charge 2
Intensity (10^6)
1.5
1
0.5
0
y1
b2
y2
b3
y3
b5
y4
y7
y8
y9
200
400
600
800
1000
1200
1400
m/z

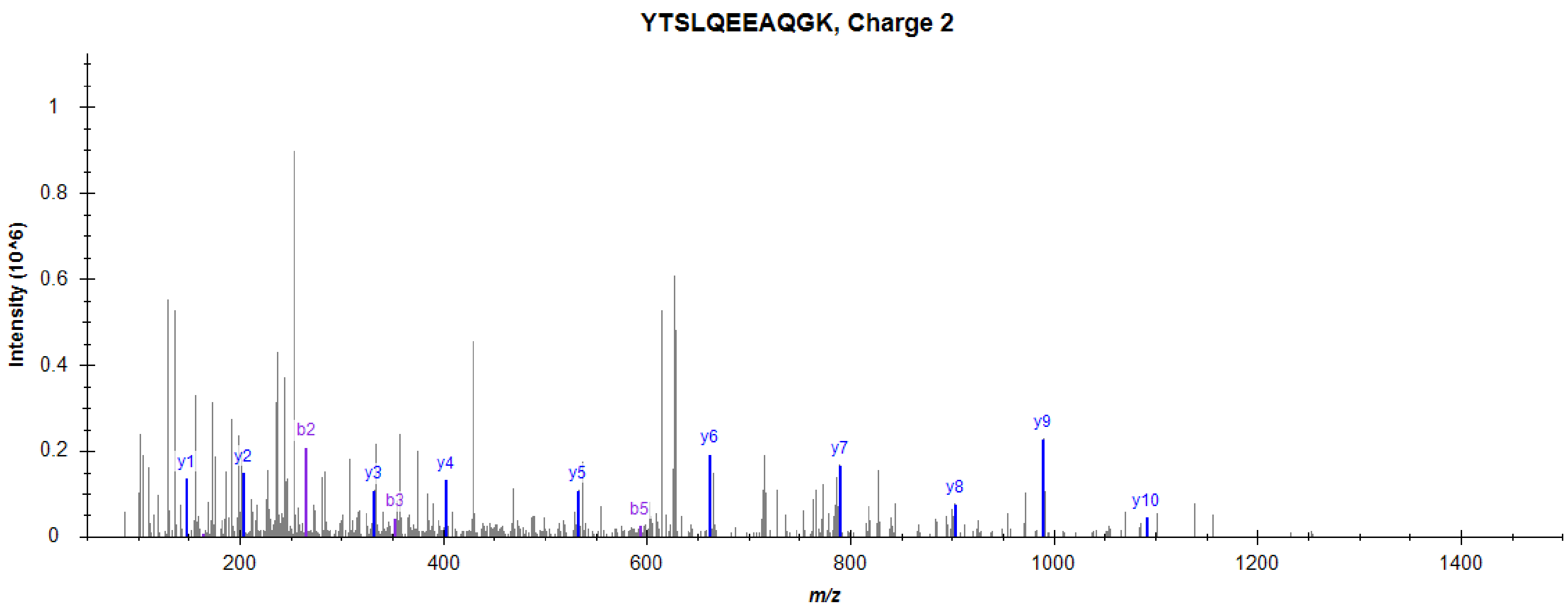

YTSLQEEAQGK, Charge 2
Intensity (10^6)
1
0.8
0.6
0.4
0.2
0
y1
y2
b2
y3
b3
y4
y5
b5
y6
y7
y8
y9
y10
200
400
600
800
1000
1200
1400
m/z

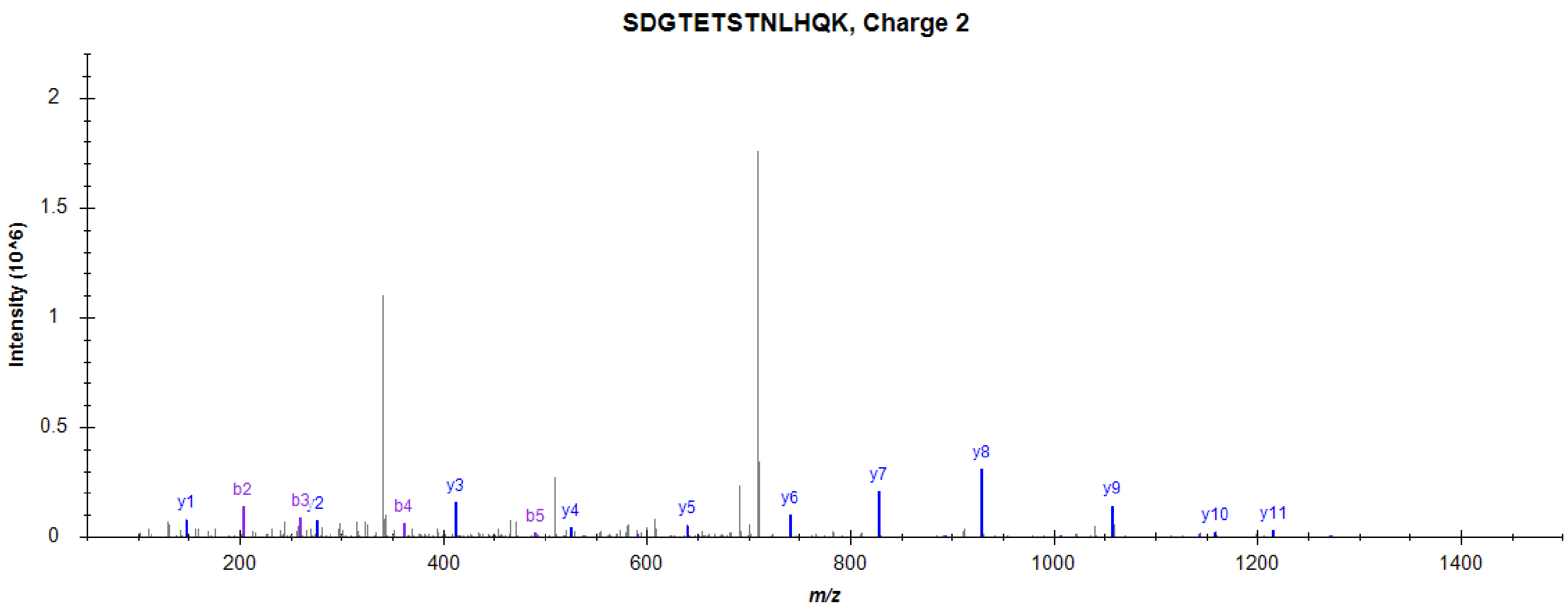
SDGTETSTNLHQK, Charge 2
Intensity (10^6)
m/z
0
0.5
1
1.5
2
200
400
600
800
1000
1200
1400
y1
b2
b3
y2
b4
y3
b5
y4
y5
y6
y7
y8
y9
y10
y11

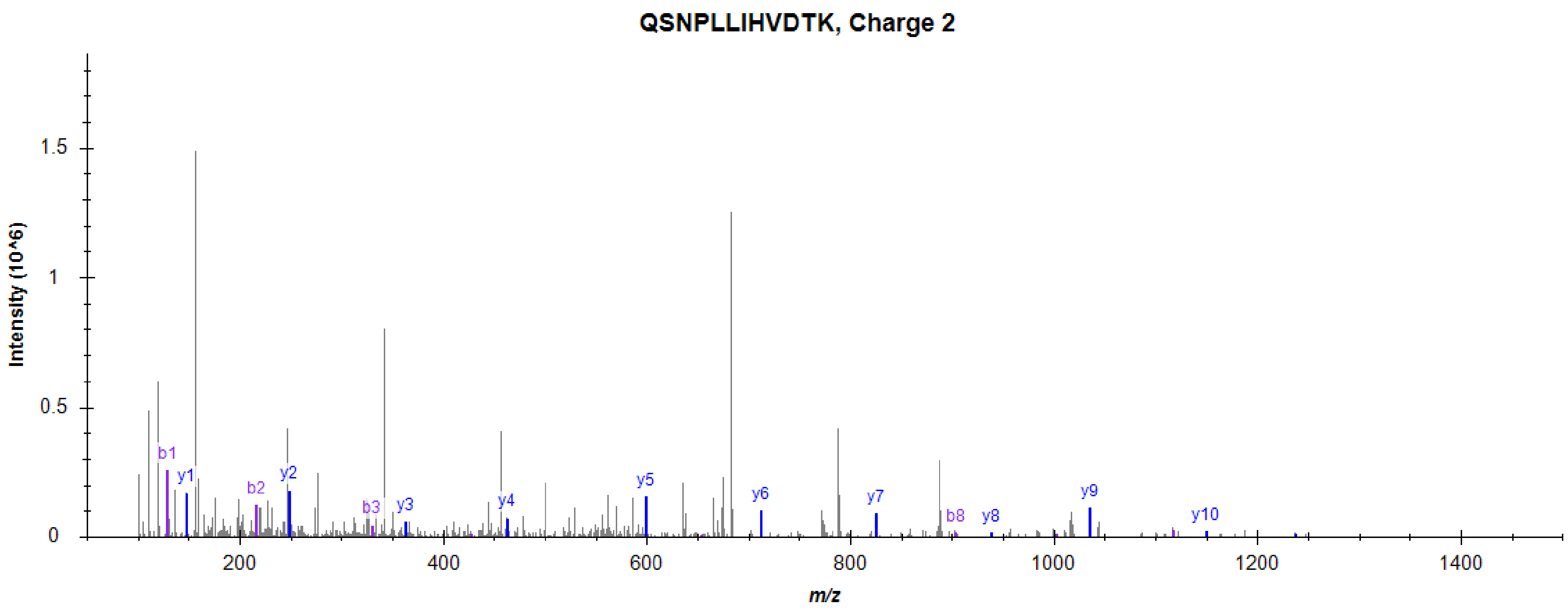
QSNPLLIHVDTK, Charge 2
Intensity (10^6)
m/z
0
0.5
1
1.5
200
400
600
800
1000
1200
1400
b1
y1
b2
y2
b3
y3
y4
y5
y6
y7
b8
y8
y9
y10

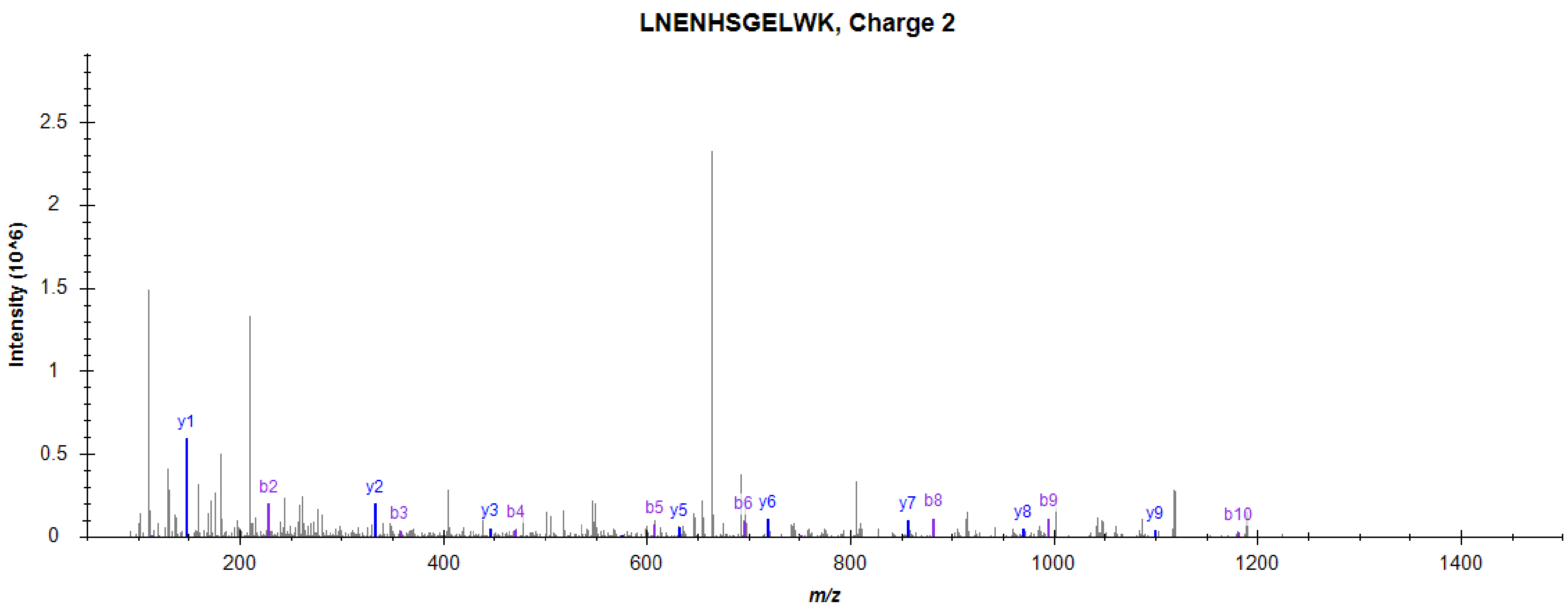

LNENHSGELWK, Charge 2
Intensity (10^6)
m/z
y1
b2
y2
b3
y3
b4
b5
y5
b6
y6
y7
b8
y8
b9
y9
b10

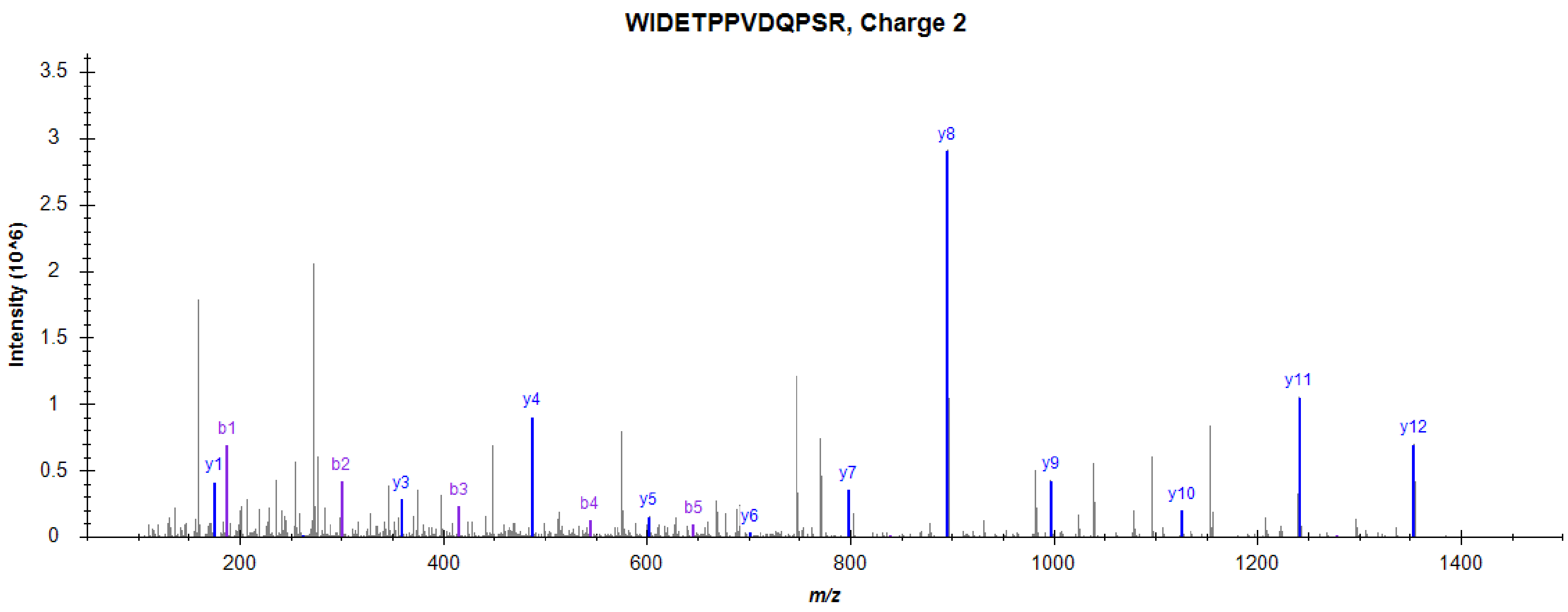
WIDETPPVDQPSR, Charge 2
Intensity (10^6)
m/z
y1
b1
b2
y3
b3
y4
b4
y5
b5
y6
y7
y8
y9
y10
y11
y12

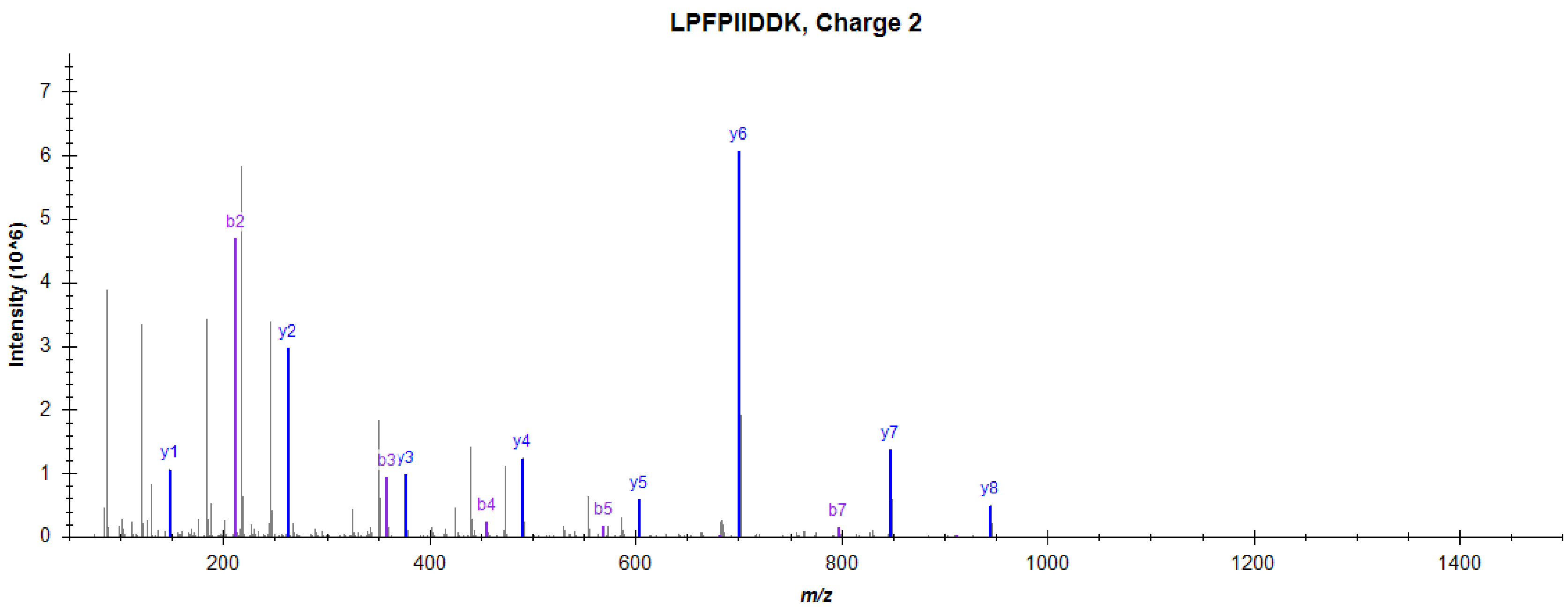
LPFPIIDDK, Charge 2
Intensity (10^6)
m/z
b2
y1
y2
b3
y3
b4
y4
b5
y5
y6
b7
y7
y8

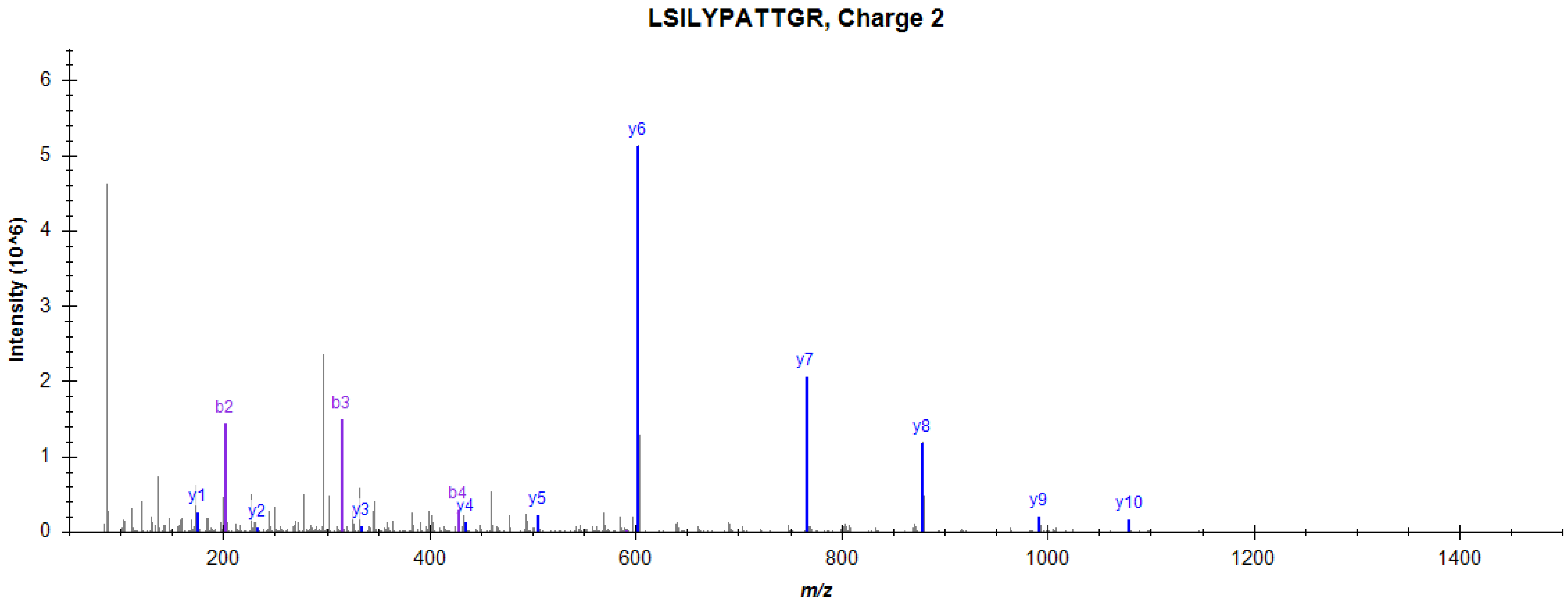
LSILYPATTGR, Charge 2
Intensity (10^6)
m/z
0
1
2
3
4
5
6
200
400
600
800
1000
1200
1400
y1
b2
y2
b3
y3
b4
y4
y5
y6
y7
y8
y9
y10

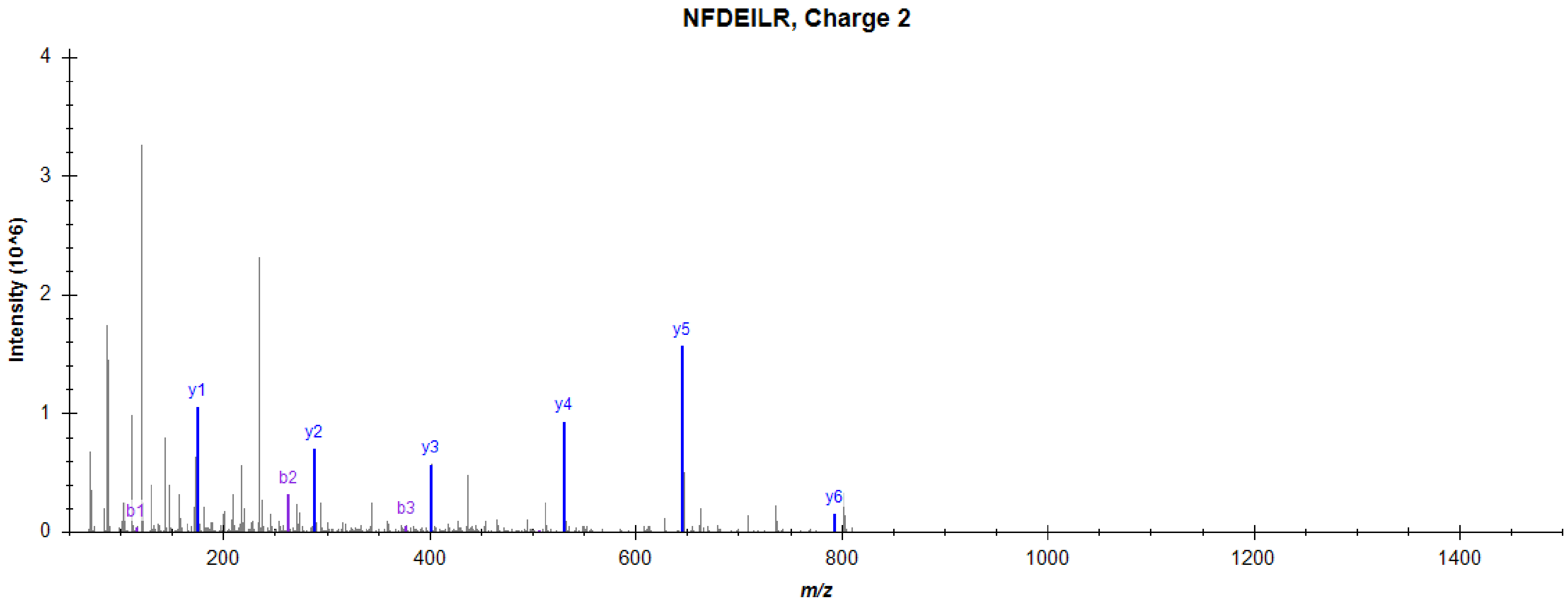

NFDEILR, Charge 2
Intensity (10^6)
m/z
0
1
2
3
4
200
400
600
800
1000
1200
1400
y1
y2
y3
y4
y5
y6
b1
b2
b3

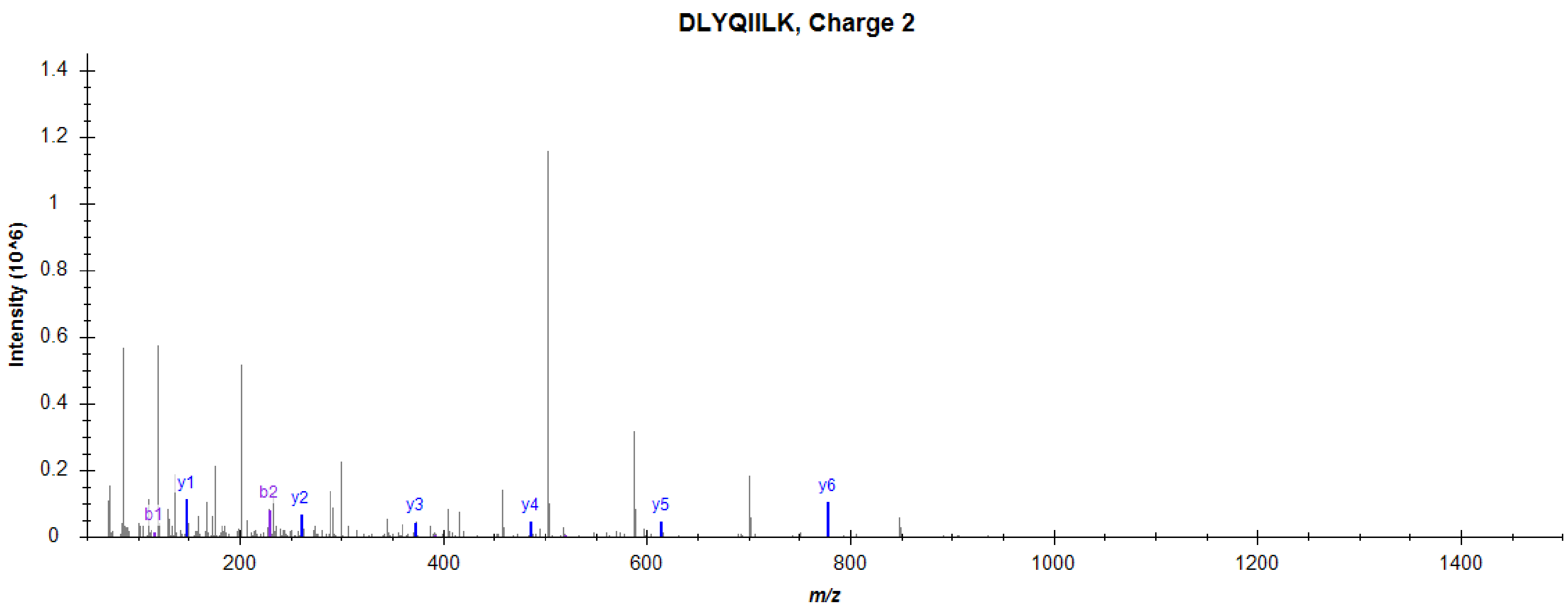
DLYQIILK, Charge 2
Intensity (10^6)
1.4
1.2
1
0.8
0.6
0.4
0.2
0
b1
y1
b2
y2
y3
y4
y5
y6
200
400
600
800
1000
1200
1400
m/z
