# Supplementary material for: Comparative Proteomic Profiling: Cellular Metabolisms Are Mainly Affected in Senecavirus A-Inoculated Cells at an Early Stage of Infection
Source: Viruses. 2021 May 31;13(6):1036. doi: 10.3390/v13061036 (PMC8226903; doi:10.3390/v13061036)

Skyline analyses of candidate peptides

AVFVDLEPTVIDEIR

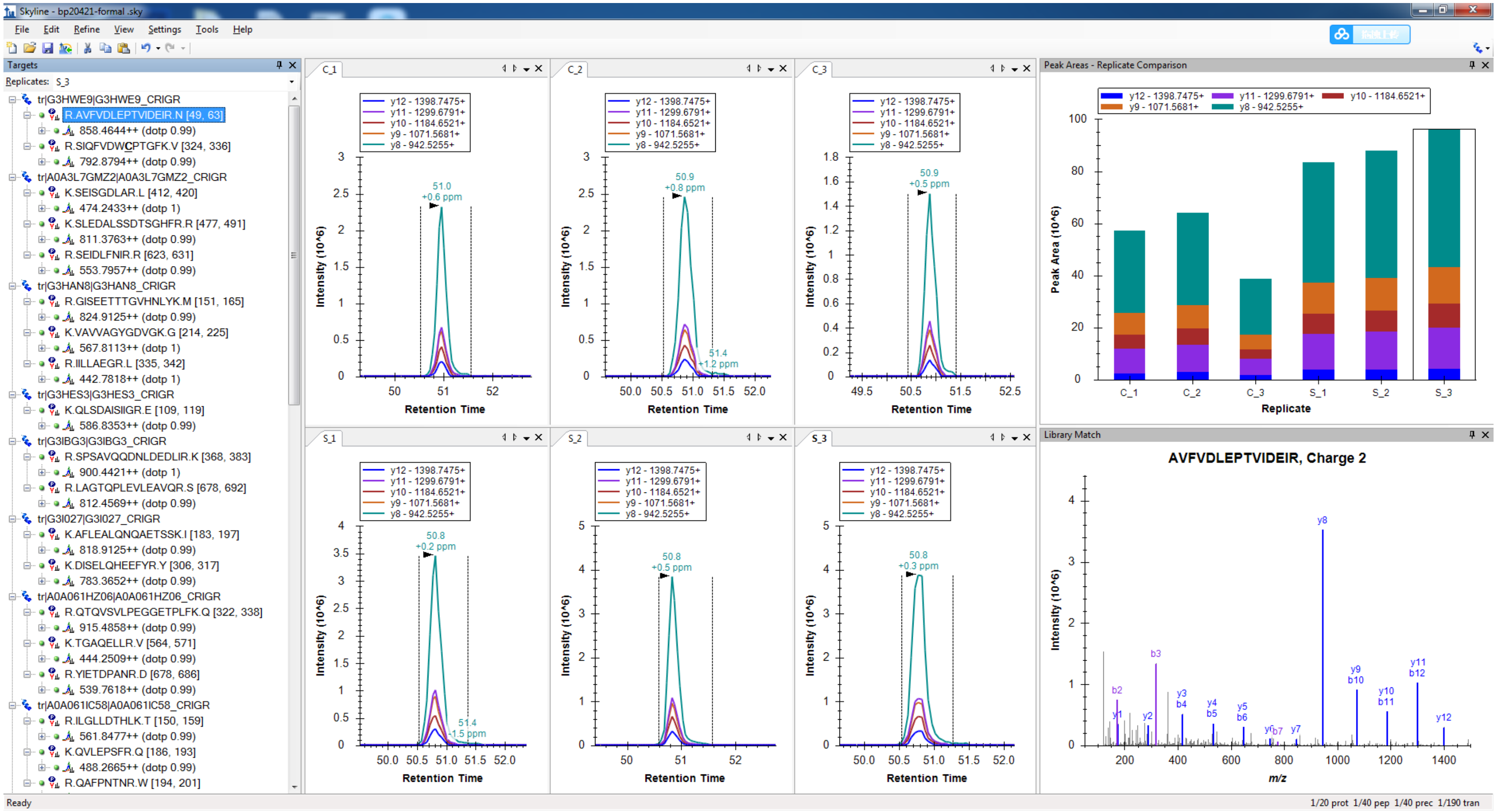

SIQFVDWCPTGFK

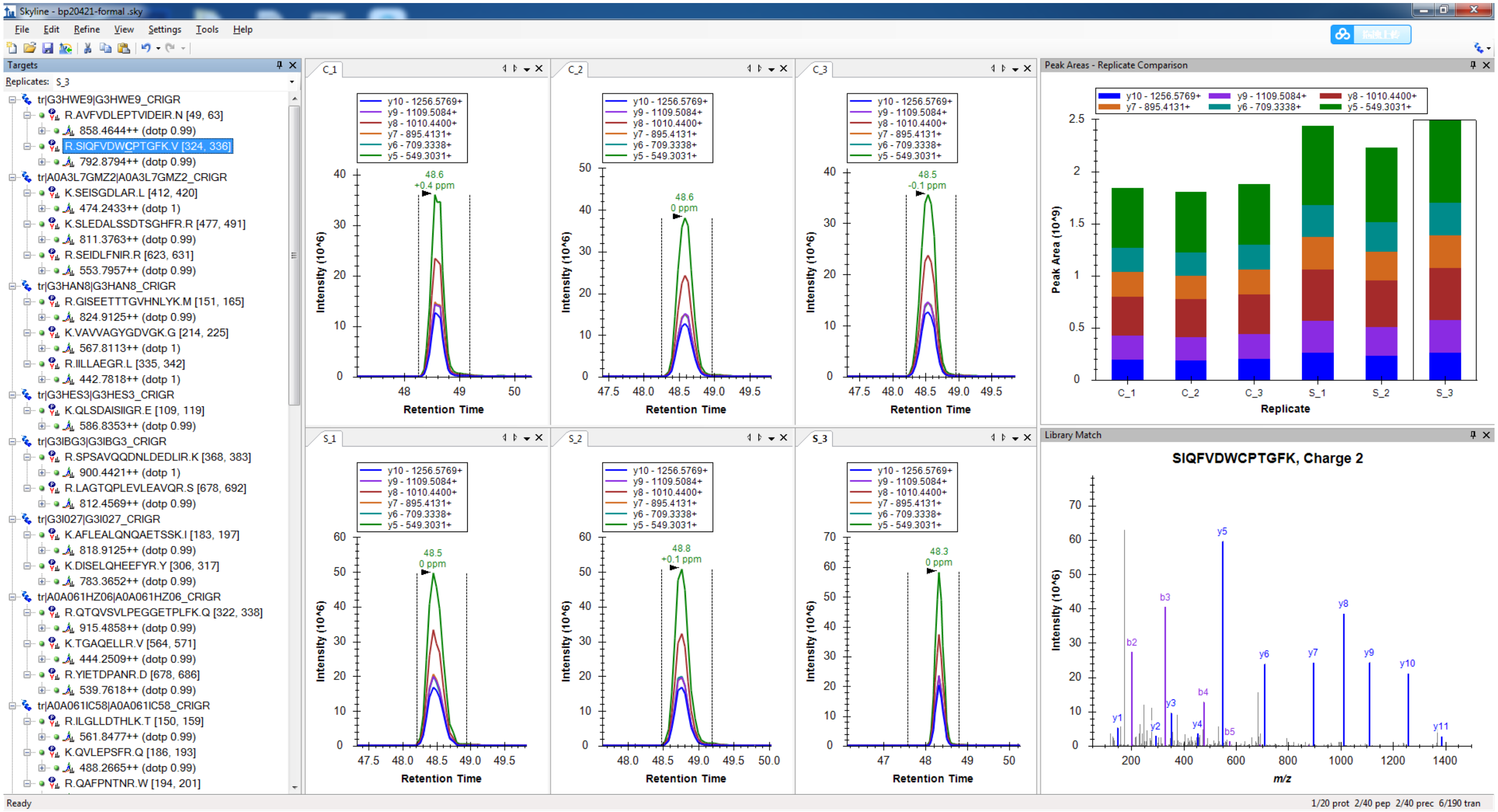

SEISGDLAR

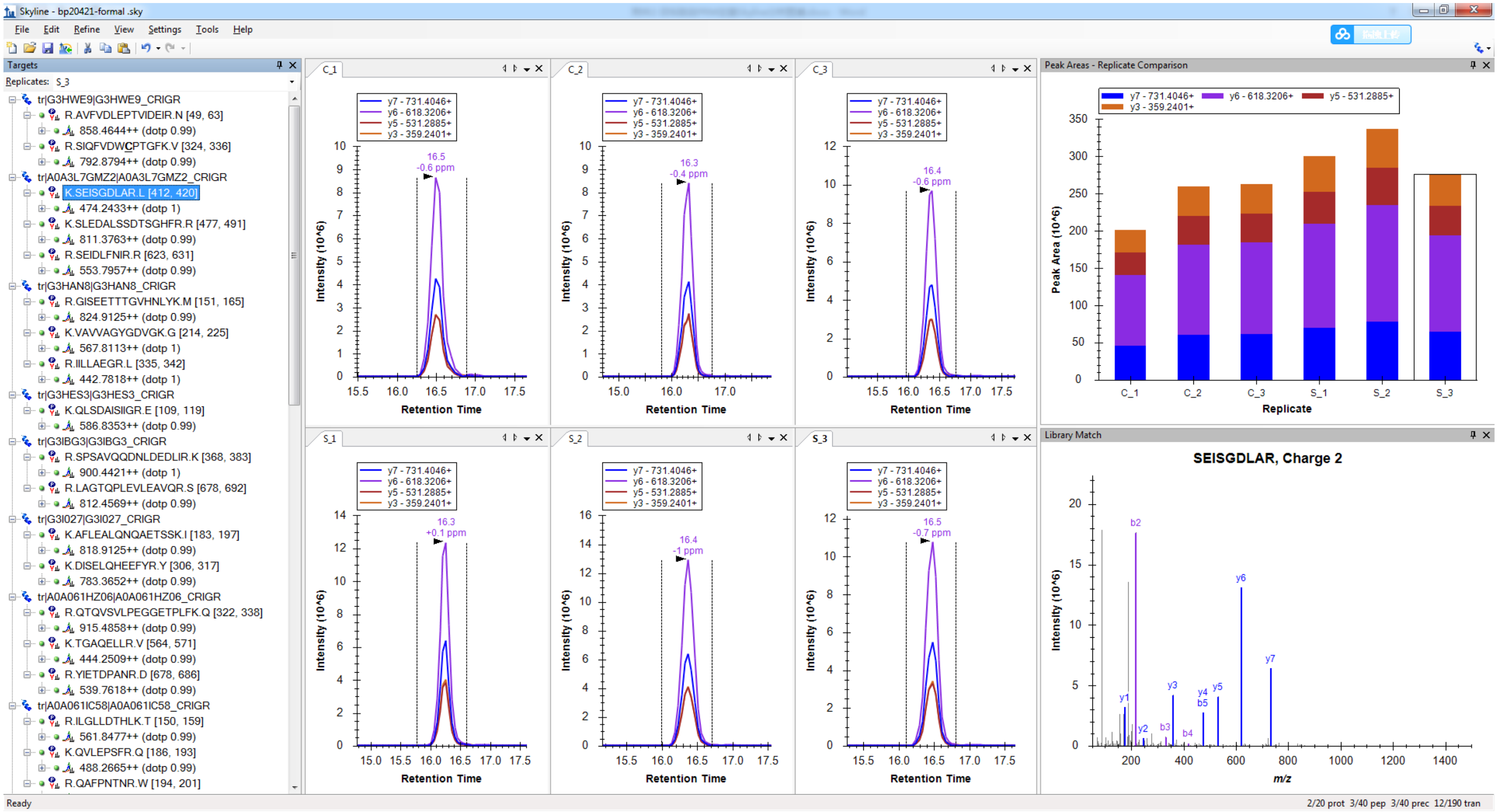
Skyline - bp20421-formal .sky
File Edit Refine View Settings Tools Help
Targets
Replicates: S_3
tr|G3HWE9|G3HWE9_CRIGR
R.AVFVDLEPTVIDEIR.N [49, 63]
858.4644++ (dotp 0.99)
R.SIQFVDWCPTGFK.V [324, 336]
792.8794++ (dotp 0.99)
tr|A0A3L7GMZ2|A0A3L7GMZ2_CRIGR
K.SEISGDLAR.L [412, 420]
474.2433++ (dotp 1)
K.SLEDALSSDTSGHFR.R [477, 491]
811.3763++ (dotp 0.99)
R.SEIDLFNIR.R [623, 631]
553.7957++ (dotp 0.99)
tr|G3HAN8|G3HAN8_CRIGR
R.GISEETTGVHNLYK.M [151, 165]
824.9125++ (dotp 0.99)
K.VAVVAGYGDVGK.G [214, 225]
567.8113++ (dotp 1)
R.IILLAEGR.L [335, 342]
442.7818++ (dotp 1)
tr|G3HES3|G3HES3_CRIGR
K.QLSDAISIIGR.E [109, 119]
586.8353++ (dotp 0.99)
tr|G3IBG3|G3IBG3_CRIGR
R.SPSAVQQDNLDEDLIR.K [368, 383]
900.4421++ (dotp 1)
R.LAGTQPLEVLEAVQR.S [678, 692]
812.4569++ (dotp 0.99)
tr|G3I027|G3I027_CRIGR
K.AFLEALQNQAETSSK.I [183, 197]
818.9125++ (dotp 0.99)
K.DISELQHEEFYR.Y [306, 317]
783.3652++ (dotp 0.99)
tr|A0A061HZ06|A0A061HZ06_CRIGR
R.QTQVSVLPEGGETPLFK.Q [322, 338]
915.4858++ (dotp 0.99)
K.TGAQELLR.V [564, 571]
444.2509++ (dotp 0.99)
R.YIETDPANR.D [678, 686]
539.7618++ (dotp 0.99)
tr|A0A061IC58|A0A061IC58_CRIGR
R.ILGLLDTHLK.T [150, 159]
561.8477++ (dotp 0.99)
K.QVLEPSFR.Q [186, 193]
488.2665++ (dotp 0.99)
R.QAFPNTNR.W [194, 201]
C_1
C_2
C_3
S_1
S_2
S_3
y7 - 731.4046+
y6 - 618.3206+
y5 - 531.2885+
y3 - 359.2401+
Intensity (10^6)
Retention Time
16.5
-0.6 ppm
16.3
-0.4 ppm
16.4
-0.6 ppm
16.3
+0.1 ppm
16.4
-1 ppm
16.5
-0.7 ppm
Peak Areas - Replicate Comparison
Peak Area (10^6)
Replicate
Library Match
SEISGDLAR, Charge 2
Intensity (10^6)
m/z
b2
y6
y7
y3
y4
b5
y5
y1
y2
b3
b4
Ready
2/20 prot 3/40 pep 3/40 prec 12/190 tran

SLEDALSSDTSGHFR

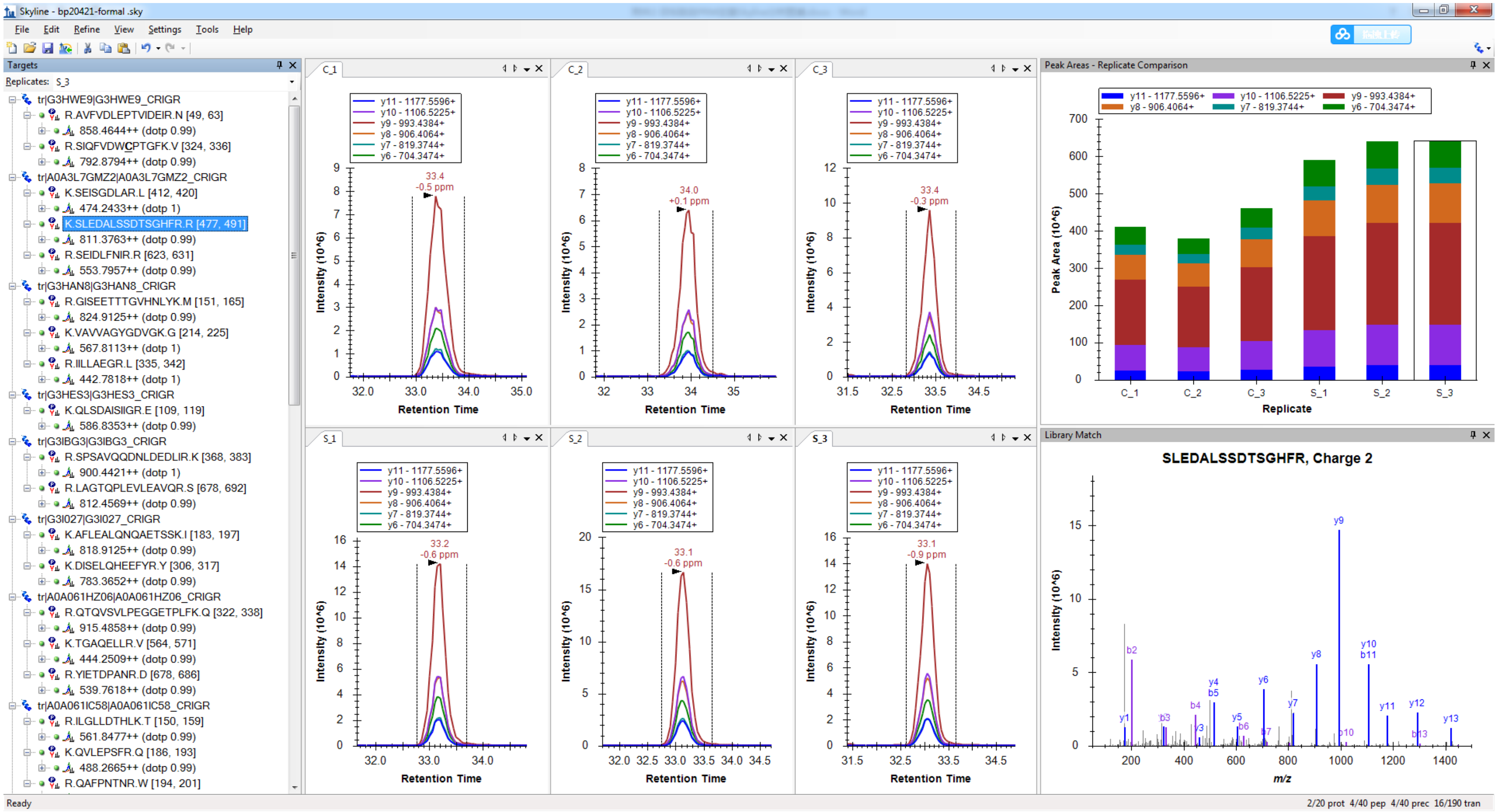

SEIDLFNIR

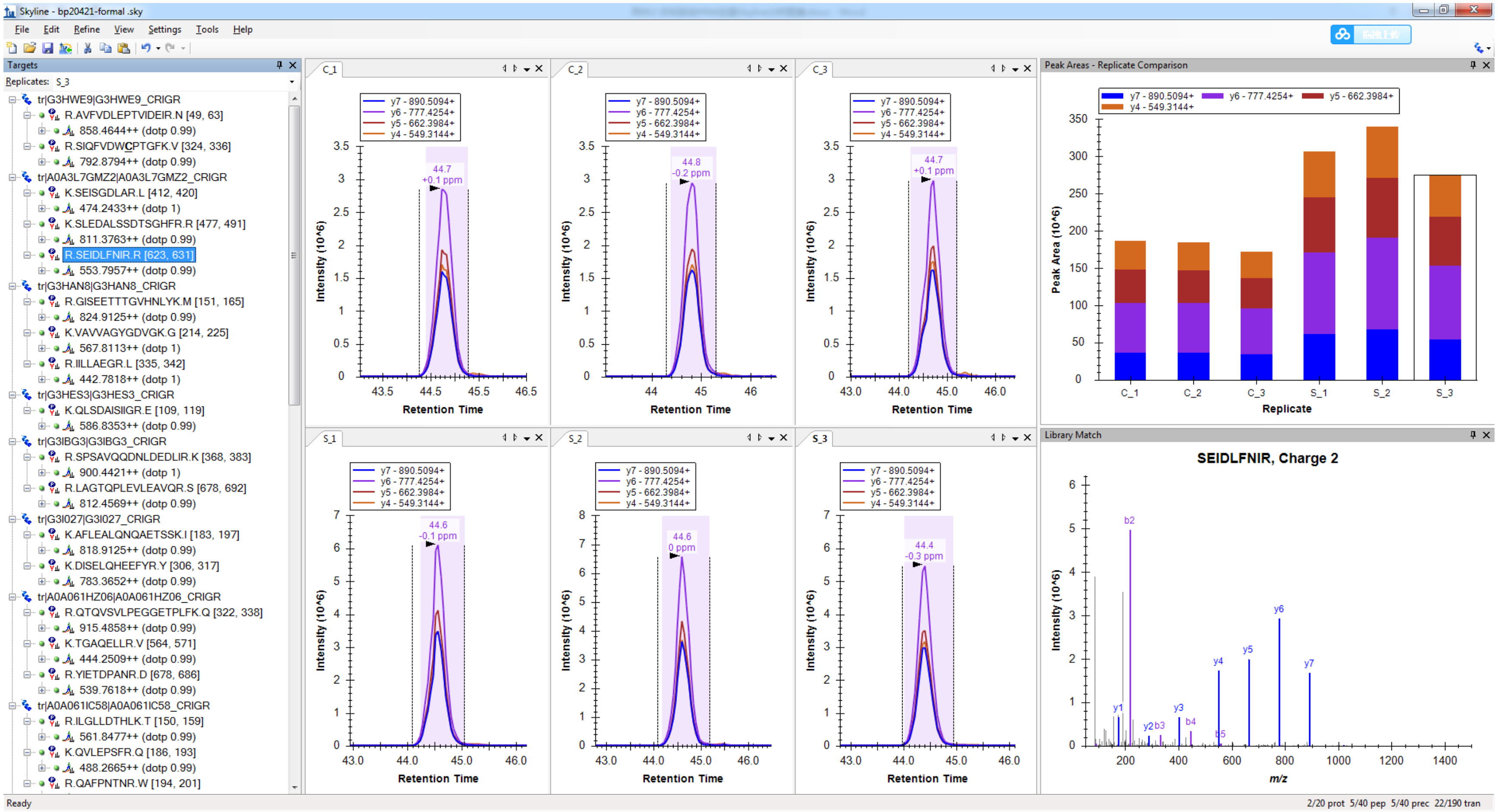
Skyline - bp20421-formal .sky
File Edit Refine View Settings Tools Help
Targets
Replicates: S_3
tr|G3HWE9|G3HWE9_CRIGR
R.AVFVDLEPTVIDEIR.N [49, 63]
858.4644++ (dotp 0.99)
R.SIQFVDWCPTGFK.V [324, 336]
792.8794++ (dotp 0.99)
tr|A0A3L7GMZ2|A0A3L7GMZ2_CRIGR
K.SEISGDLAR.L [412, 420]
474.2433++ (dotp 1)
K.SLEDALSSDTSGHFR.R [477, 491]
811.3763++ (dotp 0.99)
R.SEIDLFNIR.R [623, 631]
553.7957++ (dotp 0.99)
tr|G3HAN8|G3HAN8_CRIGR
R.GISEETTGVHNLYK.M [151, 165]
824.9125++ (dotp 0.99)
K.VAVVAGYGDVGK.G [214, 225]
567.8113++ (dotp 1)
R.IILLAEGR.L [335, 342]
442.7818++ (dotp 1)
tr|G3HES3|G3HES3_CRIGR
K.QLSDAISIIGR.E [109, 119]
586.8353++ (dotp 0.99)
tr|G3IBG3|G3IBG3_CRIGR
R.SPSAVQQDNLDEDLIR.K [368, 383]
900.4421++ (dotp 1)
R.LAGTQPLEVLEAVQR.S [678, 692]
812.4569++ (dotp 0.99)
tr|G3I027|G3I027_CRIGR
K.AFLEALQNQAETSSK.I [183, 197]
818.9125++ (dotp 0.99)
K.DISELQHEEFYR.Y [306, 317]
783.3652++ (dotp 0.99)
tr|A0A061HZ06|A0A061HZ06_CRIGR
R.QTQVSVLPEGGETPLFK.Q [322, 338]
915.4858++ (dotp 0.99)
K.TGAQELLR.V [564, 571]
444.2509++ (dotp 0.99)
R.YIETDPANR.D [678, 686]
539.7618++ (dotp 0.99)
tr|A0A061IC58|A0A061IC58_CRIGR
R.ILGLLDTHLK.T [150, 159]
561.8477++ (dotp 0.99)
K.QVLEPSFR.Q [186, 193]
488.2665++ (dotp 0.99)
R.QAFPNTNR.W [194, 201]
C_1
C_2
C_3
S_1
S_2
S_3
y7 - 890.5094+
y6 - 777.4254+
y5 - 662.3984+
y4 - 549.3144+
44.7 +0.1 ppm
44.8 -0.2 ppm
44.7 +0.1 ppm
44.6 -0.1 ppm
44.6 0 ppm
44.4 -0.3 ppm
Intensity (10^6)
Retention Time
Peak Areas - Replicate Comparison
Peak Area (10^6)
Replicate
Library Match
SEIDLFNIR, Charge 2
b2
y1
y2
b3
y3
b4
b5
y4
y5
y6
y7
m/z
Ready
2/20 prot 5/40 pep 5/40 prec 22/190 tran

GISEETTTGVHNLYK

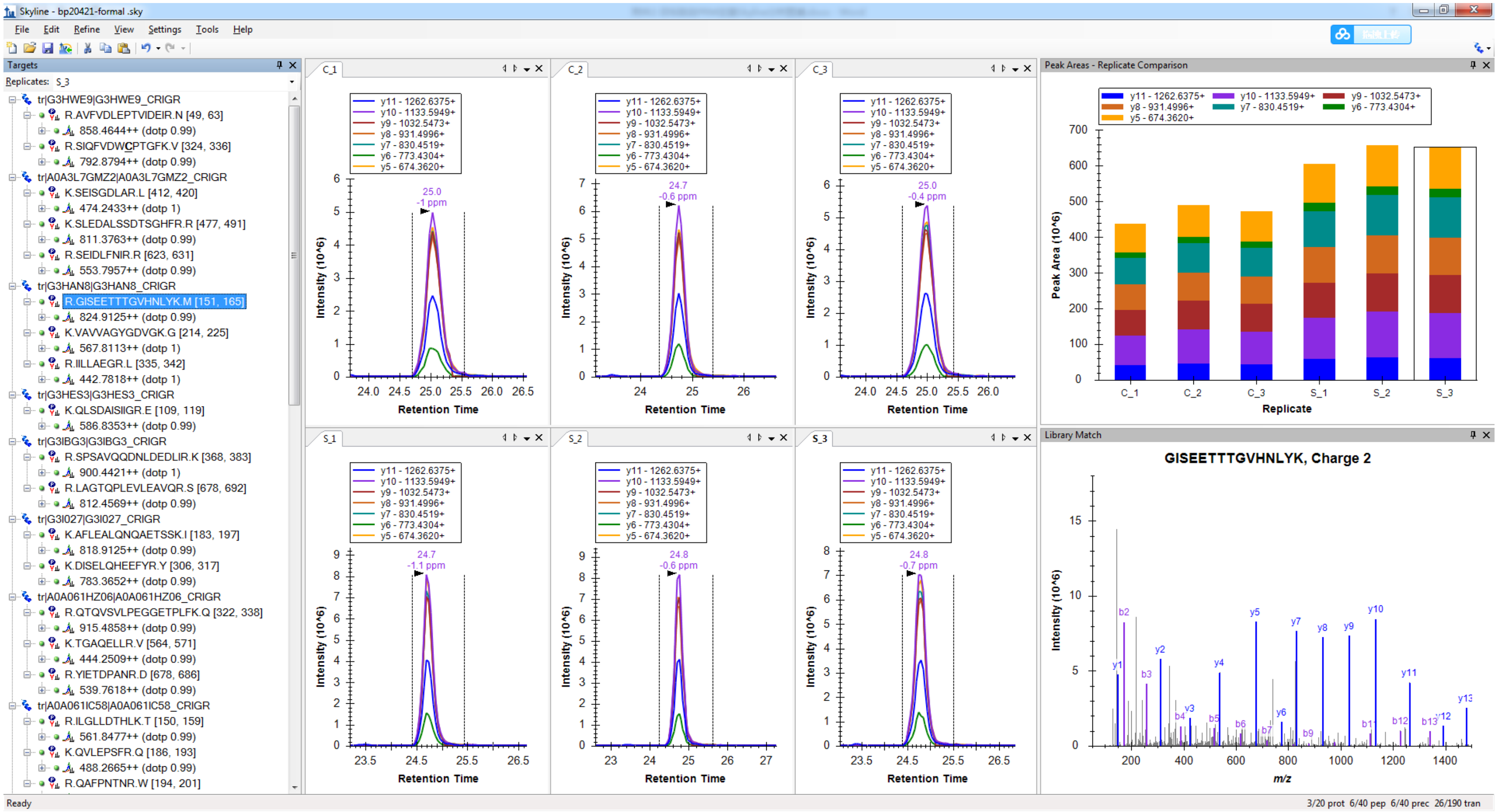

VAVVAGYGDVGK

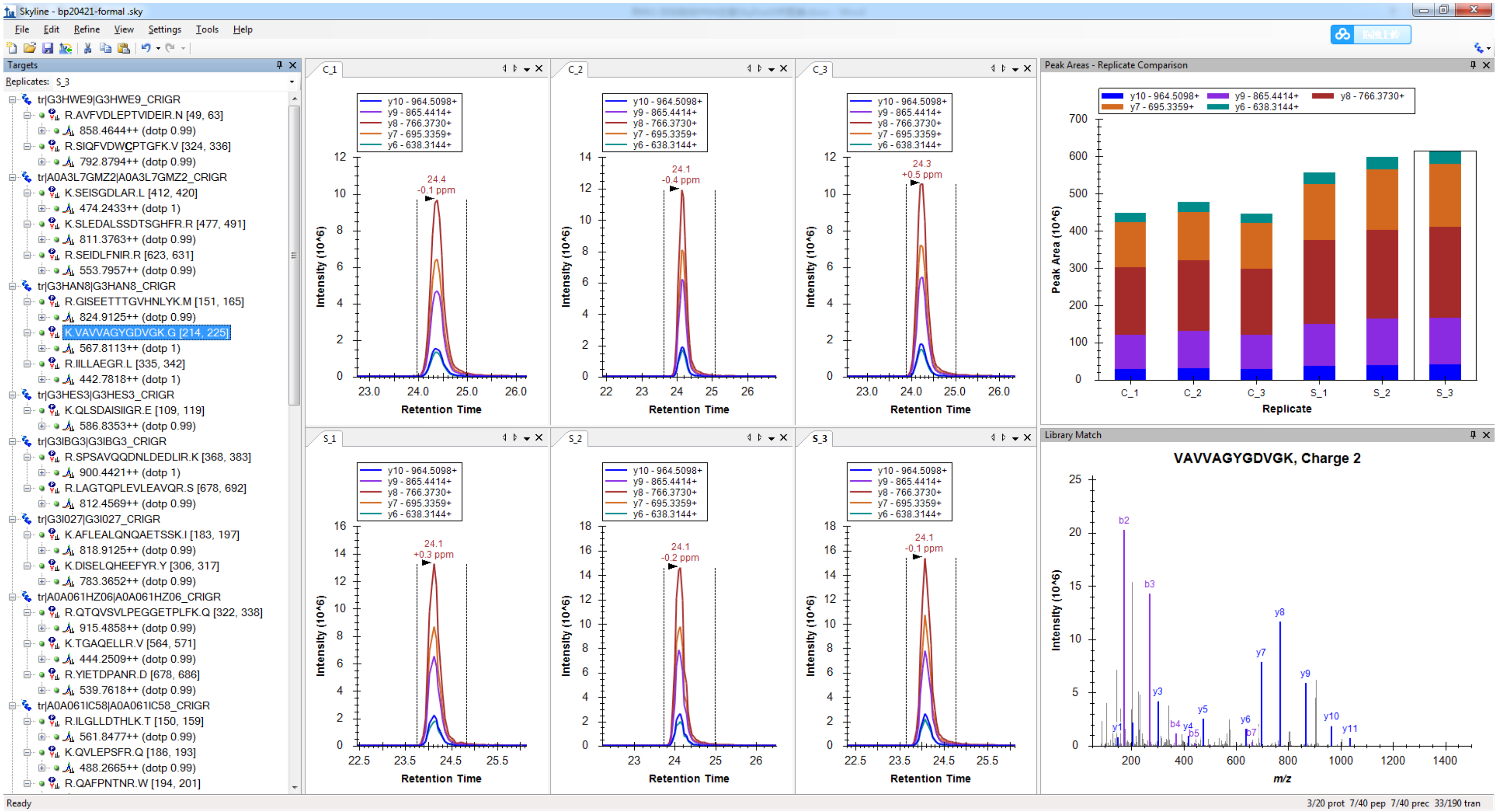

IILLAEGR

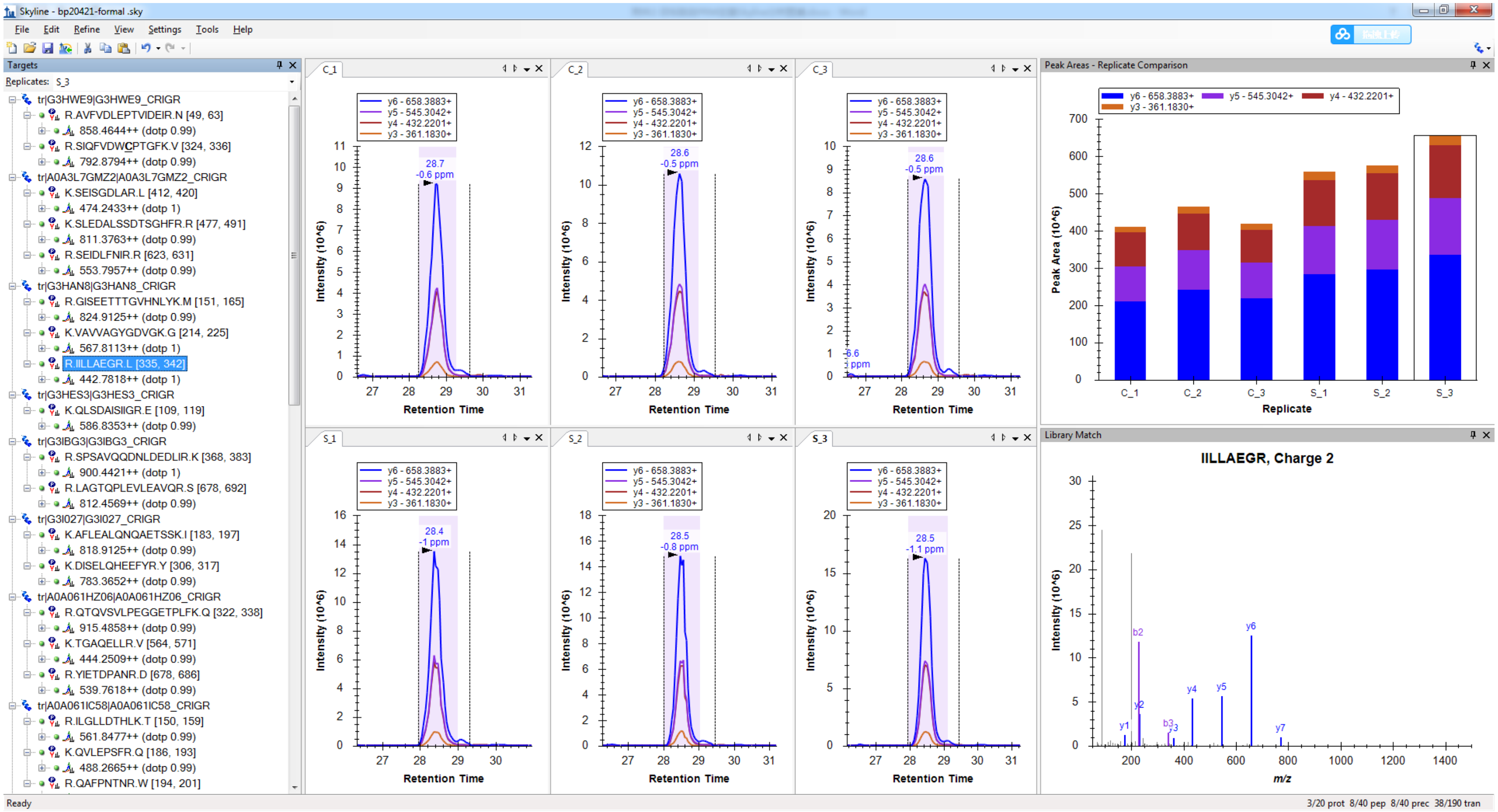

Skyline - bp20421-formal .sky
File Edit Refine View Settings Tools Help
Targets
Replicates: S_3
tr|G3HWE9|G3HWE9_CRIGR
R.AVFVDLEPTVIDEIR.N [49, 63]
858.4644++ (dotp 0.99)
R.SIQFVDWCPTGFK.V [324, 336]
792.8794++ (dotp 0.99)
tr|A0A3L7GMZ2|A0A3L7GMZ2_CRIGR
K.SEISGDLAR.L [412, 420]
474.2433++ (dotp 1)
K.SLEDALSSDTSGHFR.R [477, 491]
811.3763++ (dotp 0.99)
R.SEIDLFNIR.R [623, 631]
553.7957++ (dotp 0.99)
tr|G3HAN8|G3HAN8_CRIGR
R.GISEETTTGVHNLYK.M [151, 165]
824.9125++ (dotp 0.99)
K.VAVVAGYGDVGK.G [214, 225]
567.8113++ (dotp 1)
R.IILLAEGR.L [335, 342]
442.7818++ (dotp 1)
tr|G3HES3|G3HES3_CRIGR
K.QLSDAISIIGR.E [109, 119]
586.8353++ (dotp 0.99)
tr|G3IBG3|G3IBG3_CRIGR
R.SPSAVQQDNLDEDLIR.K [368, 383]
900.4421++ (dotp 1)
R.LAGTQPLEVLEAVQR.S [678, 692]
812.4569++ (dotp 0.99)
tr|G3I027|G3I027_CRIGR
K.AFLEALQNQAETSSK.I [183, 197]
818.9125++ (dotp 0.99)
K.DISELQHEEFYR.Y [306, 317]
783.3652++ (dotp 0.99)
tr|A0A061HZ06|A0A061HZ06_CRIGR
R.QTQVSVLPEGGETPLFK.Q [322, 338]
915.4858++ (dotp 0.99)
K.TGAQELLR.V [564, 571]
444.2509++ (dotp 0.99)
R.YIETDPANR.D [678, 686]
539.7618++ (dotp 0.99)
tr|A0A061IC58|A0A061IC58_CRIGR
R.ILGLLDTHLK.T [150, 159]
561.8477++ (dotp 0.99)
K.QVLEPSFR.Q [186, 193]
488.2665++ (dotp 0.99)
R.QAFPNTNR.W [194, 201]
C_1
C_2
C_3
S_1
S_2
S_3
y6 - 658.3883+
y5 - 545.3042+
y4 - 432.2201+
y3 - 361.1830+
28.7
-0.6 ppm
28.6
-0.5 ppm
28.6
-0.5 ppm
-6.6 ppm
28.4
-1 ppm
28.5
-0.8 ppm
28.5
-1.1 ppm
Intensity (10^6)
Retention Time
Peak Areas - Replicate Comparison
Peak Area (10^6)
Replicate
Library Match
IILLAEGR, Charge 2
y1
b2
y2
b3
y3
y4
y5
y6
y7
m/z
Ready
3/20 prot 8/40 pep 8/40 prec 38/190 tran

QLSDAISIIGR

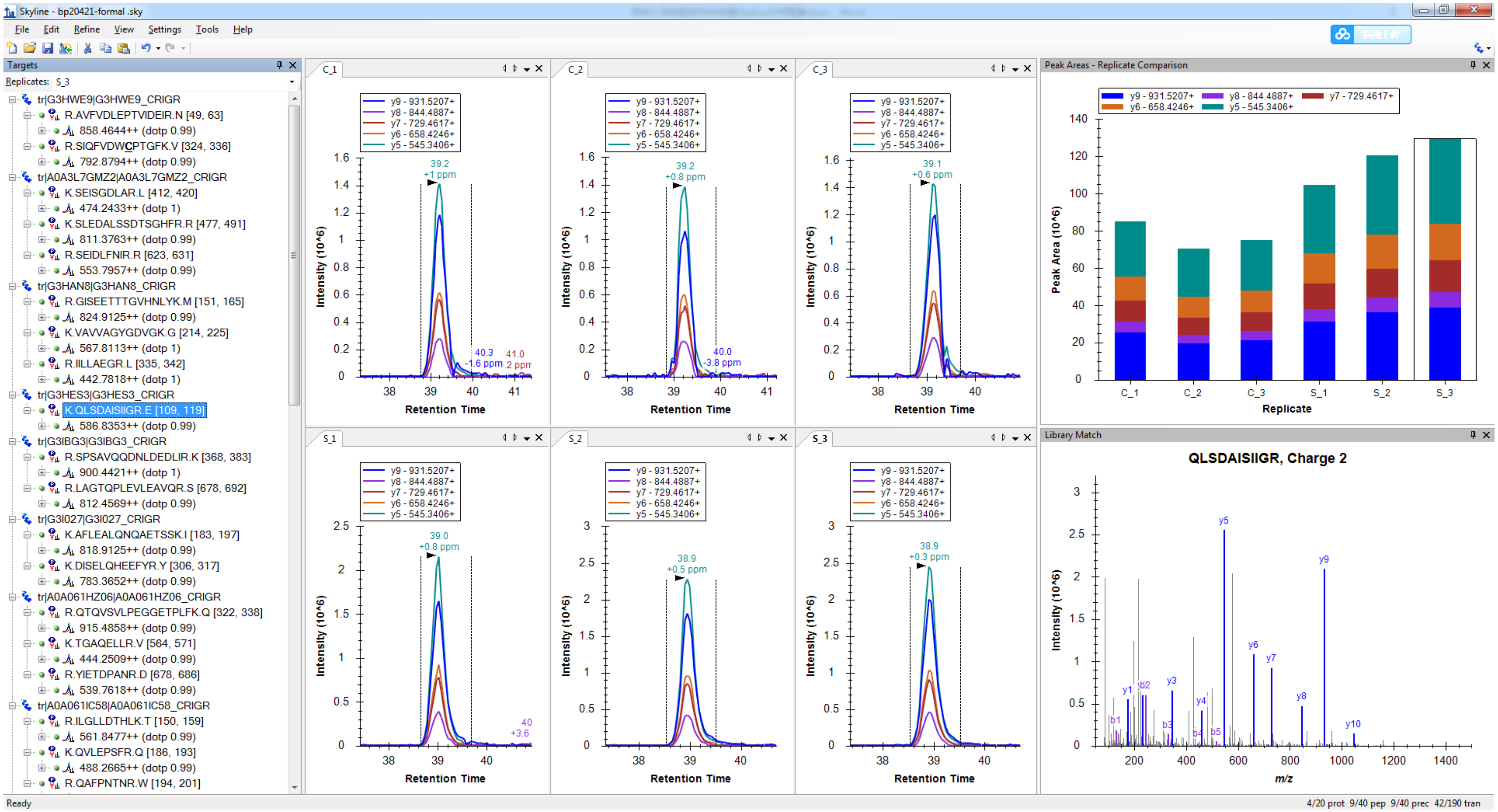

SPSAVQQDNLDEDLIR

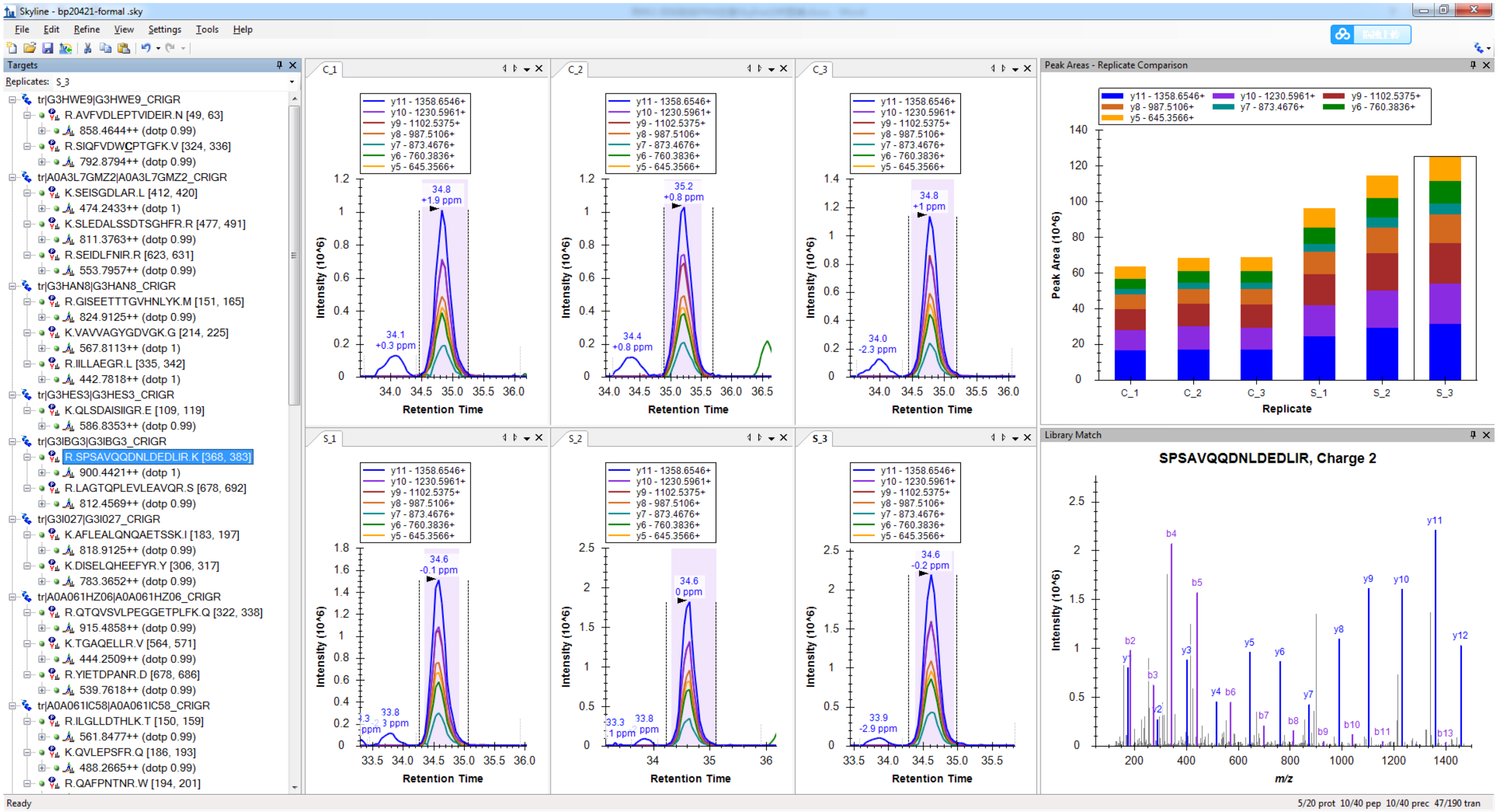

LAGTQPLEVLEAVQR

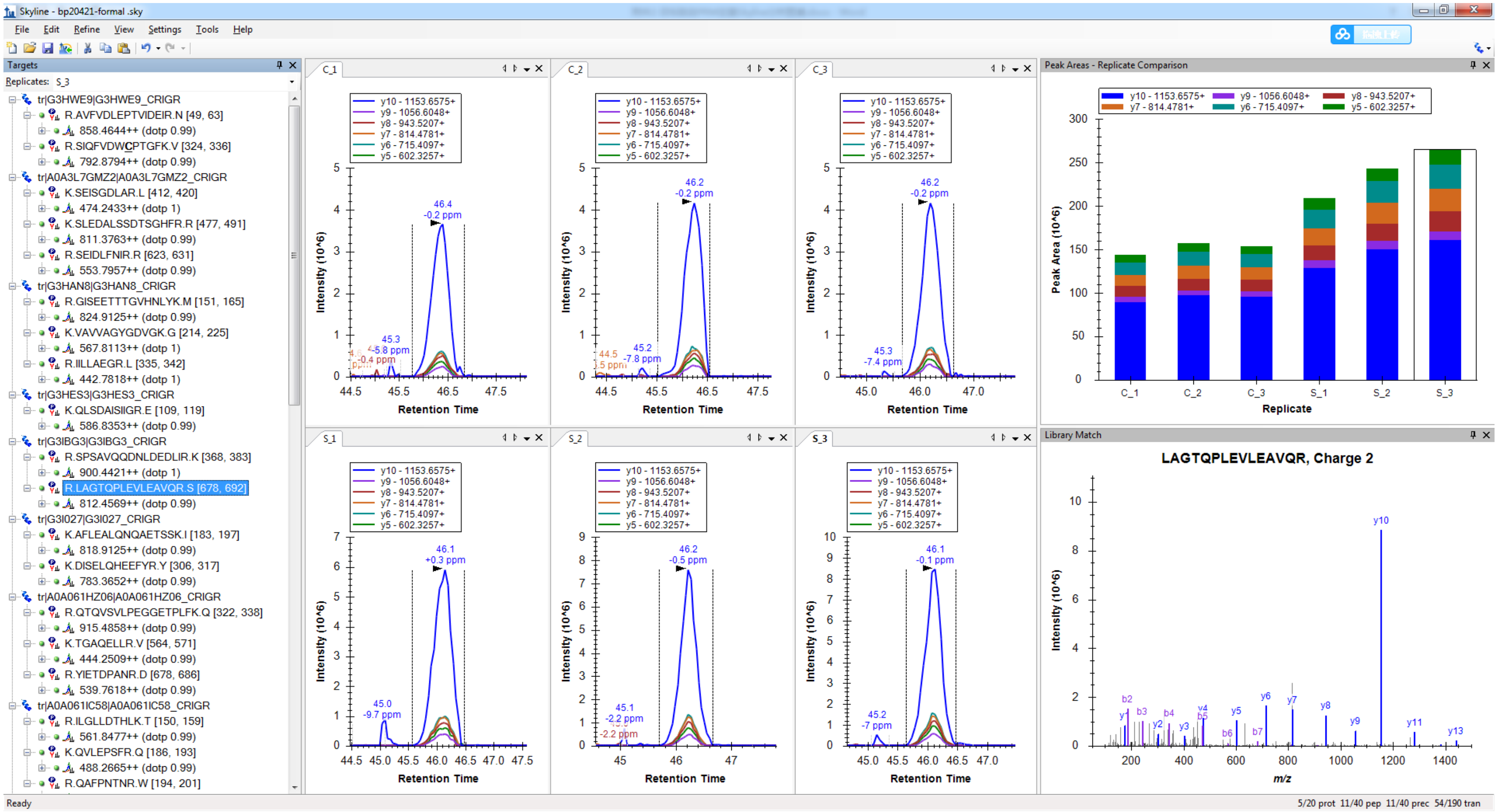

AFLEALQNQAETSSK

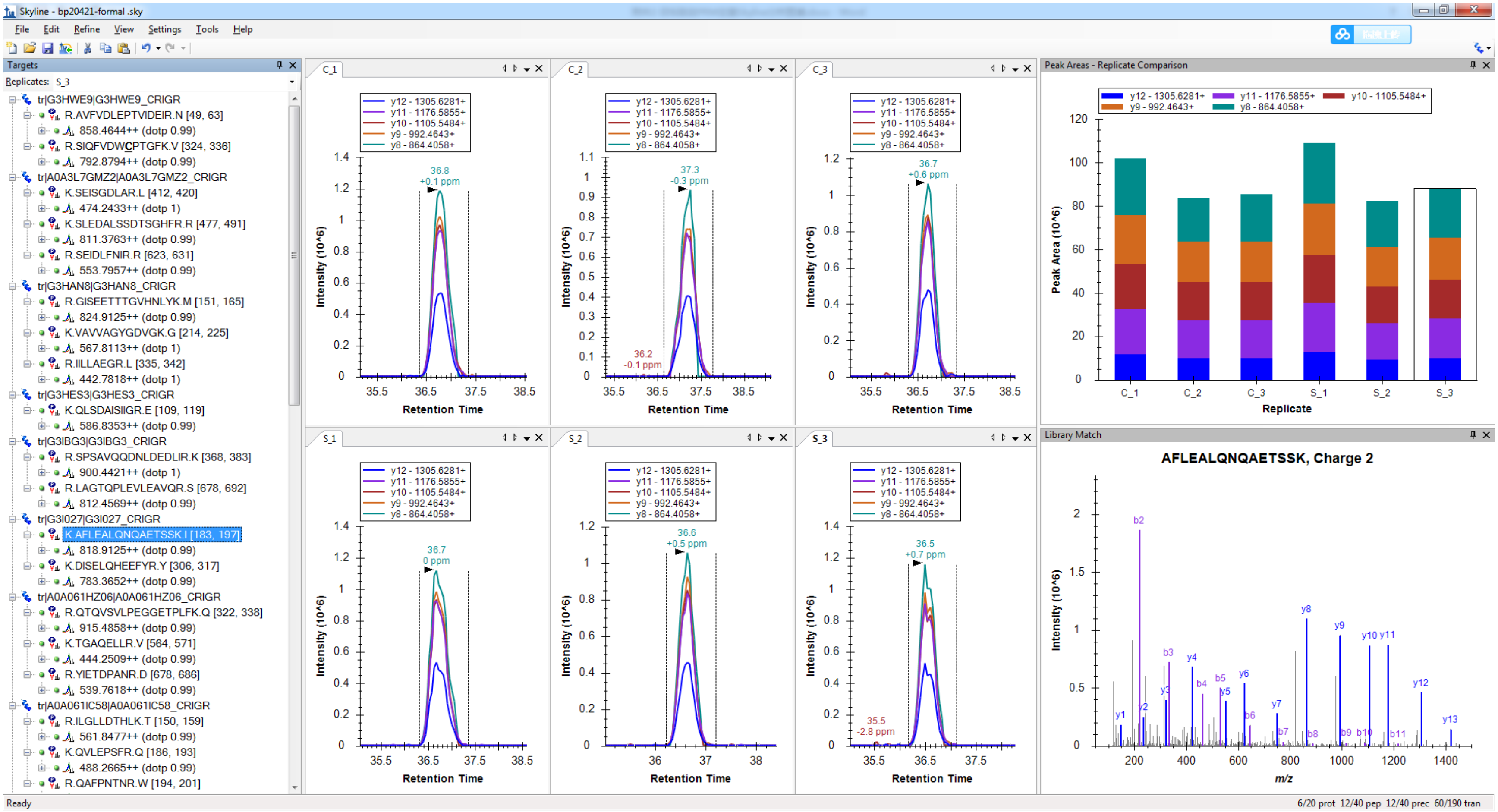

DISELQHEEFYR

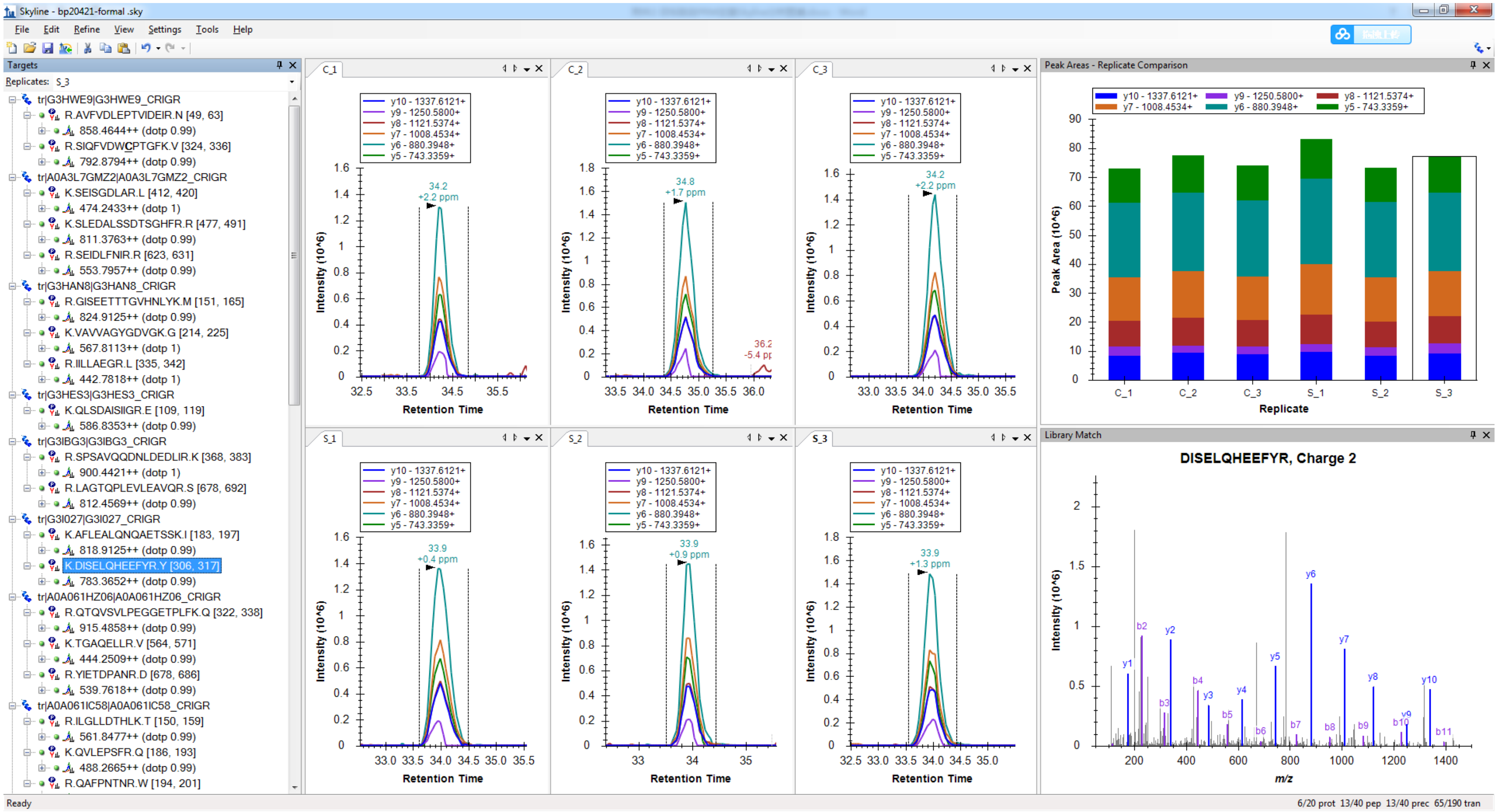

QTQVSVLPEGGETPLFK

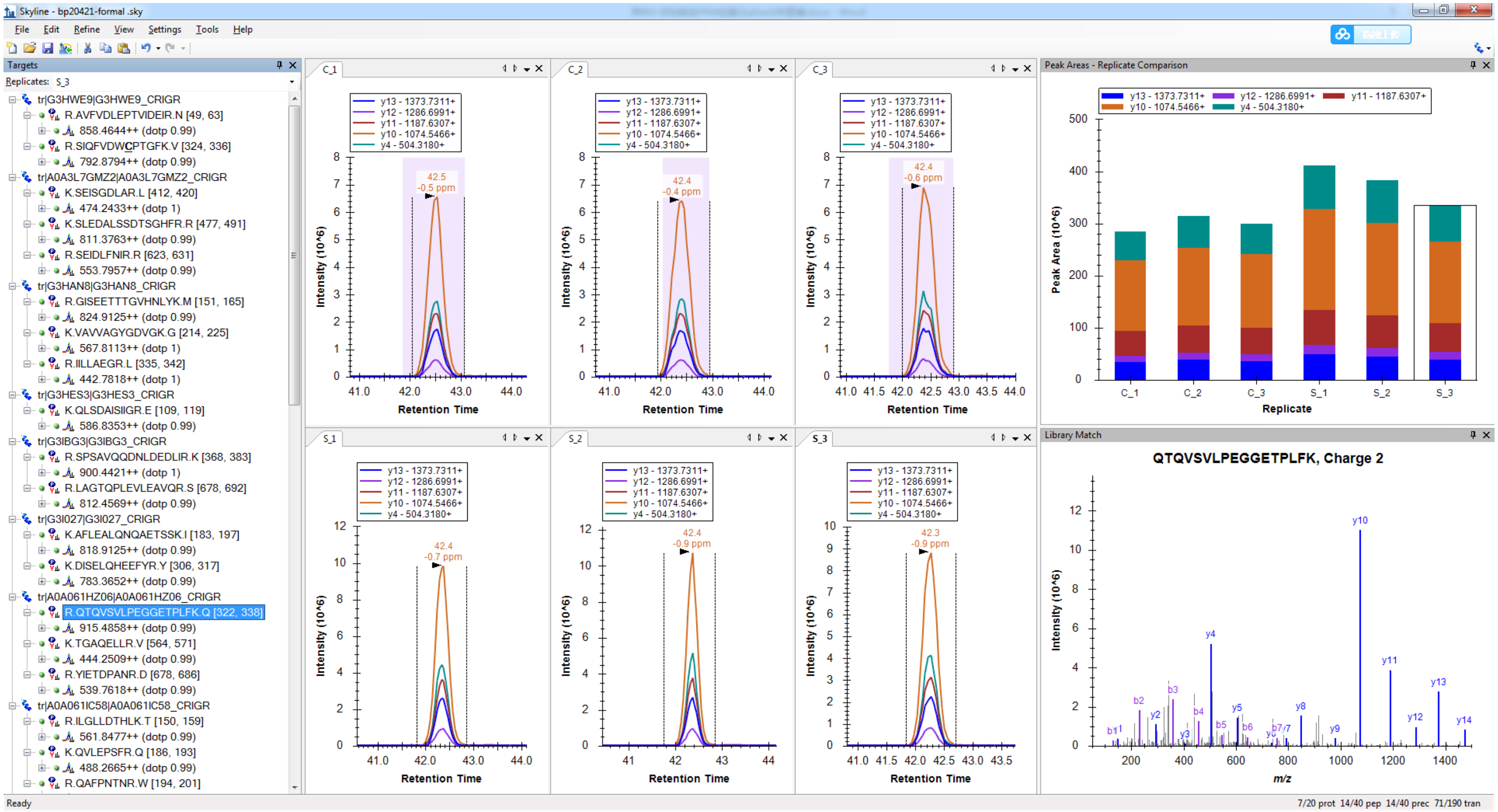

TGAQELLR

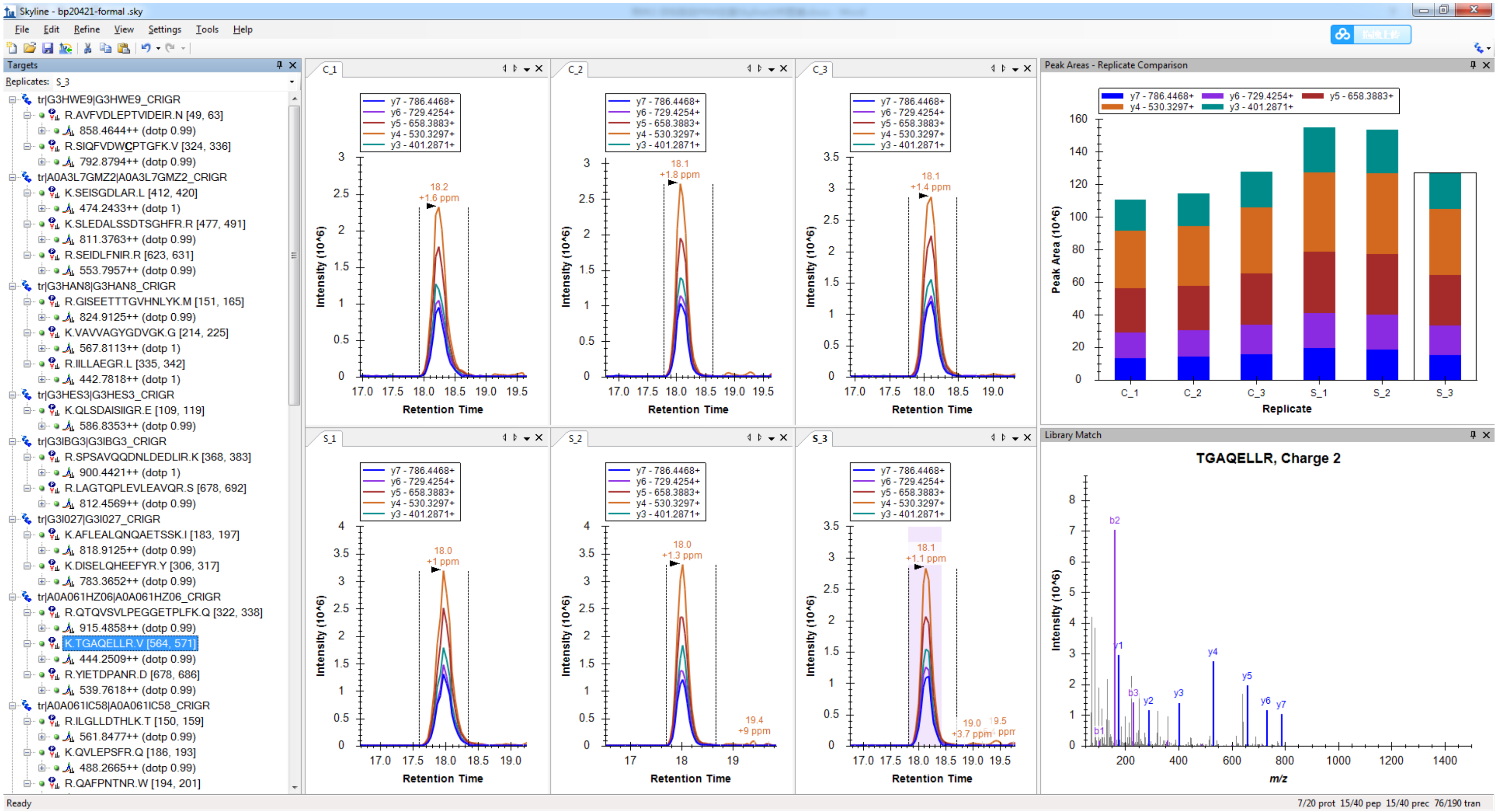

Skyline - bp20421-formal .sky
File Edit Refine View Settings Tools Help
Targets
Replicates: S_3
tr|G3HWE9|G3HWE9_CRIGR
R.AVFVDLEPTVIDEIR.N [49, 63]
858.4644++ (dotp 0.99)
R.SIQFVDWCPTGFK.V [324, 336]
792.8794++ (dotp 0.99)
tr|A0A3L7GMZ2|A0A3L7GMZ2_CRIGR
K.SEISGDLAR.L [412, 420]
474.2433++ (dotp 1)
K.SLEDALSSDTSGHFR.R [477, 491]
811.3763++ (dotp 0.99)
R.SEIDLFNIR.R [623, 631]
553.7957++ (dotp 0.99)
tr|G3HAN8|G3HAN8_CRIGR
R.GISEETTTGVHNLYK.M [151, 165]
824.9125++ (dotp 0.99)
K.VAVVAGYGDVGK.G [214, 225]
567.8113++ (dotp 1)
R.IILLAEGR.L [335, 342]
442.7818++ (dotp 1)
tr|G3HES3|G3HES3_CRIGR
K.QLSDAISIIGR.E [109, 119]
586.8353++ (dotp 0.99)
tr|G3IBG3|G3IBG3_CRIGR
R.SPSAVQQDNLDEDLIR.K [368, 383]
900.4421++ (dotp 1)
R.LAGTQPLEVLEAVQR.S [678, 692]
812.4569++ (dotp 0.99)
tr|G3I027|G3I027_CRIGR
K.AFLEALQNQAETSSK.I [183, 197]
818.9125++ (dotp 0.99)
K.DISELQHEEFYR.Y [306, 317]
783.3652++ (dotp 0.99)
tr|A0A061HZ06|A0A061HZ06_CRIGR
R.QTQVSVLPEGGETPLFK.Q [322, 338]
915.4858++ (dotp 0.99)
K.TGAQELLR.V [564, 571]
444.2509++ (dotp 0.99)
R.YIETDPANR.D [678, 686]
539.7618++ (dotp 0.99)
tr|A0A061IC58|A0A061IC58_CRIGR
R.ILGLLDTHLK.T [150, 159]
561.8477++ (dotp 0.99)
K.QVLEPSFR.Q [186, 193]
488.2665++ (dotp 0.99)
R.QAFPNTNR.W [194, 201]
C_1
C_2
C_3
S_1
S_2
S_3
y7 - 786.4468+
y6 - 729.4254+
y5 - 658.3883+
y4 - 530.3297+
y3 - 401.2871+
18.2 +1.6 ppm
18.1 +1.8 ppm
18.1 +1.4 ppm
18.0 +1 ppm
18.0 +1.3 ppm
19.4 +9 ppm
18.1 +1.1 ppm
19.0 +3.7 ppm
Intensity (10^6)
Retention Time
Peak Areas - Replicate Comparison
Peak Area (10^6)
Replicate
Library Match
TGAQELLR, Charge 2
b1
b2
b3
y1
y2
y3
y4
y5
y6
y7
m/z
Ready
7/20 prot 15/40 pep 15/40 prec 76/190 tran

YIETDPANR

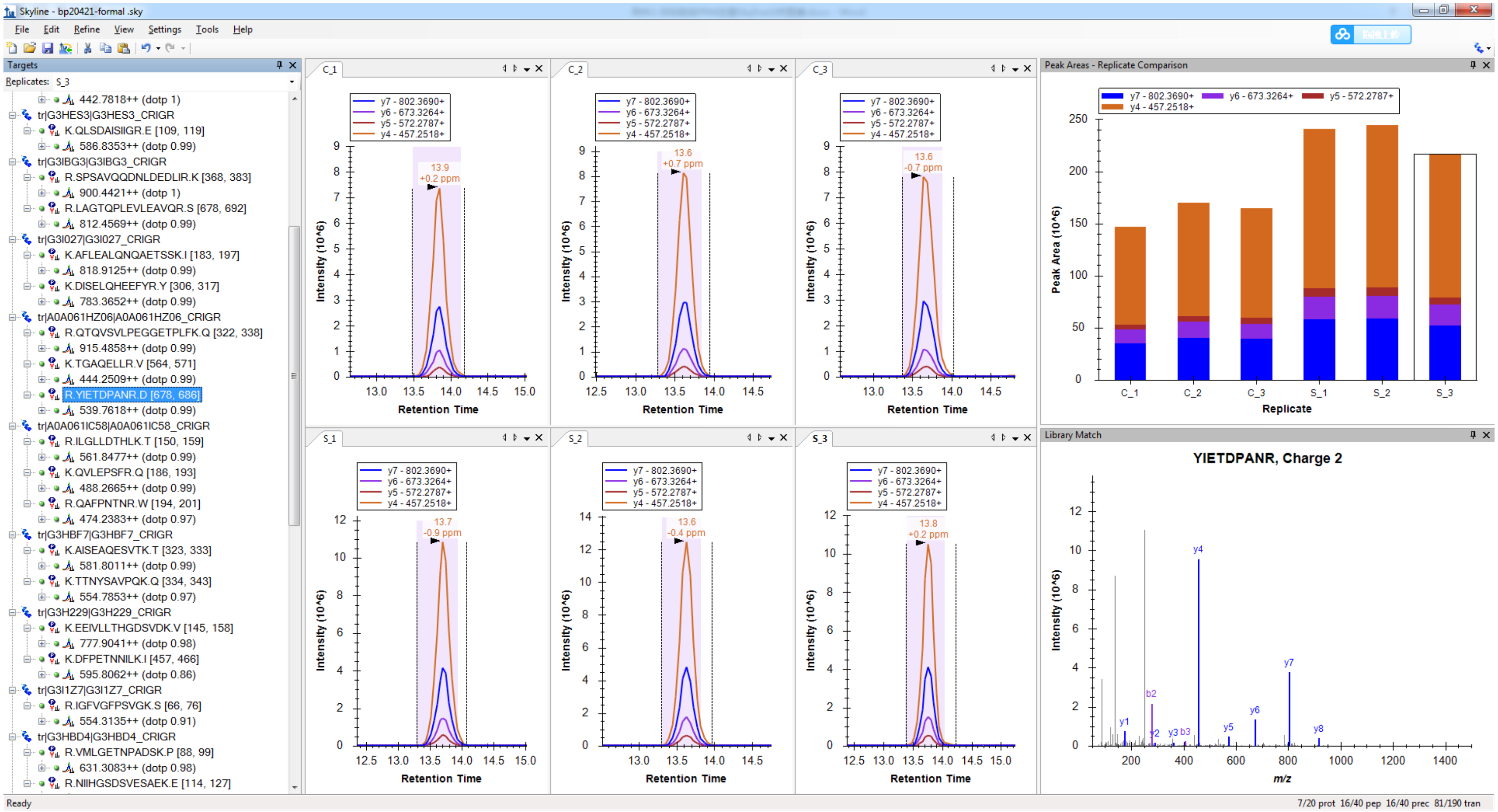

Skyline - bp20421-formal .sky
File Edit Refine View Settings Tools Help
Targets
Replicates: S_3
442.7818++ (dotp 1)
tr|G3HES3|G3HES3_CRIGR
K.QLSDAISIIGR.E [109, 119]
586.8353++ (dotp 0.99)
tr|G3IBG3|G3IBG3_CRIGR
R.SPSAVQQDNLDEDLIR.K [368, 383]
900.4421++ (dotp 1)
R.LAGTQPLEVLEAVQR.S [678, 692]
812.4569++ (dotp 0.99)
tr|G3I027|G3I027_CRIGR
K.AFLEALQNQAETSSK.I [183, 197]
818.9125++ (dotp 0.99)
K.DISELQHEEFYR.Y [306, 317]
783.3652++ (dotp 0.99)
tr|A0A061HZ06|A0A061HZ06_CRIGR
R.QTQVSVLPEGGETPLFK.Q [322, 338]
915.4858++ (dotp 0.99)
K.TGAQELLR.V [564, 571]
444.2509++ (dotp 0.99)
R.YIETDPANR.D [678, 686]
539.7618++ (dotp 0.99)
tr|A0A061IC58|A0A061IC58_CRIGR
R.ILGLLDTHLK.T [150, 159]
561.8477++ (dotp 0.99)
K.QVLEPSFR.Q [186, 193]
488.2665++ (dotp 0.99)
R.QAFPNTNR.W [194, 201]
474.2383++ (dotp 0.97)
tr|G3HBF7|G3HBF7_CRIGR
K.AISEAQESVTK.T [323, 333]
581.8011++ (dotp 0.99)
K.TTNYSAVPQK.Q [334, 343]
554.7853++ (dotp 0.97)
tr|G3H229|G3H229_CRIGR
K.EEIVLLTHGDSVDK.V [145, 158]
777.9041++ (dotp 0.98)
K.DFPETNNILK.I [457, 466]
595.8062++ (dotp 0.86)
tr|G3I1Z7|G3I1Z7_CRIGR
R.IGFVGFPSVGK.S [66, 76]
554.3135++ (dotp 0.91)
tr|G3HBD4|G3HBD4_CRIGR
R.VMLGETNPADSK.P [88, 99]
631.3083++ (dotp 0.98)
R.NIIHGSDSVESAEK.E [114, 127]
C_1
C_2
C_3
S_1
S_2
S_3
y7 - 802.3690+
y6 - 673.3264+
y5 - 572.2787+
y4 - 457.2518+
Intensity (10^6)
Retention Time
13.9 +0.2 ppm
13.6 +0.7 ppm
13.6 -0.7 ppm
13.7 -0.9 ppm
13.6 -0.4 ppm
13.8 +0.2 ppm
Peak Areas - Replicate Comparison
Peak Area (10^6)
Replicate
Library Match
YIETDPANR, Charge 2
y1 b2 y2 y3 b3 y4 y5 y6 y7 y8
m/z
Ready
7/20 prot 16/40 pep 16/40 prec 81/190 tran

ILGLLDTHLK

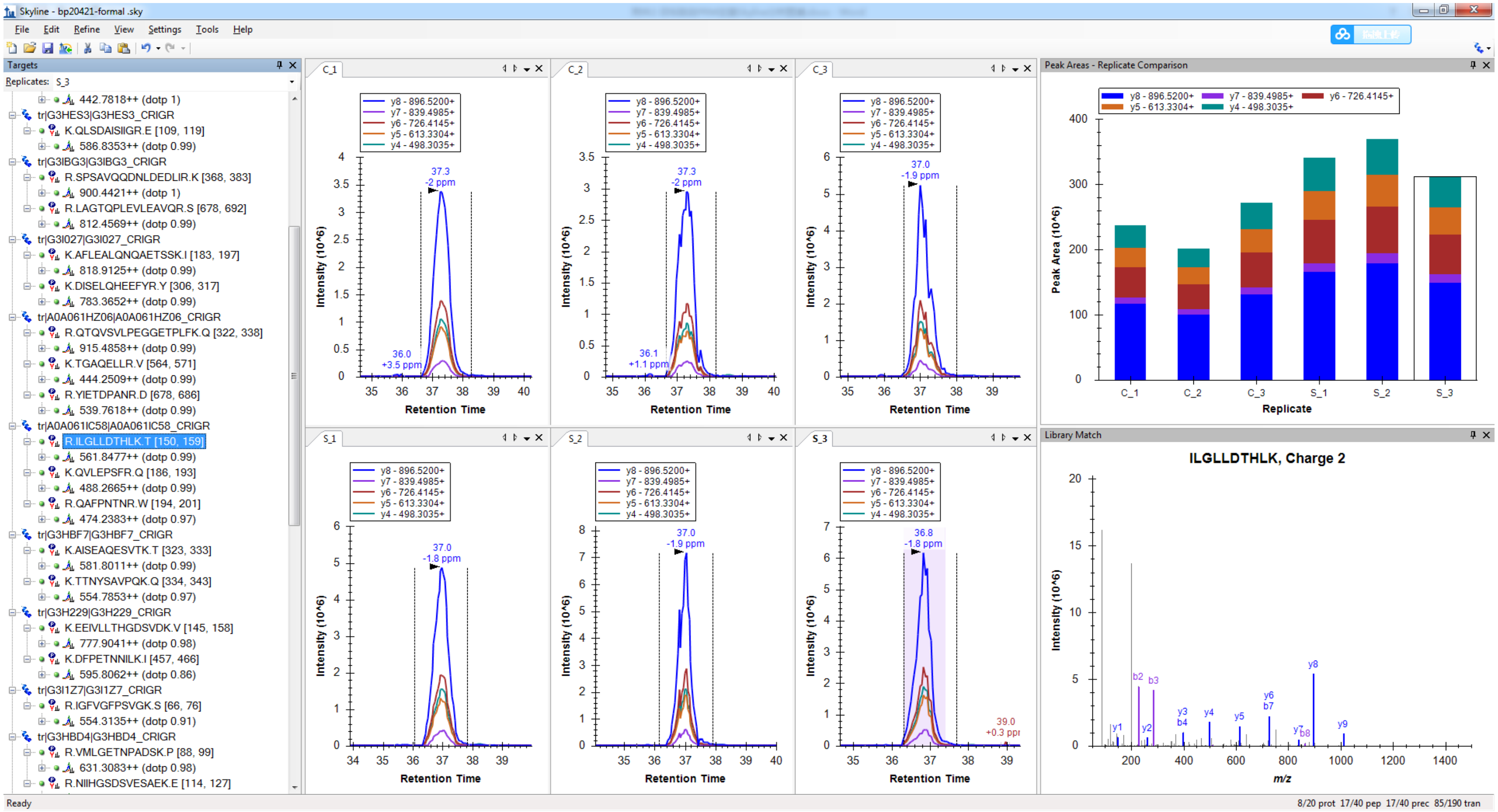

Skyline - bp20421-formal .sky
File Edit Refine View Settings Tools Help
Targets
Replicates: S_3
442.7818++ (dotp 1)
tr|G3HES3|G3HES3_CRIGR
K.QLSDAISIIGR.E [109, 119]
586.8353++ (dotp 0.99)
tr|G3IBG3|G3IBG3_CRIGR
R.SPSAVQQDNLDEDLIR.K [368, 383]
900.4421++ (dotp 1)
R.LAGTQPLEVLAEVQR.S [678, 692]
812.4569++ (dotp 0.99)
tr|G3I027|G3I027_CRIGR
K.AFLEALQNQAETSSK.I [183, 197]
818.9125++ (dotp 0.99)
K.DISELQHEEFYR.Y [306, 317]
783.3652++ (dotp 0.99)
tr|A0A061HZ06|A0A061HZ06_CRIGR
R.QTQVSVLPEGGETPLFK.Q [322, 338]
915.4858++ (dotp 0.99)
K.TGAQELLR.V [564, 571]
444.2509++ (dotp 0.99)
R.YIETDPANR.D [678, 686]
539.7618++ (dotp 0.99)
tr|A0A061IC58|A0A061IC58_CRIGR
R.ILGLLDTHLK.T [150, 159]
561.8477++ (dotp 0.99)
K.QVLEPSFR.Q [186, 193]
488.2665++ (dotp 0.99)
R.QAFPNTNR.W [194, 201]
474.2383++ (dotp 0.97)
tr|G3HBF7|G3HBF7_CRIGR
K.AISEAQESVTK.T [323, 333]
581.8011++ (dotp 0.99)
K.TTNYSAVPQK.Q [334, 343]
554.7853++ (dotp 0.97)
tr|G3H229|G3H229_CRIGR
K.EEIVLLTHGDSVDK.V [145, 158]
777.9041++ (dotp 0.98)
K.DFPETNNILK.I [457, 466]
595.8062++ (dotp 0.86)
tr|G3I1Z7|G3I1Z7_CRIGR
R.IGFVGFPSVGK.S [66, 76]
554.3135++ (dotp 0.91)
tr|G3HBD4|G3HBD4_CRIGR
R.VMLGETNPADSK.P [88, 99]
631.3083++ (dotp 0.98)
R.NIIHGSDSVESAEK.E [114, 127]
C_1
C_2
C_3
S_1
S_2
S_3
y8 - 896.5200+
y7 - 839.4985+
y6 - 726.4145+
y5 - 613.3304+
y4 - 498.3035+
Intensity (10^6)
Retention Time
37.3 -2 ppm
36.0 +3.5 ppm
37.3 -2 ppm
36.1 +1.1 ppm
37.0 -1.9 ppm
37.0 -1.8 ppm
37.0 -1.9 ppm
36.8 -1.8 ppm
39.0 +0.3 ppi
Peak Areas - Replicate Comparison
Peak Area (10^6)
Replicate
Library Match
ILGLLDTHLK, Charge 2
m/z
y1 y2 b2 b3 y3 b4 y4 y5 y6 b7 y7 b8 y8 y9
Ready
8/20 prot 17/40 pep 17/40 prec 85/190 tran

QVLEPSFR

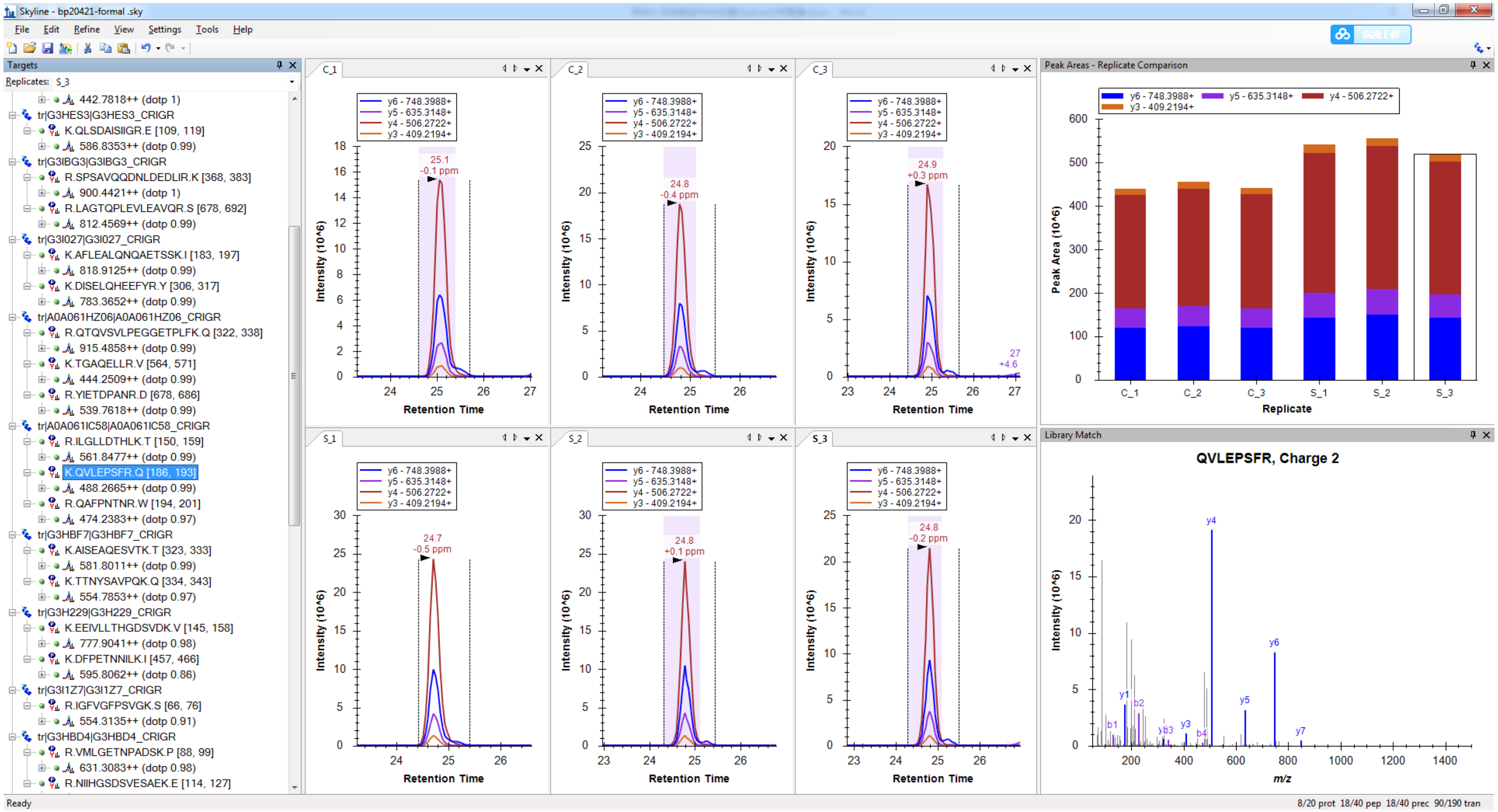

Skyline - bp20421-formal .sky
File Edit Refine View Settings Tools Help
Targets
Replicates: S_3
442.7818++ (dotp 1)
tr|G3HES3|G3HES3_CRIGR
K.QLSDAISIIGR.E [109, 119]
586.8353++ (dotp 0.99)
tr|G3IBG3|G3IBG3_CRIGR
R.SPSAVQQDNLDEDLIR.K [368, 383]
900.4421++ (dotp 1)
R.LAGTQPLEVLEAVQR.S [678, 692]
812.4569++ (dotp 0.99)
tr|G3I027|G3I027_CRIGR
K.AFLEALQNQAETSSK.I [183, 197]
818.9125++ (dotp 0.99)
K.DISELQHEEFYR.Y [306, 317]
783.3652++ (dotp 0.99)
tr|A0A061HZ06|A0A061HZ06_CRIGR
R.QTQVSVLPEGGETPLFK.Q [322, 338]
915.4858++ (dotp 0.99)
K.TGAQELLR.V [564, 571]
444.2509++ (dotp 0.99)
R.YIETDPANR.D [678, 686]
539.7618++ (dotp 0.99)
tr|A0A061IC58|A0A061IC58_CRIGR
R.ILGLLDTHLK.T [150, 159]
561.8477++ (dotp 0.99)
K.QVLEPSFR.Q [186, 193]
488.2665++ (dotp 0.99)
R.QAFPNTNR.W [194, 201]
474.2383++ (dotp 0.97)
tr|G3HBF7|G3HBF7_CRIGR
K.AISEAQESVTK.T [323, 333]
581.8011++ (dotp 0.99)
K.TTNYSAVPQK.Q [334, 343]
554.7853++ (dotp 0.97)
tr|G3H229|G3H229_CRIGR
K.EEIVLLTHGDSVDK.V [145, 158]
777.9041++ (dotp 0.98)
K.DFPETNNILK.I [457, 466]
595.8062++ (dotp 0.86)
tr|G3I1Z7|G3I1Z7_CRIGR
R.IGFVGFPSVGK.S [66, 76]
554.3135++ (dotp 0.91)
tr|G3HBD4|G3HBD4_CRIGR
R.VMLGETNPADSK.P [88, 99]
631.3083++ (dotp 0.98)
R.NIIHGSDSVESAEK.E [114, 127]
C_1
C_2
C_3
S_1
S_2
S_3
y6 - 748.3988+
y5 - 635.3148+
y4 - 506.2722+
y3 - 409.2194+
25.1
-0.1 ppm
24.8
-0.4 ppm
24.9
+0.3 ppm
27
+4.6
24.7
-0.5 ppm
24.8
+0.1 ppm
24.8
-0.2 ppm
Intensity (10^6)
Retention Time
Peak Areas - Replicate Comparison
Peak Area (10^6)
Replicate
Library Match
QVLEPSFR, Charge 2
y4
y6
y5
y7
y1
b2
b1
y3
b3
b4
m/z
Ready
8/20 prot 18/40 pep 18/40 prec 90/190 tran

QAFPNTNR

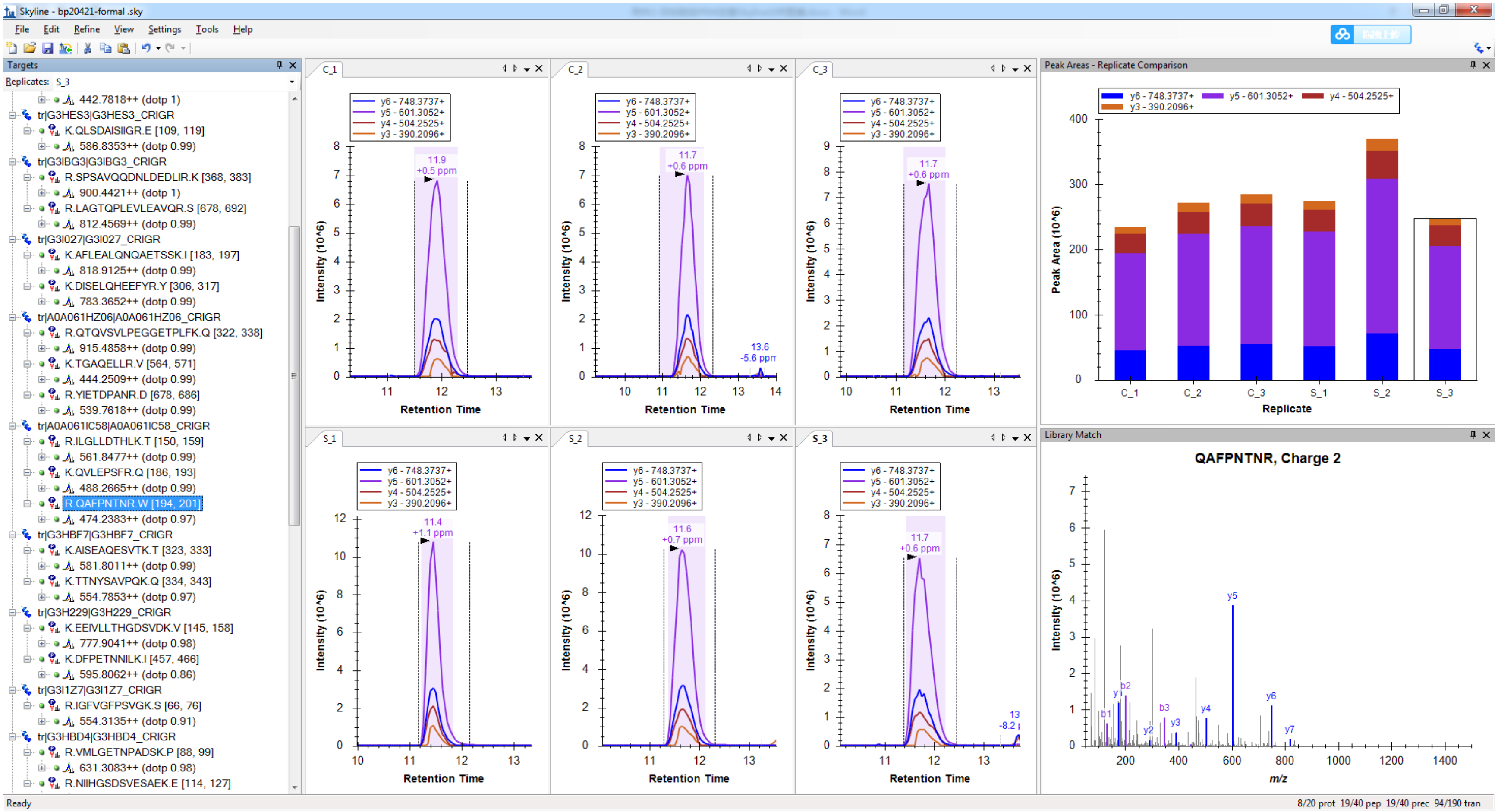

Skyline - bp20421-formal .sky
File
Edit
Refine
View
Settings
Tools
Help
Targets
Replicates: S_3
442.7818++ (dotp 1)
tr|G3HES3|G3HES3_CRIGR
K.QLSDAISIIGR.E [109, 119]
586.8353++ (dotp 0.99)
tr|G3IBG3|G3IBG3_CRIGR
R.SPSAVQQDNLDEDLIR.K [368, 383]
900.4421++ (dotp 1)
R.LAGTQPLEVLEAVQR.S [678, 692]
812.4569++ (dotp 0.99)
tr|G3I027|G3I027_CRIGR
K.AFLEALQNQAETSSK.I [183, 197]
818.9125++ (dotp 0.99)
K.DISELQHEEFYR.Y [306, 317]
783.3652++ (dotp 0.99)
tr|A0A061HZ06|A0A061HZ06_CRIGR
R.QTQVSVLPEGGETPLFK.Q [322, 338]
915.4858++ (dotp 0.99)
K.TGAQELLR.V [564, 571]
444.2509++ (dotp 0.99)
R.YIETDPANR.D [678, 686]
539.7618++ (dotp 0.99)
tr|A0A061IC58|A0A061IC58_CRIGR
R.ILGLLDTHLK.T [150, 159]
561.8477++ (dotp 0.99)
K.QVLEPSFR.Q [186, 193]
488.2665++ (dotp 0.99)
R.QAFPNTNR.W [194, 201]
474.2383++ (dotp 0.97)
tr|G3HBF7|G3HBF7_CRIGR
K.AISEAQESVTK.T [323, 333]
581.8011++ (dotp 0.99)
K.TTNYSAVPQK.Q [334, 343]
554.7853++ (dotp 0.97)
tr|G3H229|G3H229_CRIGR
K.EEIVLLTHGDSVDK.V [145, 158]
777.9041++ (dotp 0.98)
K.DFPETNNILK.I [457, 466]
595.8062++ (dotp 0.86)
tr|G3I1Z7|G3I1Z7_CRIGR
R.IGFVGFPSVGK.S [66, 76]
554.3135++ (dotp 0.91)
tr|G3HBD4|G3HBD4_CRIGR
R.VMLGETNPADSK.P [88, 99]
631.3083++ (dotp 0.98)
R.NIIHGSDSVESAEK.E [114, 127]
C_1
C_2
C_3
S_1
S_2
S_3
y6 - 748.3737+
y5 - 601.3052+
y4 - 504.2525+
y3 - 390.2096+
11.9
+0.5 ppm
11.7
+0.6 ppm
13.6
-5.6 ppm
11.4
+1.1 ppm
11.6
+0.7 ppm
+0.6 ppm
Intensity (10^6)
Retention Time
Peak Areas - Replicate Comparison
Peak Area (10^6)
Replicate
Library Match
QAFPNTNR, Charge 2
b1
b2
b3
y2
y3
y4
y5
y6
y7
m/z
Ready
8/20 prot 19/40 pep 19/40 prec 94/190 tran

AISEAQESVTK

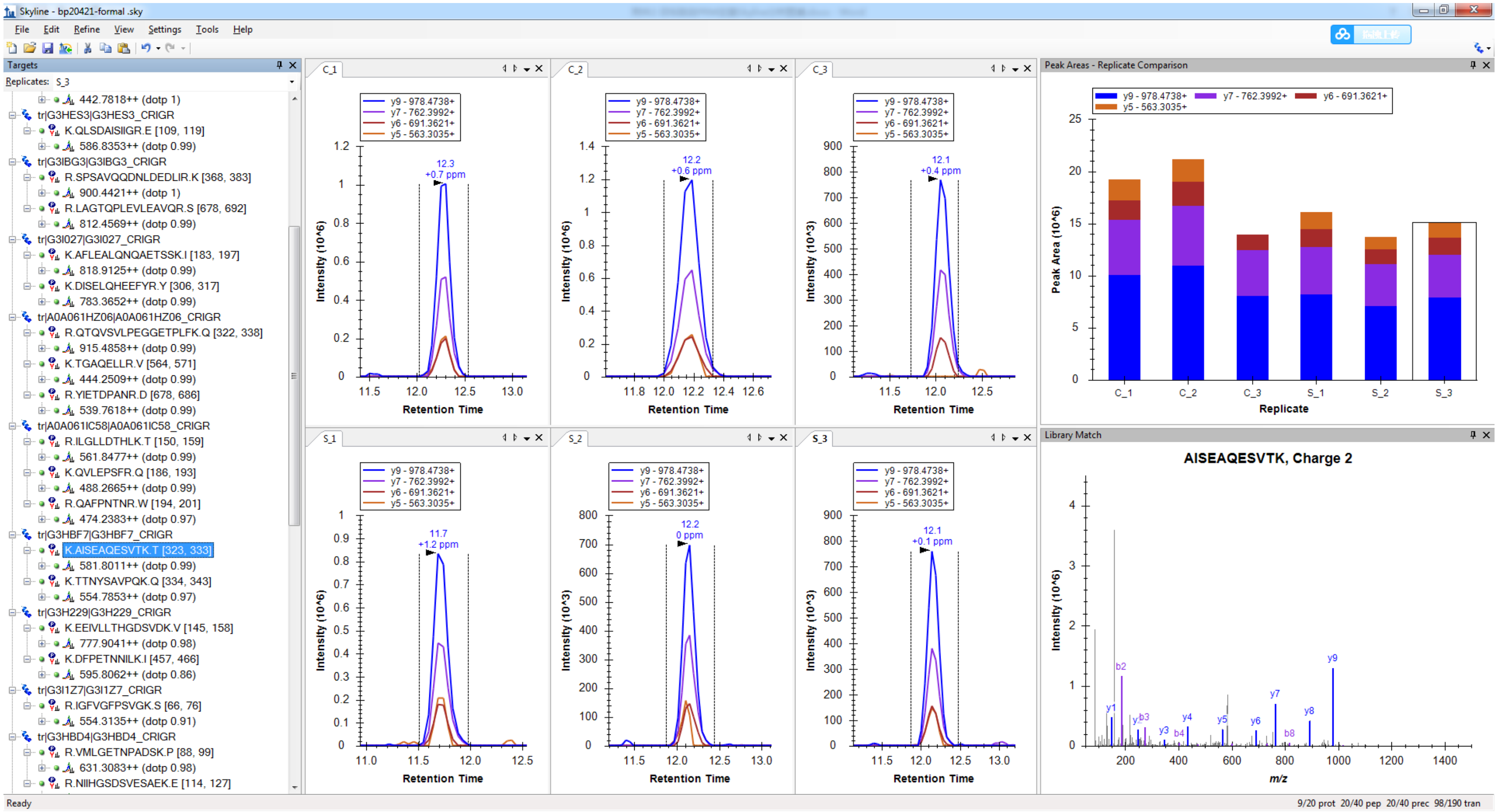

TTNYSAVPQK

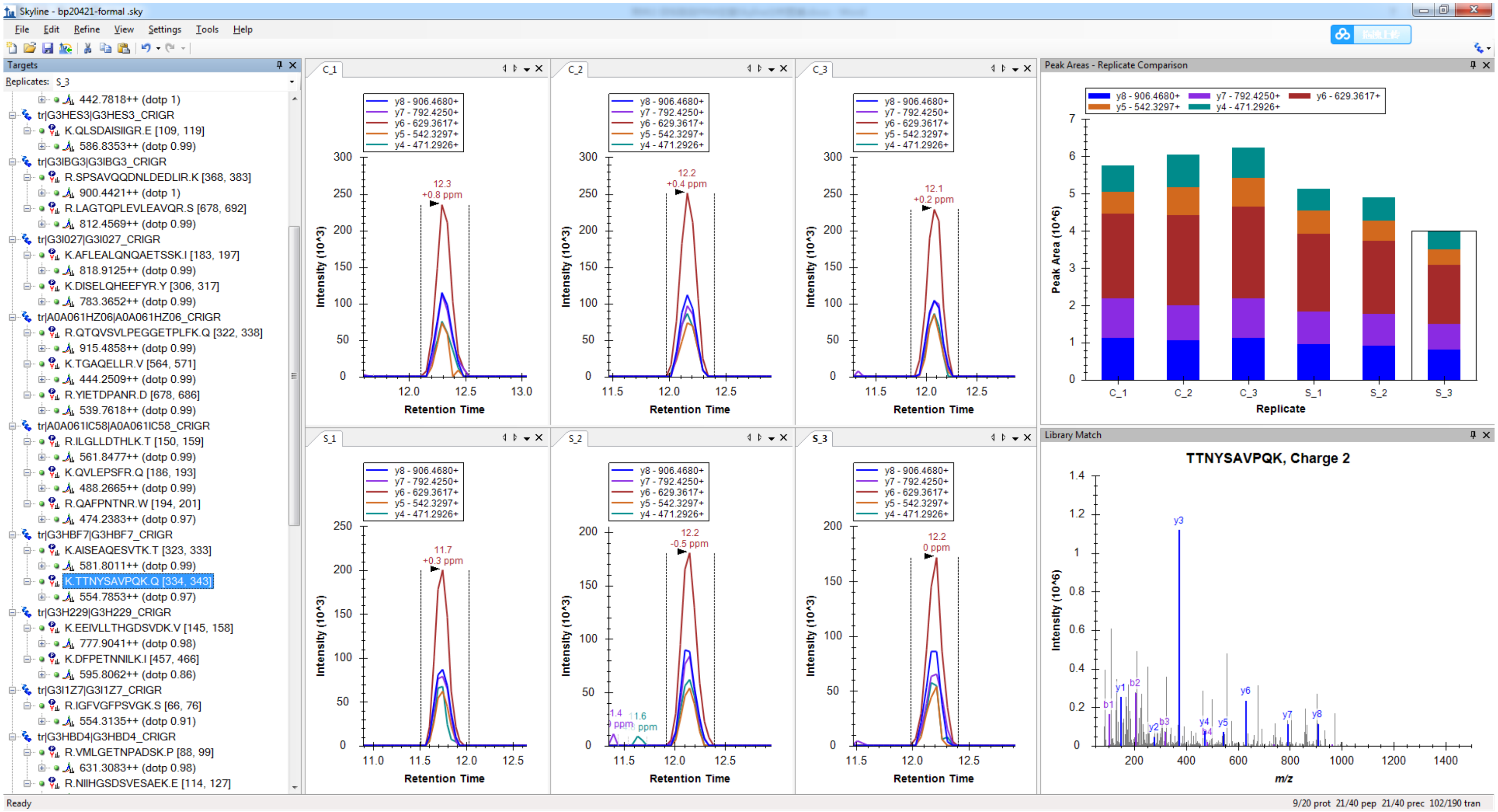

Skyline - bp20421-formal .sky
File Edit Refine View Settings Tools Help
Targets
Replicates: S_3
442.7818++ (dotp 1)
tr|G3HES3|G3HES3_CRIGR
K.QLSDAISIIGR.E [109, 119]
586.8353++ (dotp 0.99)
tr|G3IBG3|G3IBG3_CRIGR
R.SPSAVQQDNLDEDLIR.K [368, 383]
900.4421++ (dotp 1)
R.LAGTQPLEVLEAVQR.S [678, 692]
812.4569++ (dotp 0.99)
tr|G3I027|G3I027_CRIGR
K.AFLEALQNQAETSSK.I [183, 197]
818.9125++ (dotp 0.99)
K.DISELQHEEFYR.Y [306, 317]
783.3652++ (dotp 0.99)
tr|A0A061HZ06|A0A061HZ06_CRIGR
R.QTQVSVLPEGGETPLFK.Q [322, 338]
915.4858++ (dotp 0.99)
K.TGAQELLR.V [564, 571]
444.2509++ (dotp 0.99)
R.YIETDPANR.D [678, 686]
539.7618++ (dotp 0.99)
tr|A0A061IC58|A0A061IC58_CRIGR
R.ILGLLDTHLK.T [150, 159]
561.8477++ (dotp 0.99)
K.QVLEPSFR.Q [186, 193]
488.2665++ (dotp 0.99)
R.QAFPNTNR.W [194, 201]
474.2383++ (dotp 0.97)
tr|G3HBF7|G3HBF7_CRIGR
K.AISEAQESVTK.T [323, 333]
581.8011++ (dotp 0.99)
K.TTNYSAVPQK.Q [334, 343]
554.7853++ (dotp 0.97)
tr|G3H229|G3H229_CRIGR
K.EEIVLLTHGDSVDK.V [145, 158]
777.9041++ (dotp 0.98)
K.DFPETNNILK.I [457, 466]
595.8062++ (dotp 0.86)
tr|G3I1Z7|G3I1Z7_CRIGR
R.IGFVGFPSVGK.S [66, 76]
554.3135++ (dotp 0.91)
tr|G3HBD4|G3HBD4_CRIGR
R.VMLGETNPADSK.P [88, 99]
631.3083++ (dotp 0.98)
R.NIIHGSDSVESAEK.E [114, 127]
C_1
C_2
C_3
S_1
S_2
S_3
y8 - 906.4680+
y7 - 792.4250+
y6 - 629.3617+
y5 - 542.3297+
y4 - 471.2926+
Intensity (10^3)
Retention Time
12.3 +0.8 ppm
12.2 +0.4 ppm
12.1 +0.2 ppm
11.7 +0.3 ppm
12.2 -0.5 ppm
12.2 0 ppm
Peak Areas - Replicate Comparison
Peak Area (10^6)
Replicate
Library Match
TTNYSAVPQK, Charge 2
Intensity (10^6)
m/z
Ready
9/20 prot 21/40 pep 21/40 prec 102/190 tran

EEIVLLTHGDSVDK

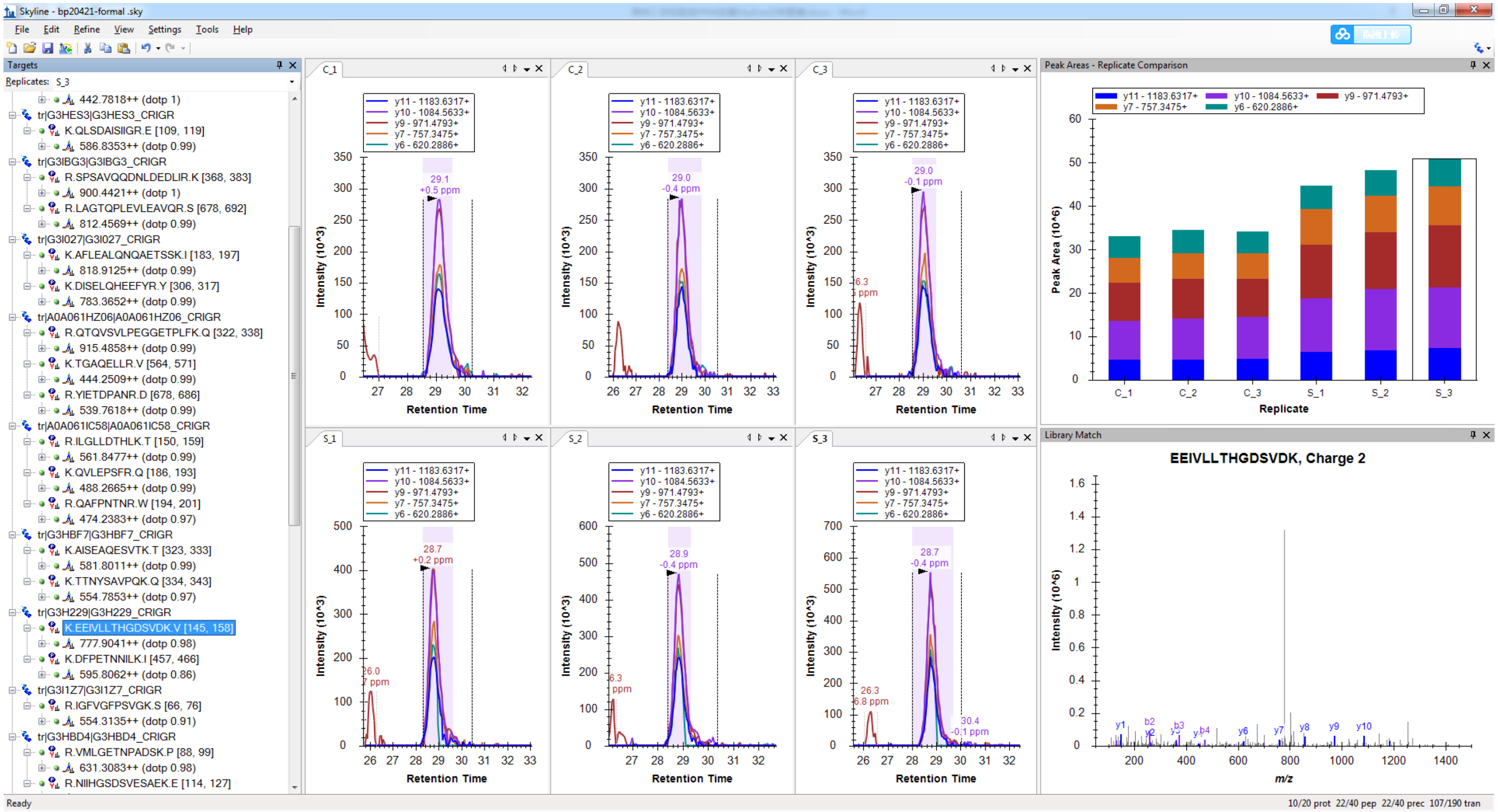

DFPETNNILK

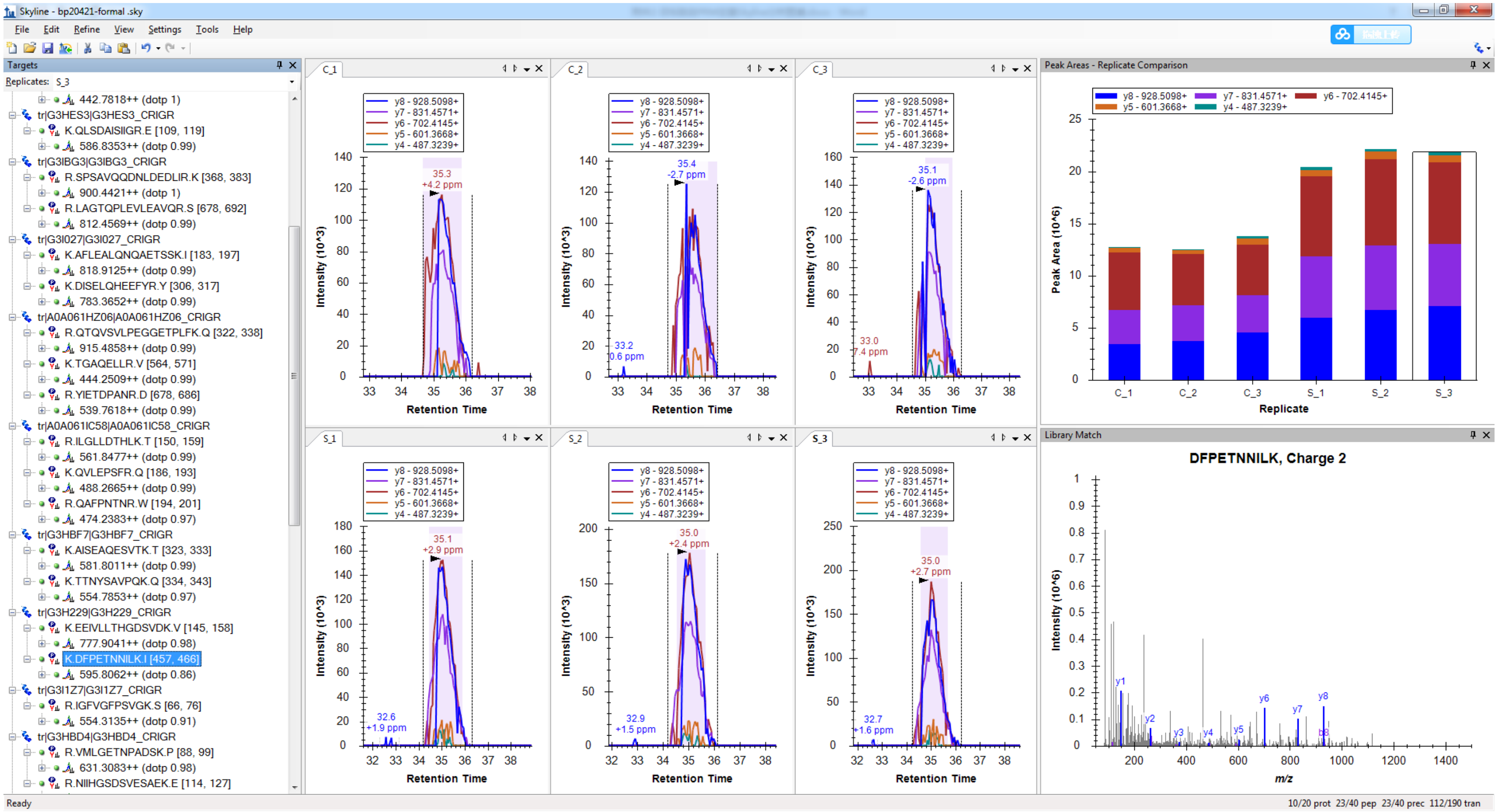

Skyline - bp20421-formal .sky
File Edit Refine View Settings Tools Help
Targets
Replicates: S_3
442.7818++ (dotp 1)
tr|G3HES3|G3HES3_CRIGR
K.QLSDAISIIGR.E [109, 119]
586.8353++ (dotp 0.99)
tr|G3IBG3|G3IBG3_CRIGR
R.SPSAVQQDNLDEDLIR.K [368, 383]
900.4421++ (dotp 1)
R.LAGTQPLEVLEAVQR.S [678, 692]
812.4569++ (dotp 0.99)
tr|G3I027|G3I027_CRIGR
K.AFLEALQNQAETSSK.I [183, 197]
818.9125++ (dotp 0.99)
K.DISELQHEEFYR.Y [306, 317]
783.3652++ (dotp 0.99)
tr|A0A061HZ06|A0A061HZ06_CRIGR
R.QTQVSVLPEGGETPLFK.Q [322, 338]
915.4858++ (dotp 0.99)
K.TGAQELLR.V [564, 571]
444.2509++ (dotp 0.99)
R.YIETDPANR.D [678, 686]
539.7618++ (dotp 0.99)
tr|A0A061IC58|A0A061IC58_CRIGR
R.ILGLLDTHLK.T [150, 159]
561.8477++ (dotp 0.99)
K.QVLEPSFR.Q [186, 193]
488.2665++ (dotp 0.99)
R.QAFPNTNR.W [194, 201]
474.2383++ (dotp 0.97)
tr|G3HBF7|G3HBF7_CRIGR
K.AISEAQESVTK.T [323, 333]
581.8011++ (dotp 0.99)
K.TTNYSAVPQK.Q [334, 343]
554.7853++ (dotp 0.97)
tr|G3H229|G3H229_CRIGR
K.EEIVLLTHGDSVDK.V [145, 158]
777.9041++ (dotp 0.98)
K.DFPETNNILK.I [457, 466]
595.8062++ (dotp 0.86)
tr|G3I1Z7|G3I1Z7_CRIGR
R.IGFVGFPSVGK.S [66, 76]
554.3135++ (dotp 0.91)
tr|G3HBD4|G3HBD4_CRIGR
R.VMLGETNPADSK.P [88, 99]
631.3083++ (dotp 0.98)
R.NIIHGSDSVESAEK.E [114, 127]
C_1
C_2
C_3
S_1
S_2
S_3
y8 - 928.5098+
y7 - 831.4571+
y6 - 702.4145+
y5 - 601.3668+
y4 - 487.3239+
Intensity (10^3)
Retention Time
35.3 +4.2 ppm
35.4 -2.7 ppm
33.2 0.6 ppm
35.1 -2.6 ppm
33.0 7.4 ppm
35.1 +2.9 ppm
32.6 +1.9 ppm
35.0 +2.4 ppm
32.9 +1.5 ppm
35.0 +2.7 ppm
32.7 +1.6 ppm
Peak Areas - Replicate Comparison
Peak Area (10^6)
Replicate
Library Match
DFPETNNILK, Charge 2
Intensity (10^6)
m/z
y1
y2
y3
y4
y5
y6
y7
y8
Ready
10/20 prot 23/40 pep 23/40 prec 112/190 tran

IGFVGFPSVGK

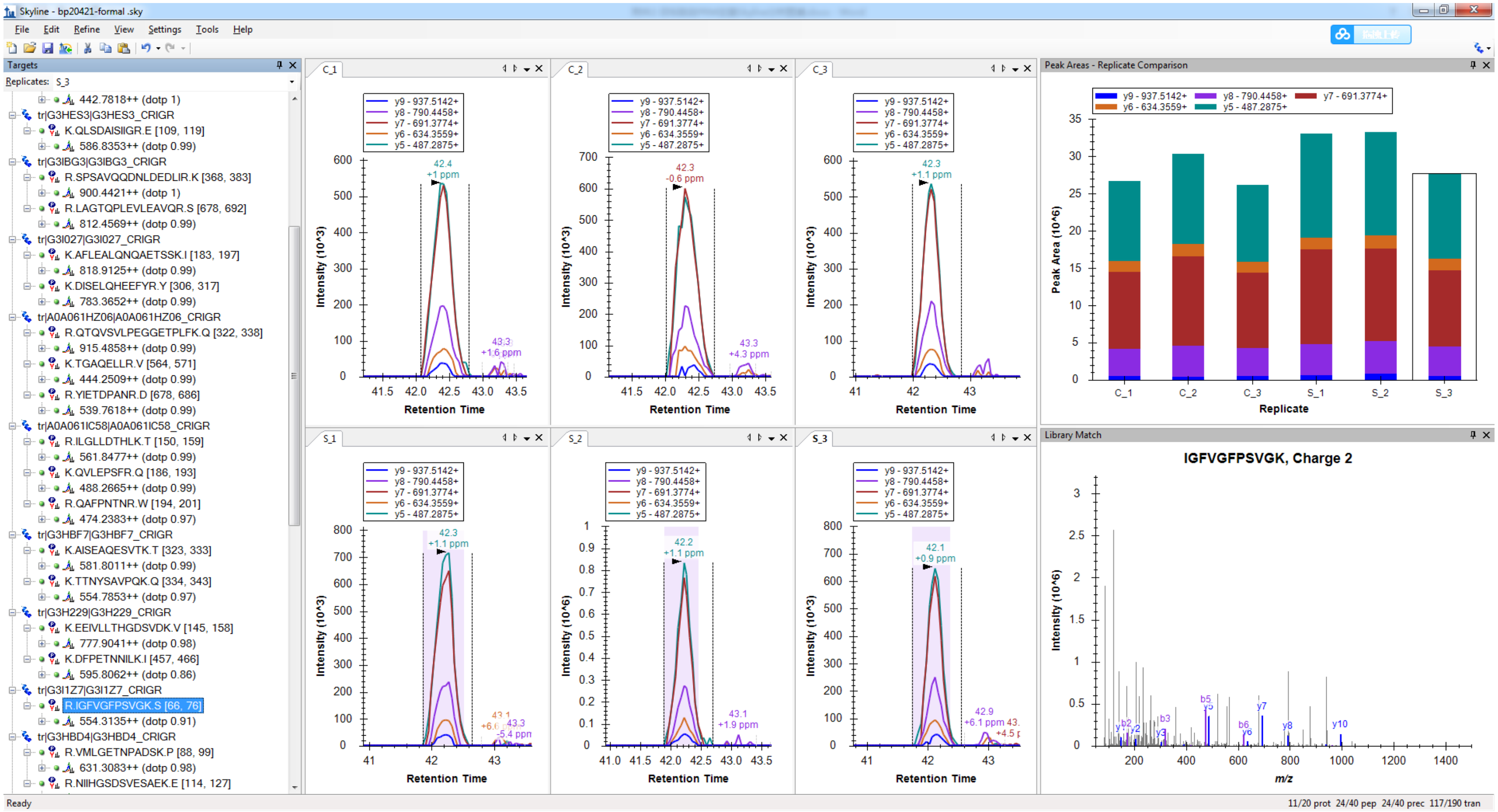

VMLGETNPADSK

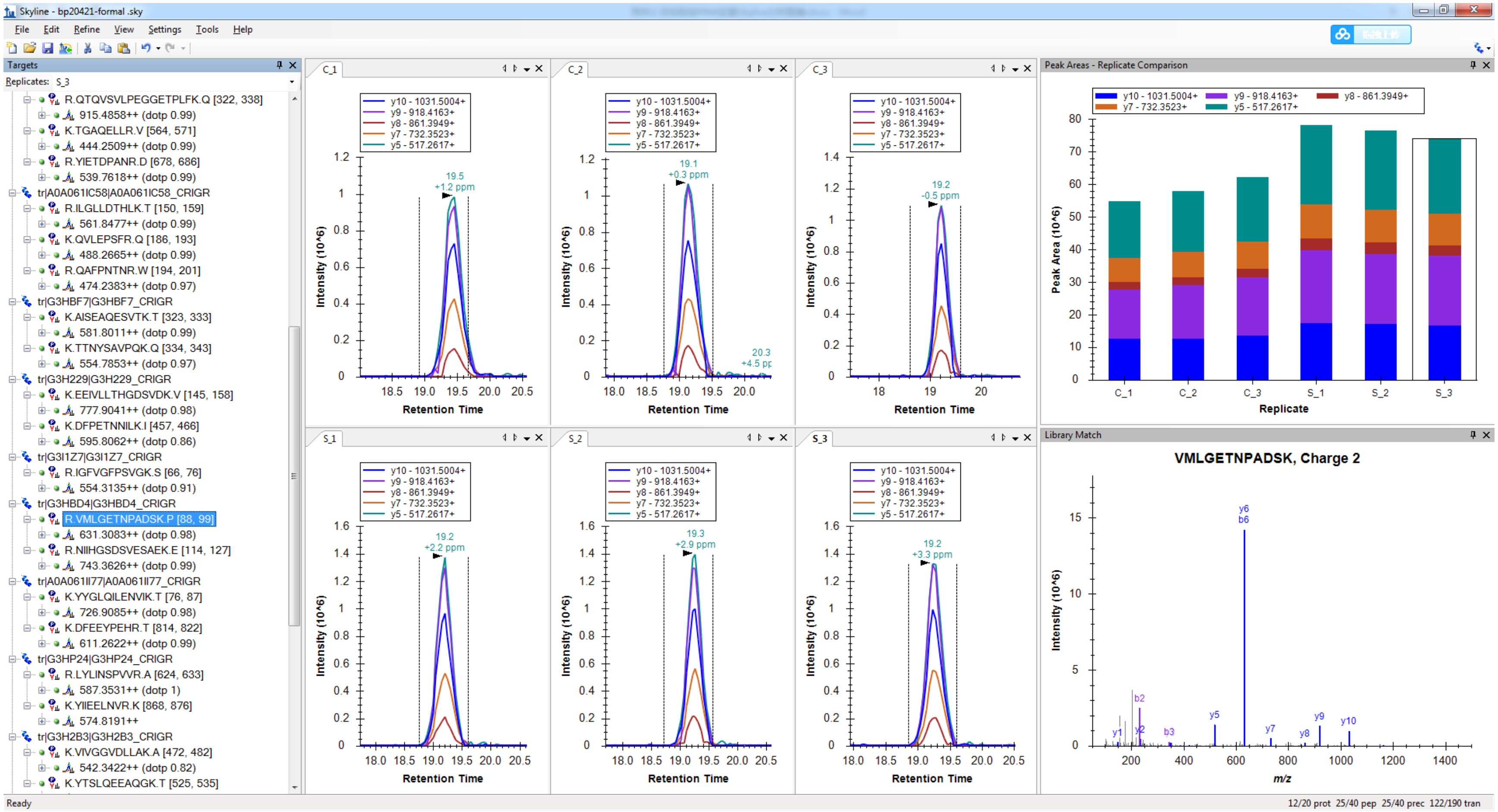

NIIHGSDSVESAEK

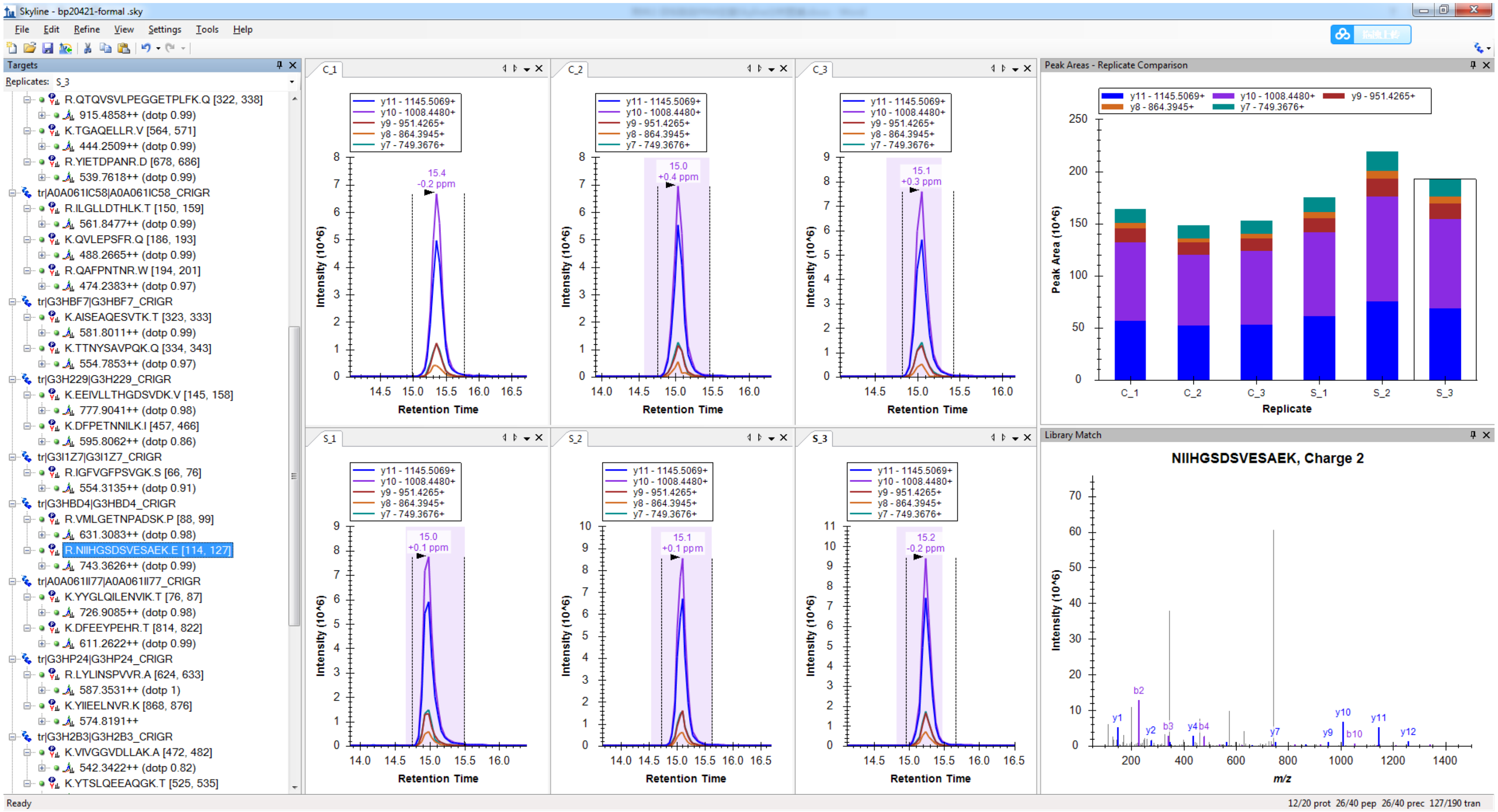

YYGLQILENVIK

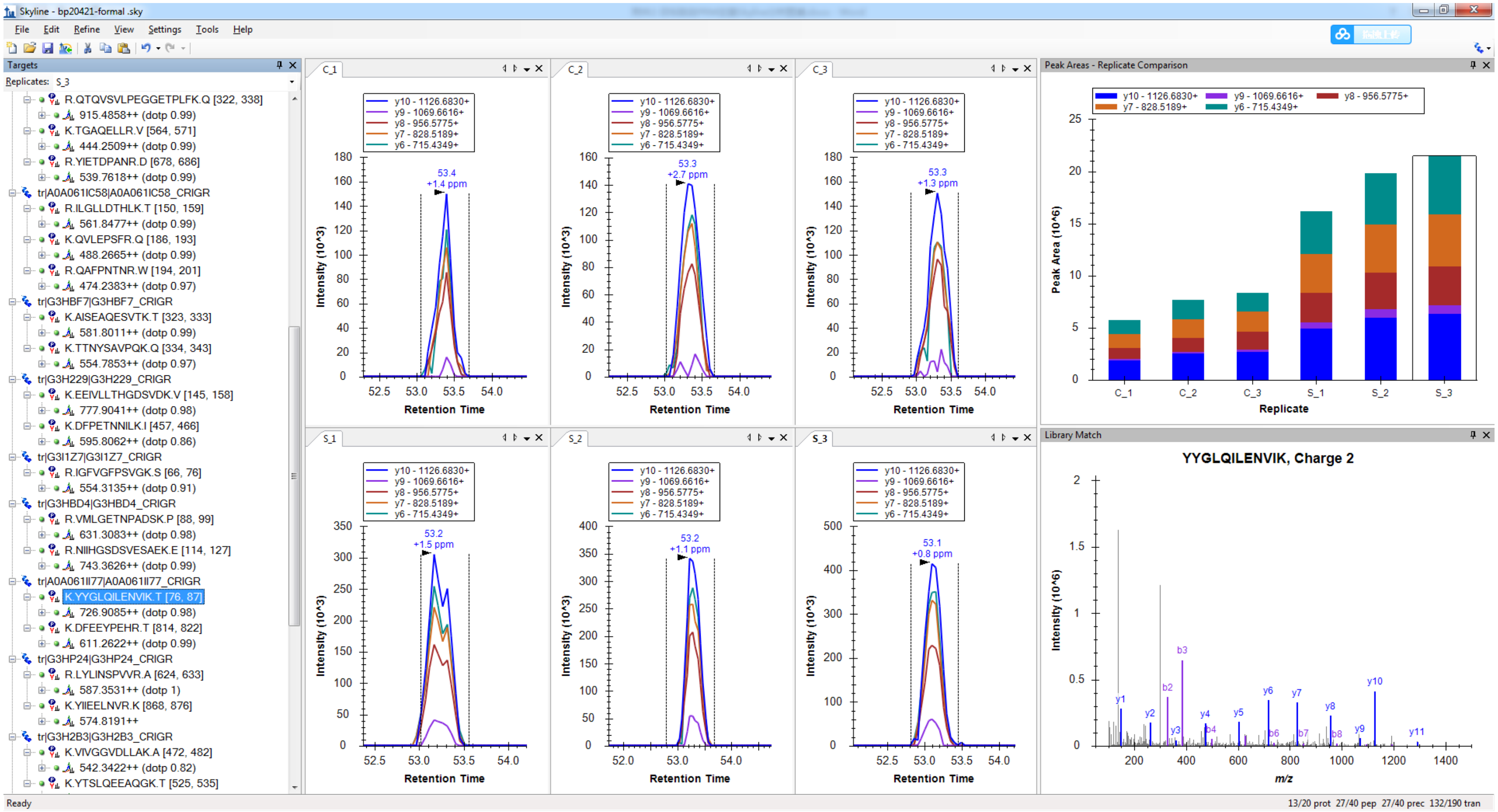

DFEEYPEHR

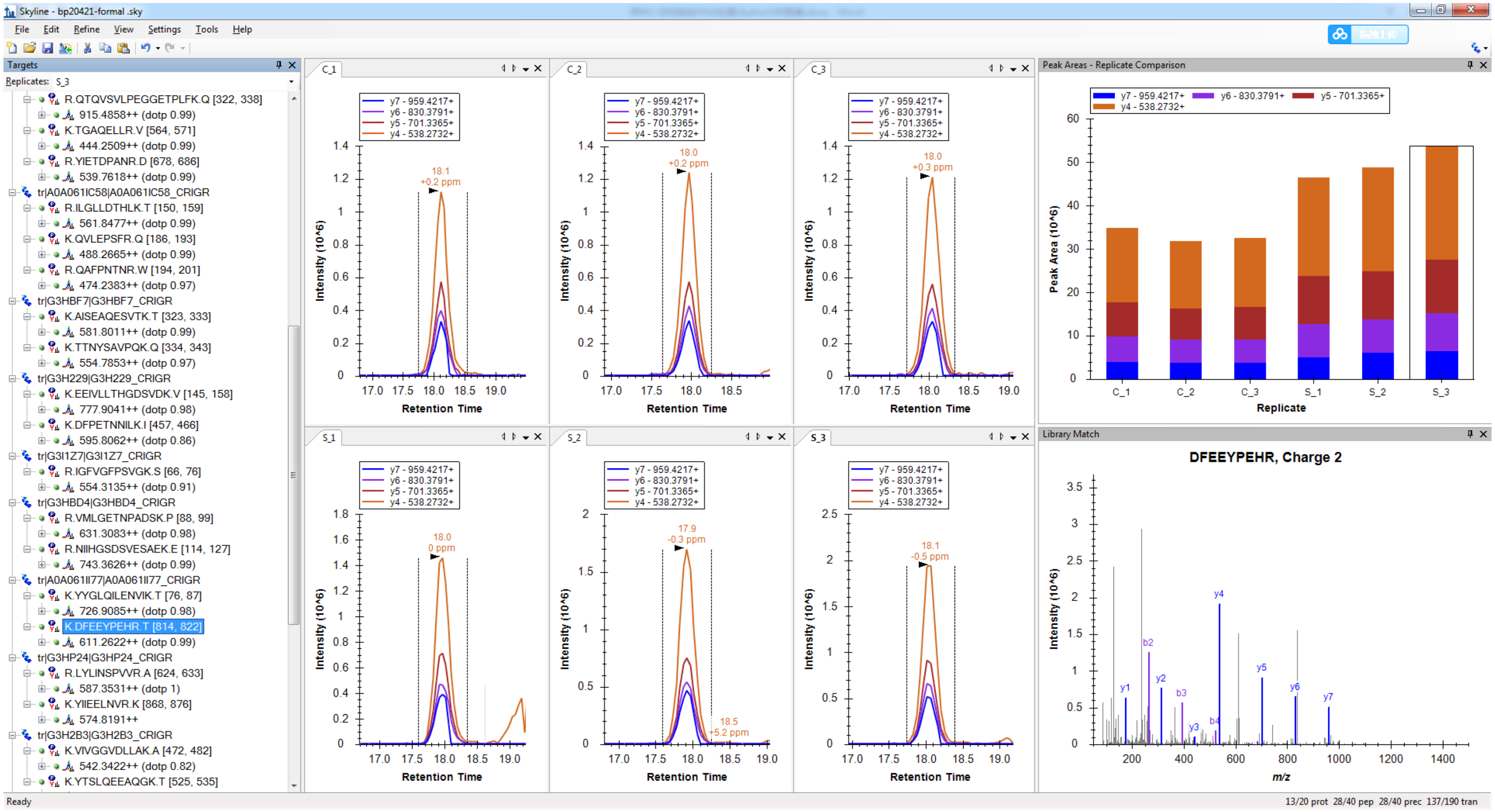

Skyline - bp20421-formal .sky
File Edit Refine View Settings Tools Help
Targets
Replicates: S_3
R.QTQVSVLPEGGETPLFK.Q [322, 338]
915.4858++ (dotp 0.99)
K.TGAQELLR.V [564, 571]
444.2509++ (dotp 0.99)
R.YIETDPANR.D [678, 686]
539.7618++ (dotp 0.99)
tr|A0A061IC58|A0A061IC58_CRIGR
R.ILGLLDTHLK.T [150, 159]
561.8477++ (dotp 0.99)
K.QVLEPSFR.Q [186, 193]
488.2665++ (dotp 0.99)
R.QAFPNTNR.W [194, 201]
474.2383++ (dotp 0.97)
tr|G3HBF7|G3HBF7_CRIGR
K.AISEAQESVTK.T [323, 333]
581.8011++ (dotp 0.99)
K.TTNYSAVPQK.Q [334, 343]
554.7853++ (dotp 0.97)
tr|G3H229|G3H229_CRIGR
K.EEIVLLTHGDSVDK.V [145, 158]
777.9041++ (dotp 0.98)
K.DFPETNNILK.I [457, 466]
595.8062++ (dotp 0.86)
tr|G3I1Z7|G3I1Z7_CRIGR
R.IGFVGFPSVGK.S [66, 76]
554.3135++ (dotp 0.91)
tr|G3HBD4|G3HBD4_CRIGR
R.VMLGETNPADSK.P [88, 99]
631.3083++ (dotp 0.98)
R.NIIHGSDSVESAEK.E [114, 127]
743.3626++ (dotp 0.99)
tr|A0A061II77|A0A061II77_CRIGR
K.YYGLQILENVIK.T [76, 87]
726.9085++ (dotp 0.98)
K.DFEEYPEHR.T [814, 822]
611.2622++ (dotp 0.99)
tr|G3HP24|G3HP24_CRIGR
R.LYLINSPVVR.A [624, 633]
587.3531++ (dotp 1)
K.YIIEELNVR.K [868, 876]
574.8191++
tr|G3H2B3|G3H2B3_CRIGR
K.VIVGGVDLLAK.A [472, 482]
542.3422++ (dotp 0.82)
K.YTSLQEEAQGK.T [525, 535]
C_1
C_2
C_3
S_1
S_2
S_3
y7 - 959.4217+
y6 - 830.3791+
y5 - 701.3365+
y4 - 538.2732+
18.1
+0.2 ppm
18.0
+0.2 ppm
18.0
+0.3 ppm
18.0
0 ppm
17.9
-0.3 ppm
18.5
+5.2 ppm
18.1
-0.5 ppm
Intensity (10^6)
Retention Time
Peak Areas - Replicate Comparison
Peak Area (10^6)
Replicate
Library Match
DFEEYPEHR, Charge 2
Intensity (10^6)
m/z
Ready
13/20 prot 28/40 pep 28/40 prec 137/190 tran

LYLINSPVVR

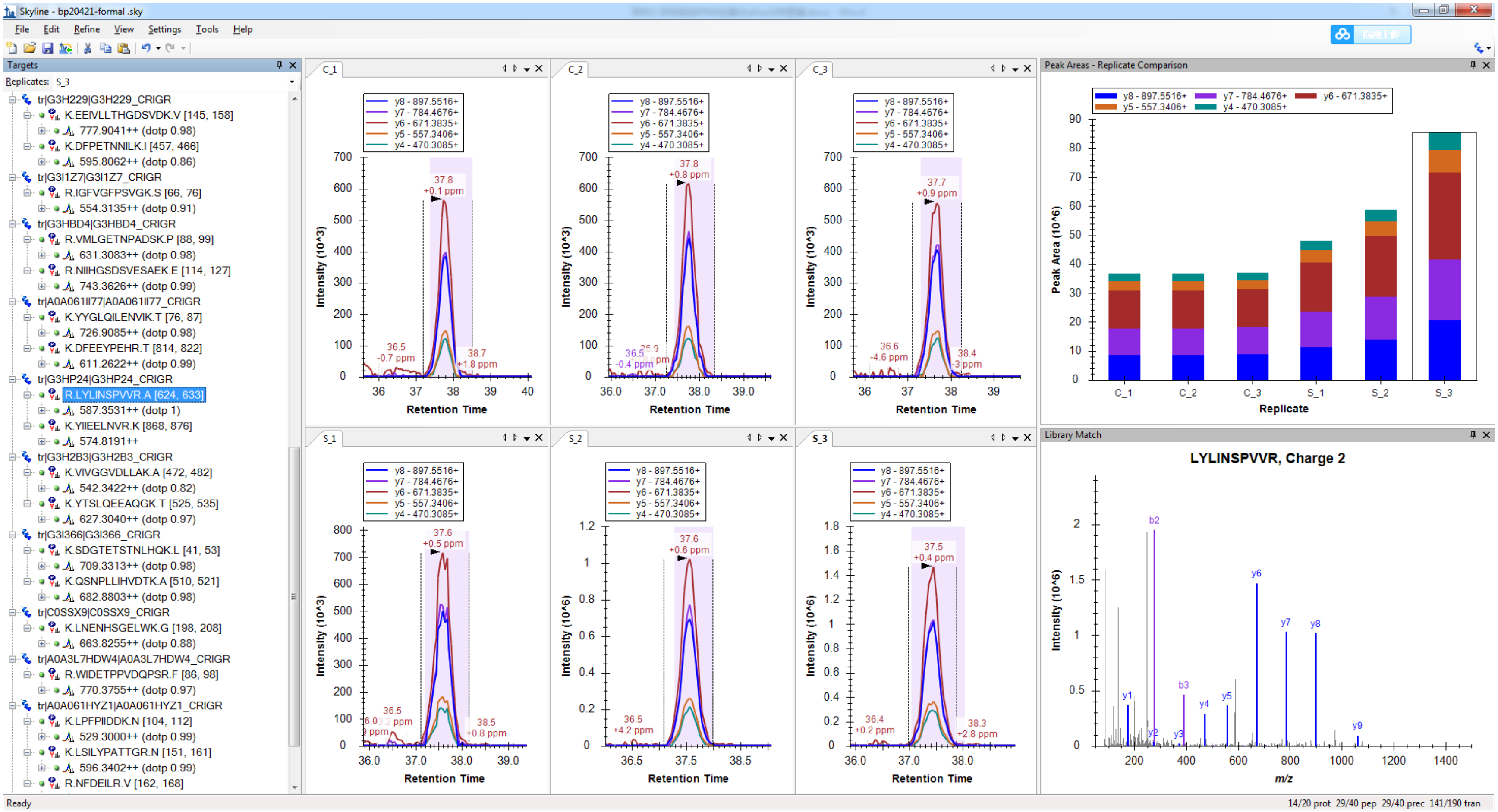

Skyline - bp20421-formal .sky
File Edit Refine View Settings Tools Help
Targets
Replicates: S_3
tr|G3H229|G3H229_CRIGR
K.EEIVLLTHGDSVDK.V [145, 158]
777.9041++ (dotp 0.98)
K.DFPETNNILK.I [457, 466]
595.8062++ (dotp 0.86)
tr|G3I1Z7|G3I1Z7_CRIGR
R.IGFVGFPSVGK.S [66, 76]
554.3135++ (dotp 0.91)
tr|G3HBD4|G3HBD4_CRIGR
R.VMLGETNPADSK.P [88, 99]
631.3083++ (dotp 0.98)
R.NIIHGSDSVESAEK.E [114, 127]
743.3626++ (dotp 0.99)
tr|A0A061II77|A0A061II77_CRIGR
K.YYGLQILENVIK.T [76, 87]
726.9085++ (dotp 0.98)
K.DFEEYPEHR.T [814, 822]
611.2622++ (dotp 0.99)
tr|G3HP24|G3HP24_CRIGR
R.LYLINSPVVR.A [624, 633]
587.3531++ (dotp 1)
K.YIIEELNVR.K [868, 876]
574.8191++
tr|G3H2B3|G3H2B3_CRIGR
K.VIVGGVDLLAK.A [472, 482]
542.3422++ (dotp 0.82)
K.YTSLQEEAQGK.T [525, 535]
627.3040++ (dotp 0.97)
tr|G3I366|G3I366_CRIGR
K.SDGTETSTNLHQK.L [41, 53]
709.3313++ (dotp 0.98)
K.QSNPLLIHVDTK.A [510, 521]
682.8803++ (dotp 0.98)
tr|C0SSX9|C0SSX9_CRIGR
K.LNENHSGELWK.G [198, 208]
663.8255++ (dotp 0.88)
tr|A0A3L7HDW4|A0A3L7HDW4_CRIGR
R.WIDETPPVDQPSR.F [86, 98]
770.3755++ (dotp 0.97)
tr|A0A061HYZ1|A0A061HYZ1_CRIGR
K.LPFPIIDDK.N [104, 112]
529.3000++ (dotp 0.99)
K.LSILYPATTGR.N [151, 161]
596.3402++ (dotp 0.99)
R.NFDEILR.V [162, 168]
C_1
C_2
C_3
S_1
S_2
S_3
y8 - 897.5516+
y7 - 784.4676+
y6 - 671.3835+
y5 - 557.3406+
y4 - 470.3085+
Intensity (10^3)
Intensity (10^6)
Retention Time
37.8 +0.1 ppm
37.8 +0.8 ppm
37.7 +0.9 ppm
37.6 +0.5 ppm
37.6 +0.6 ppm
37.5 +0.4 ppm
Peak Areas - Replicate Comparison
Peak Area (10^6)
Replicate
Library Match
LYLINSPVVR, Charge 2
Intensity (10^6)
m/z
b2
b3
y1
y4
y5
y6
y7
y8
y9
Ready
14/20 prot 29/40 pep 29/40 prec 141/190 tran

YIIEELNVR

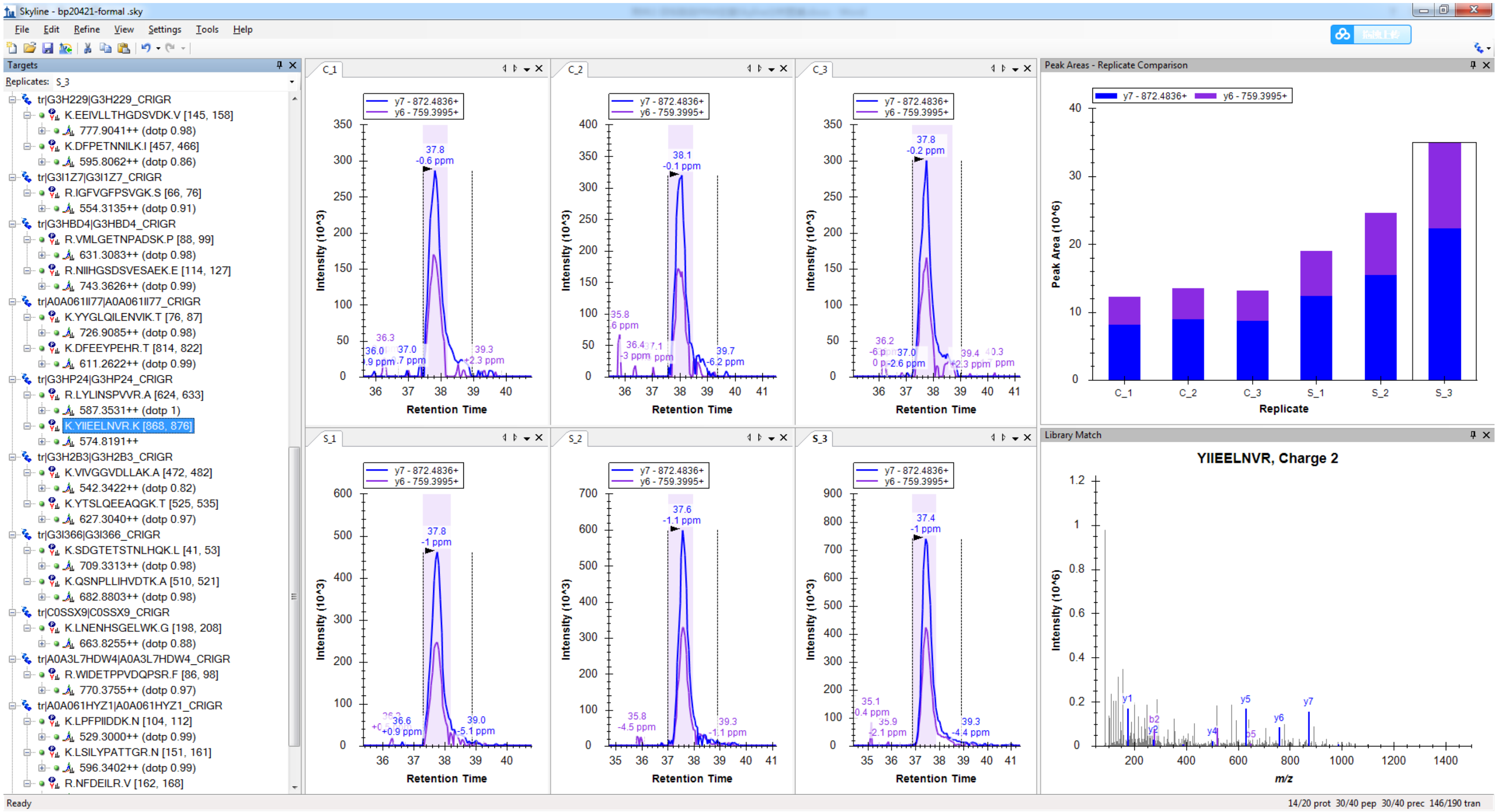

Skyline - bp20421-formal .sky
File Edit Refine View Settings Tools Help
Targets
Replicates: S_3
tr|G3H229|G3H229_CRIGR
K.EEIVLLTHGDSVDK.V [145, 158]
777.9041++ (dotp 0.98)
K.DFPETNNILK.I [457, 466]
595.8062++ (dotp 0.86)
tr|G3I1Z7|G3I1Z7_CRIGR
R.IGFVGFPSVGK.S [66, 76]
554.3135++ (dotp 0.91)
tr|G3HBD4|G3HBD4_CRIGR
R.VMLGETNPADSK.P [88, 99]
631.3083++ (dotp 0.98)
R.NIIHGSDSVESAEK.E [114, 127]
743.3626++ (dotp 0.99)
tr|A0A061II77|A0A061II77_CRIGR
K.YYGLQILENVIK.T [76, 87]
726.9085++ (dotp 0.98)
K.DFEEYPEHR.T [814, 822]
611.2622++ (dotp 0.99)
tr|G3HP24|G3HP24_CRIGR
R.LYLINSPVVR.A [624, 633]
587.3531++ (dotp 1)
K.YIIEELNVR.K [868, 876]
574.8191++
tr|G3H2B3|G3H2B3_CRIGR
K.VIVGGVDLLAK.A [472, 482]
542.3422++ (dotp 0.82)
K.YTSLQEEAQGK.T [525, 535]
627.3040++ (dotp 0.97)
tr|G3I366|G3I366_CRIGR
K.SDGTETSTNLHQK.L [41, 53]
709.3313++ (dotp 0.98)
K.QSNPLLIHVDTK.A [510, 521]
682.8803++ (dotp 0.98)
tr|C0SSX9|C0SSX9_CRIGR
K.LNENHSGELWK.G [198, 208]
663.8255++ (dotp 0.88)
tr|A0A3L7HDW4|A0A3L7HDW4_CRIGR
R.WIDETPPVDQPSR.F [86, 98]
770.3755++ (dotp 0.97)
tr|A0A061HYZ1|A0A061HYZ1_CRIGR
K.LPFPIIDDK.N [104, 112]
529.3000++ (dotp 0.99)
K.LSILYPATTGR.N [151, 161]
596.3402++ (dotp 0.99)
R.NFDEILR.V [162, 168]
C_1
C_2
C_3
S_1
S_2
S_3
y7 - 872.4836+
y6 - 759.3995+
Intensity (10^3)
Retention Time
Peak Areas - Replicate Comparison
Peak Area (10^6)
Replicate
Library Match
YIIEELNVR, Charge 2
Intensity (10^6)
m/z
Ready
14/20 prot 30/40 pep 30/40 prec 146/190 tran

VIVGGVDLLAK

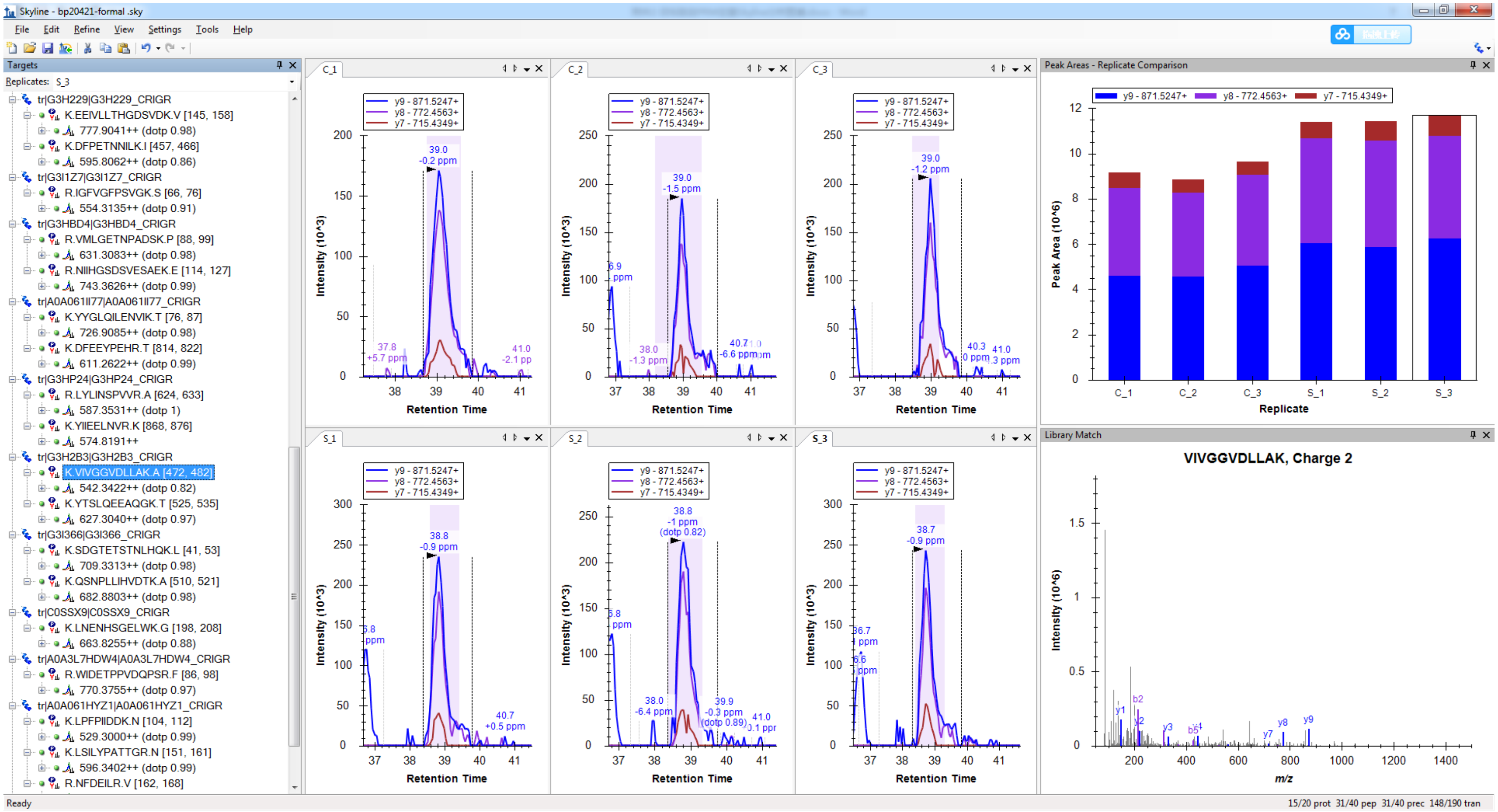

YTSLQEEAQGK

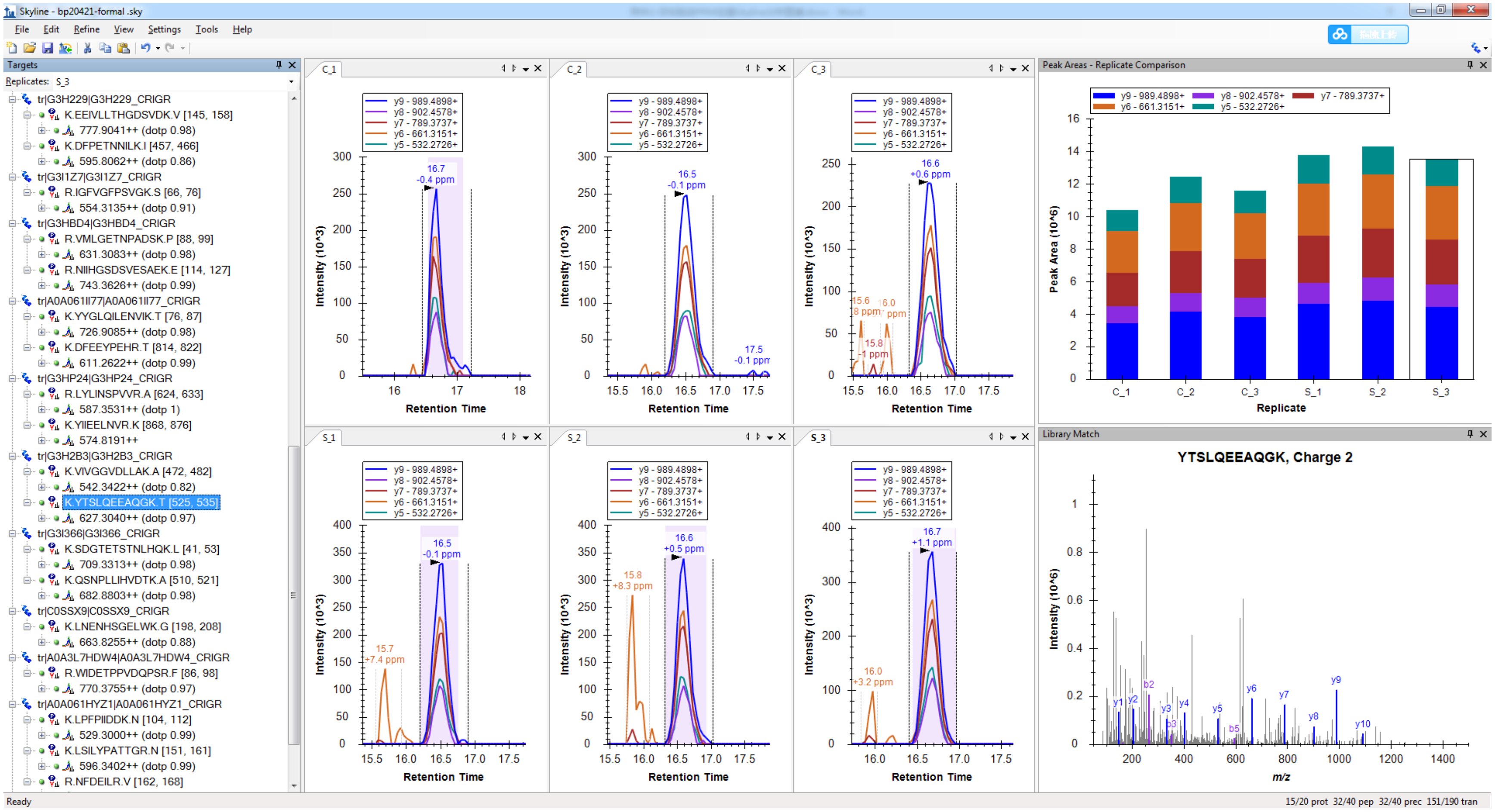

SDGTETSTNLHQK

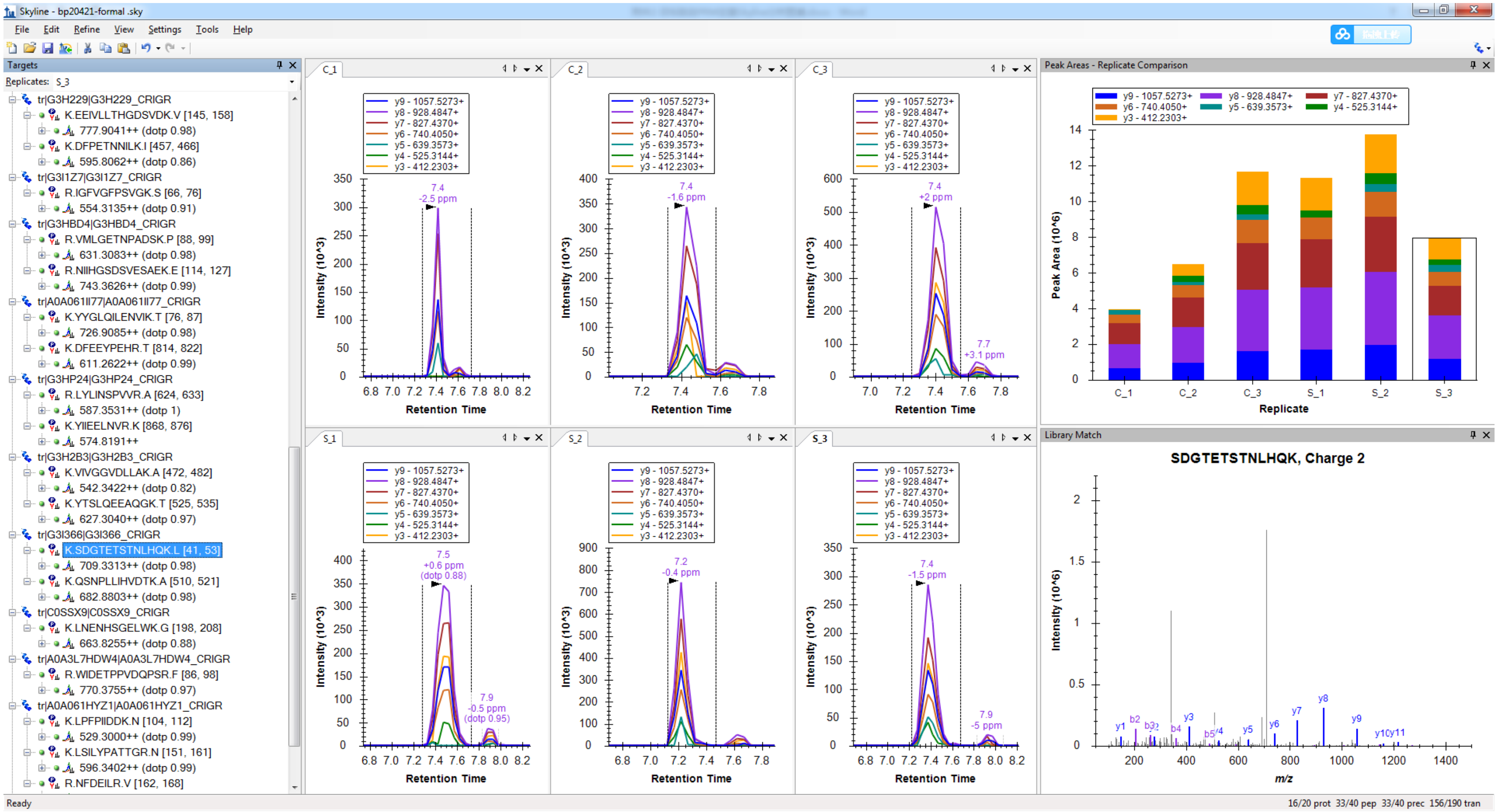

QSNPLLIHVDTK

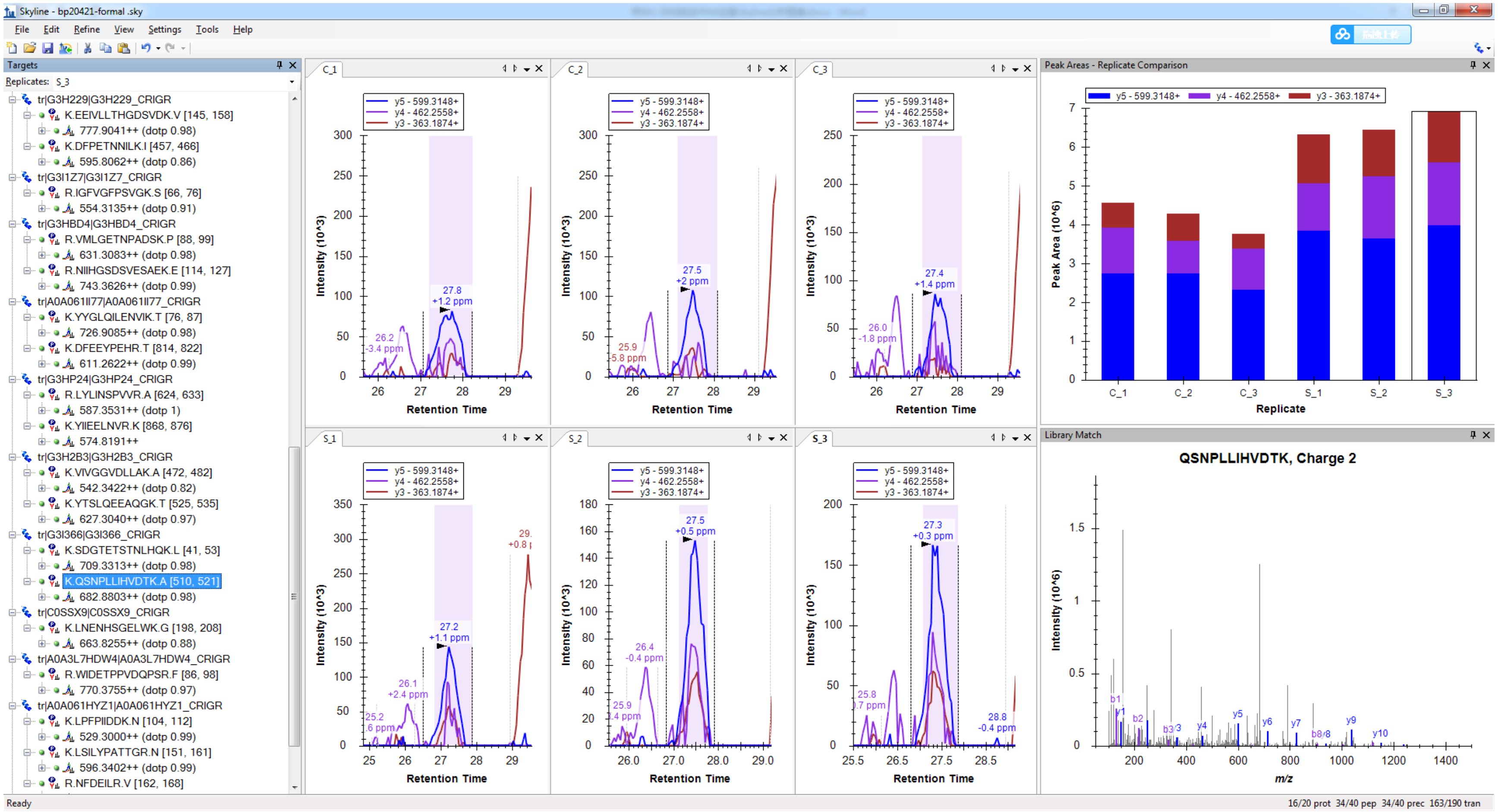

LNENHSGELWK

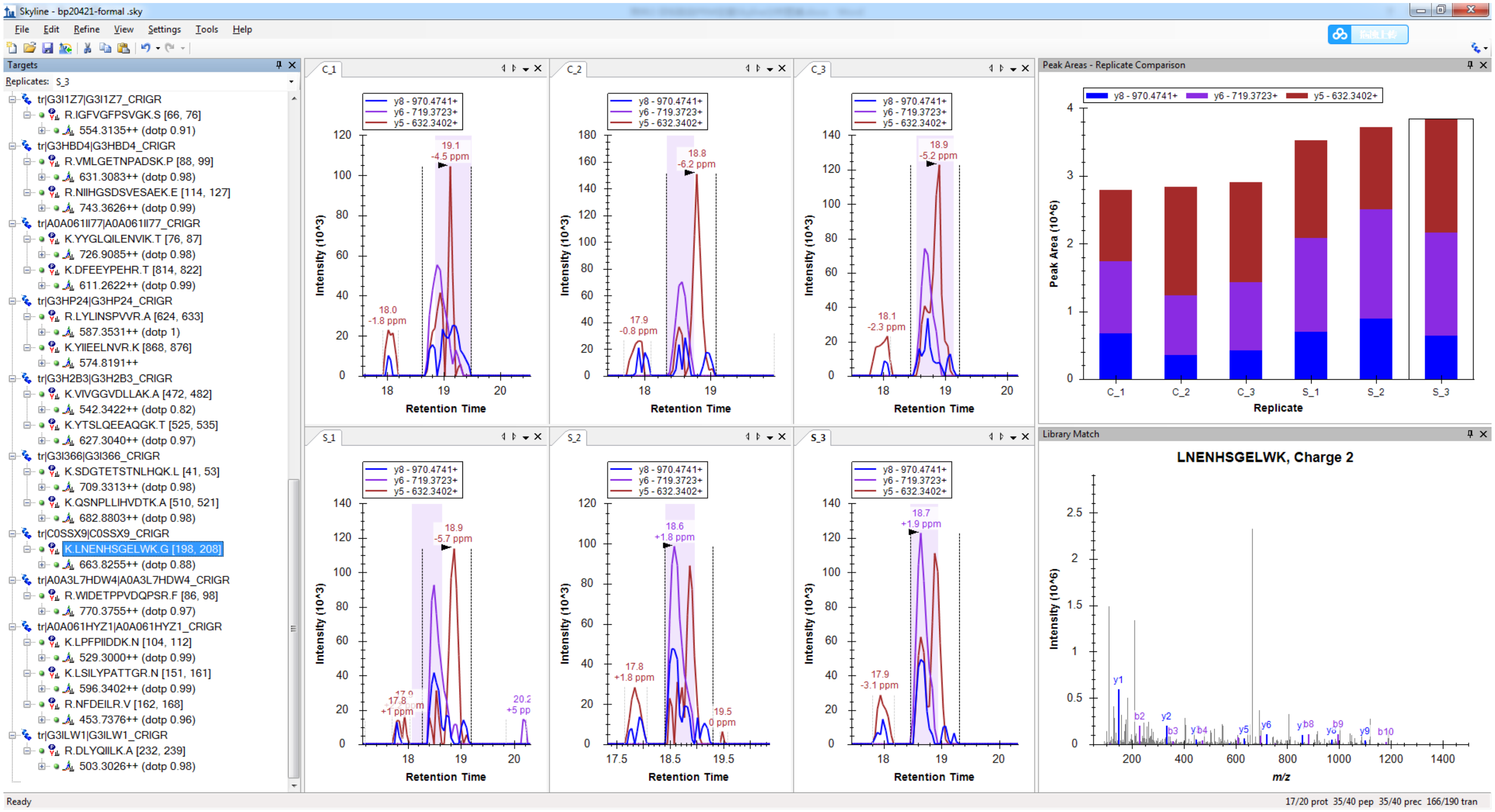

WIDETPPVDQPSR

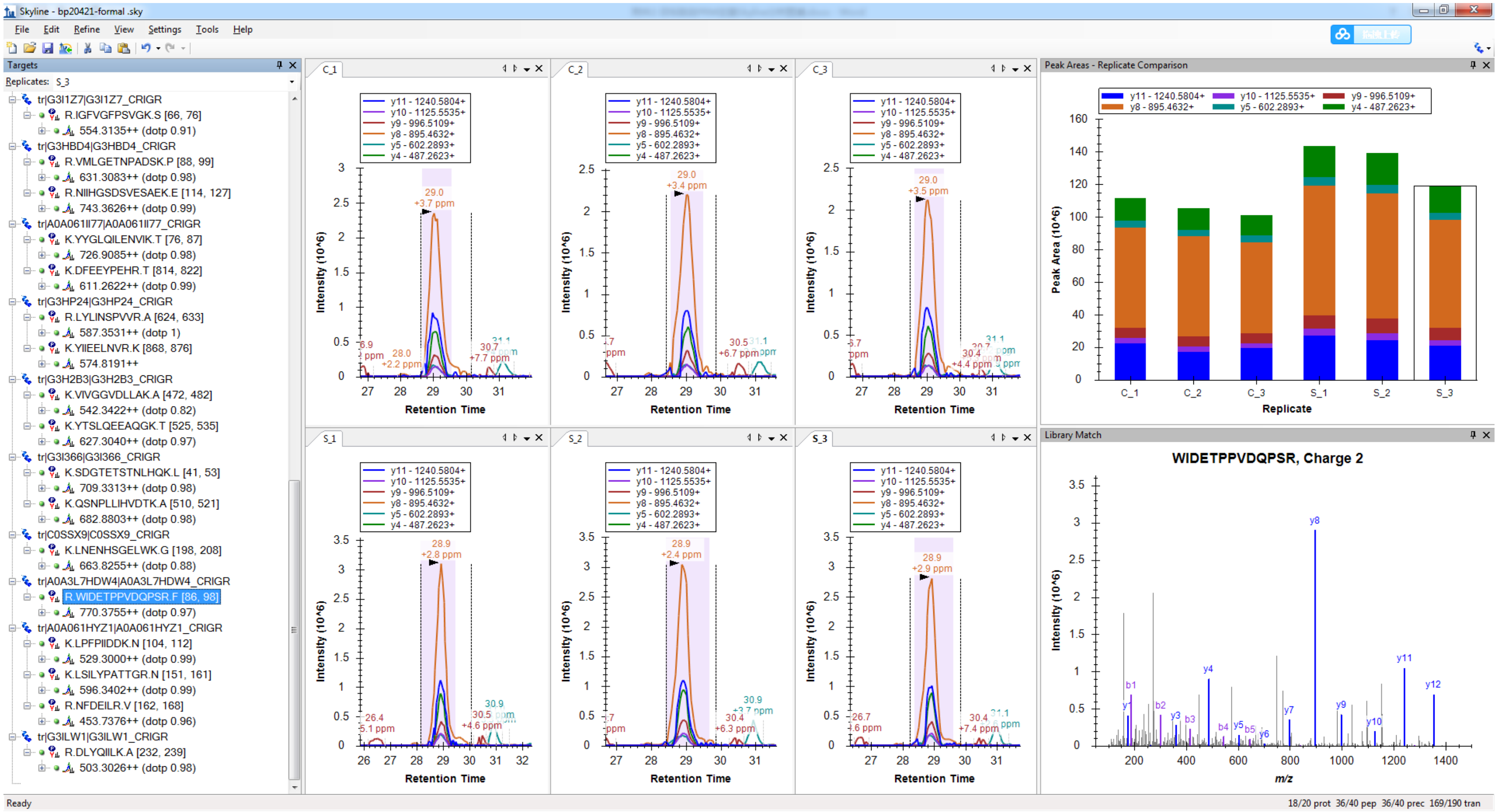

LPFPIIDDK

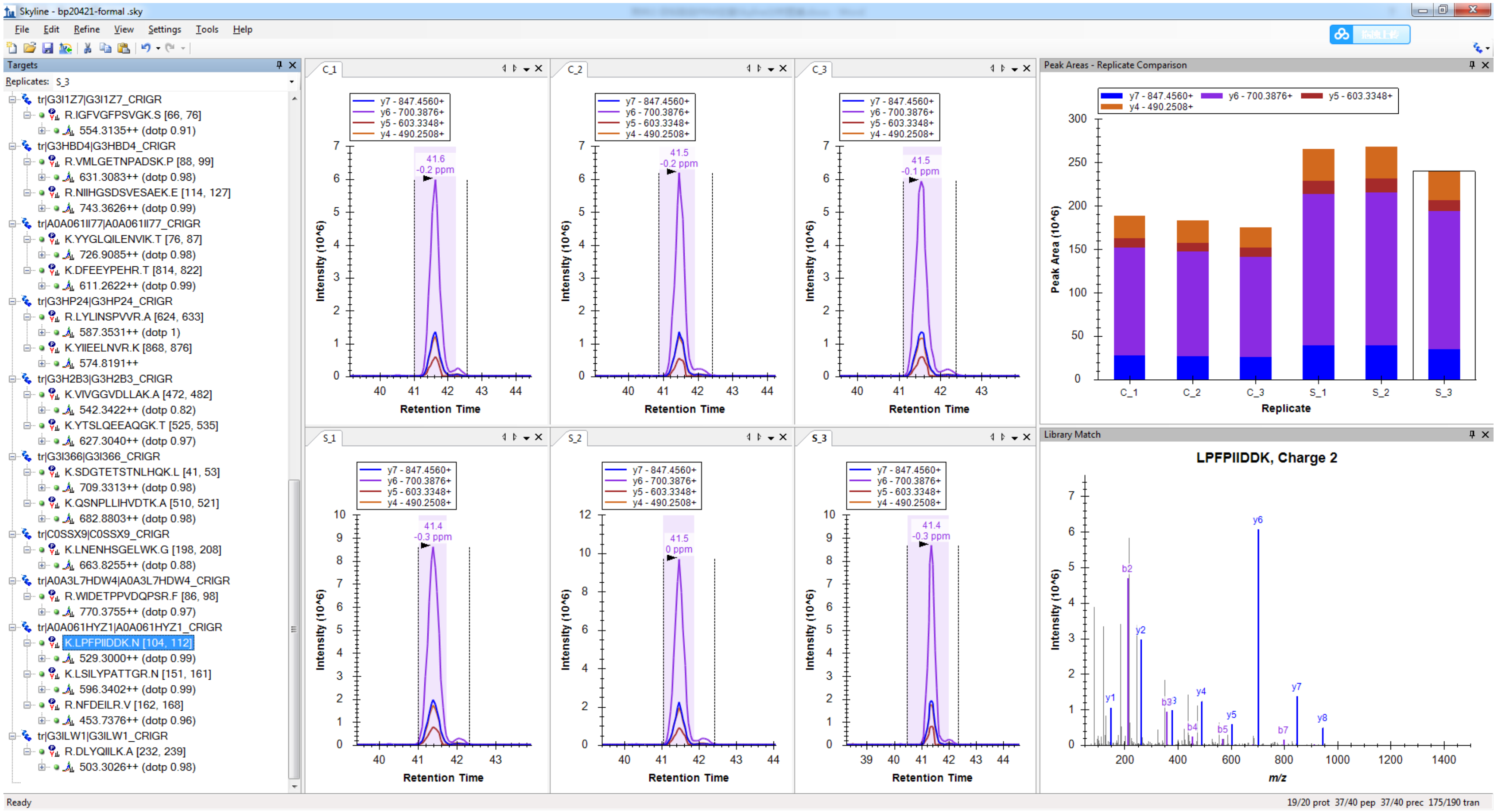

Skyline - bp20421-formal .sky
File Edit Refine View Settings Tools Help
Targets
Replicates: S_3
tr|G3I1Z7|G3I1Z7_CRIGR
R.IGFVGFPSVGK.S [66, 76]
554.3135++ (dotp 0.91)
tr|G3HBD4|G3HBD4_CRIGR
R.VMLGETNPADSK.P [88, 99]
631.3083++ (dotp 0.98)
R.NIIHGSDSVESAEK.E [114, 127]
743.3626++ (dotp 0.99)
tr|A0A061II77|A0A061II77_CRIGR
K.YYGLQILENVIK.T [76, 87]
726.9085++ (dotp 0.98)
K.DFEEYPEHR.T [814, 822]
611.2622++ (dotp 0.99)
tr|G3HP24|G3HP24_CRIGR
R.LYLINSPVVR.A [624, 633]
587.3531++ (dotp 1)
K.YIIEELNVR.K [868, 876]
574.8191++
tr|G3H2B3|G3H2B3_CRIGR
K.VIVGGVDLLAK.A [472, 482]
542.3422++ (dotp 0.82)
K.YTSLQEEAQGK.T [525, 535]
627.3040++ (dotp 0.97)
tr|G3I366|G3I366_CRIGR
K.SDGTETSTNLHQK.L [41, 53]
709.3313++ (dotp 0.98)
K.QSNPLLIHVDTK.A [510, 521]
682.8803++ (dotp 0.98)
tr|C0SSX9|C0SSX9_CRIGR
K.LNENHSGELWK.G [198, 208]
663.8255++ (dotp 0.88)
tr|A0A3L7HDW4|A0A3L7HDW4_CRIGR
R.WIDETPPVDQPSR.F [86, 98]
770.3755++ (dotp 0.97)
tr|A0A061HYZ1|A0A061HYZ1_CRIGR
K.LPFPIIDDK.N [104, 112]
529.3000++ (dotp 0.99)
K.LSILYPATTGR.N [151, 161]
596.3402++ (dotp 0.99)
R.NFDEILR.V [162, 168]
453.7376++ (dotp 0.96)
tr|G3ILW1|G3ILW1_CRIGR
R.DLYQIILK.A [232, 239]
503.3026++ (dotp 0.98)
C_1 C_2 C_3 S_1 S_2 S_3
y7 - 847.4560+
y6 - 700.3876+
y5 - 603.3348+
y4 - 490.2508+
Intensity (10^6)
Retention Time
41.6 -0.2 ppm
41.5 -0.2 ppm
41.5 -0.1 ppm
41.4 -0.3 ppm
41.5 0 ppm
41.4 -0.3 ppm
Peak Areas - Replicate Comparison
Peak Area (10^6)
Replicate
Library Match
LPFPIIDDK, Charge 2
Intensity (10^6)
m/z
y1 b2 y2 b3 b4 y4 b5 y5 y6 b7 y7 y8
Ready
19/20 prot 37/40 pep 37/40 prec 175/190 tran

LSILYPATTGR

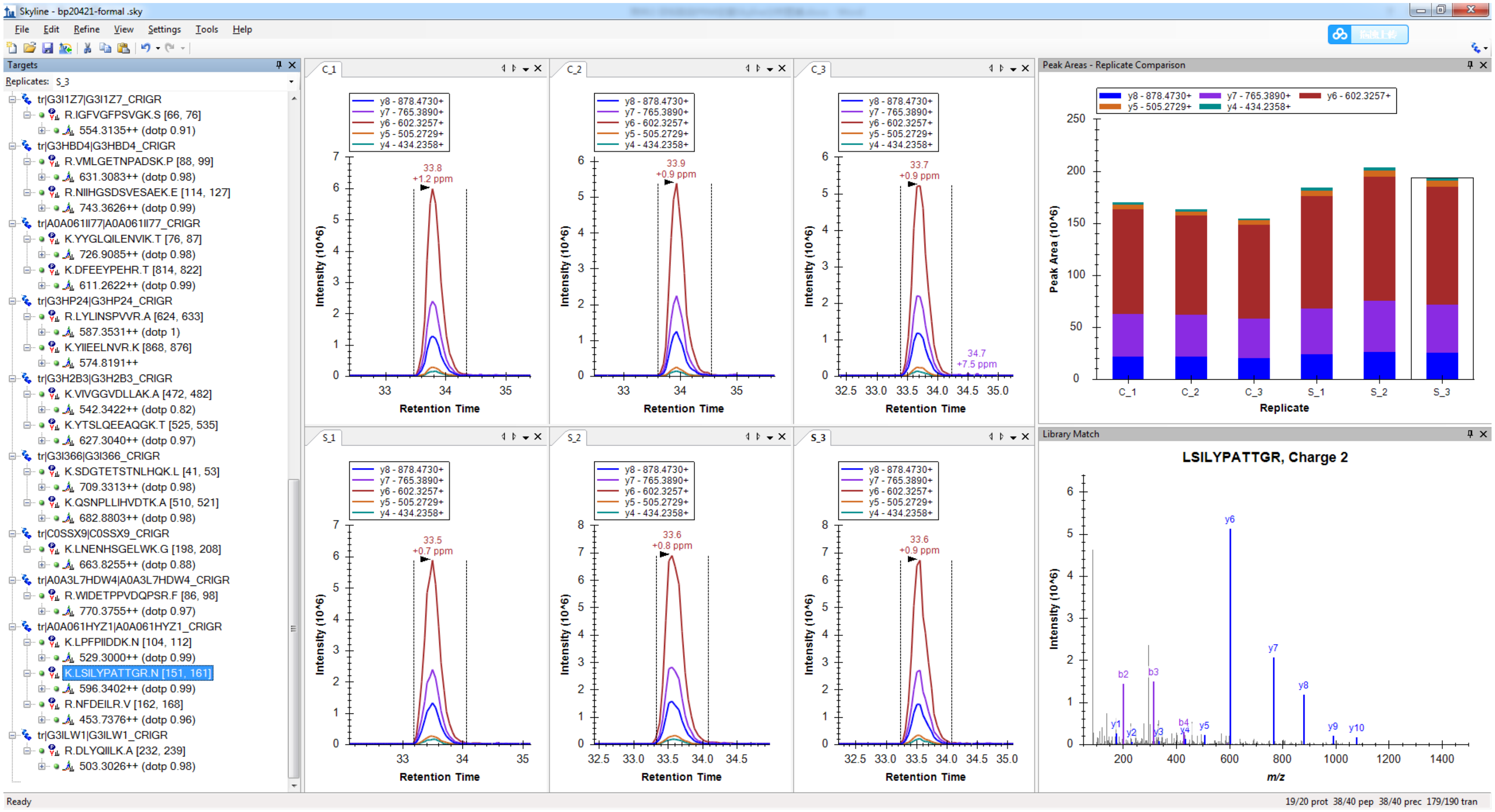

NFDEILR

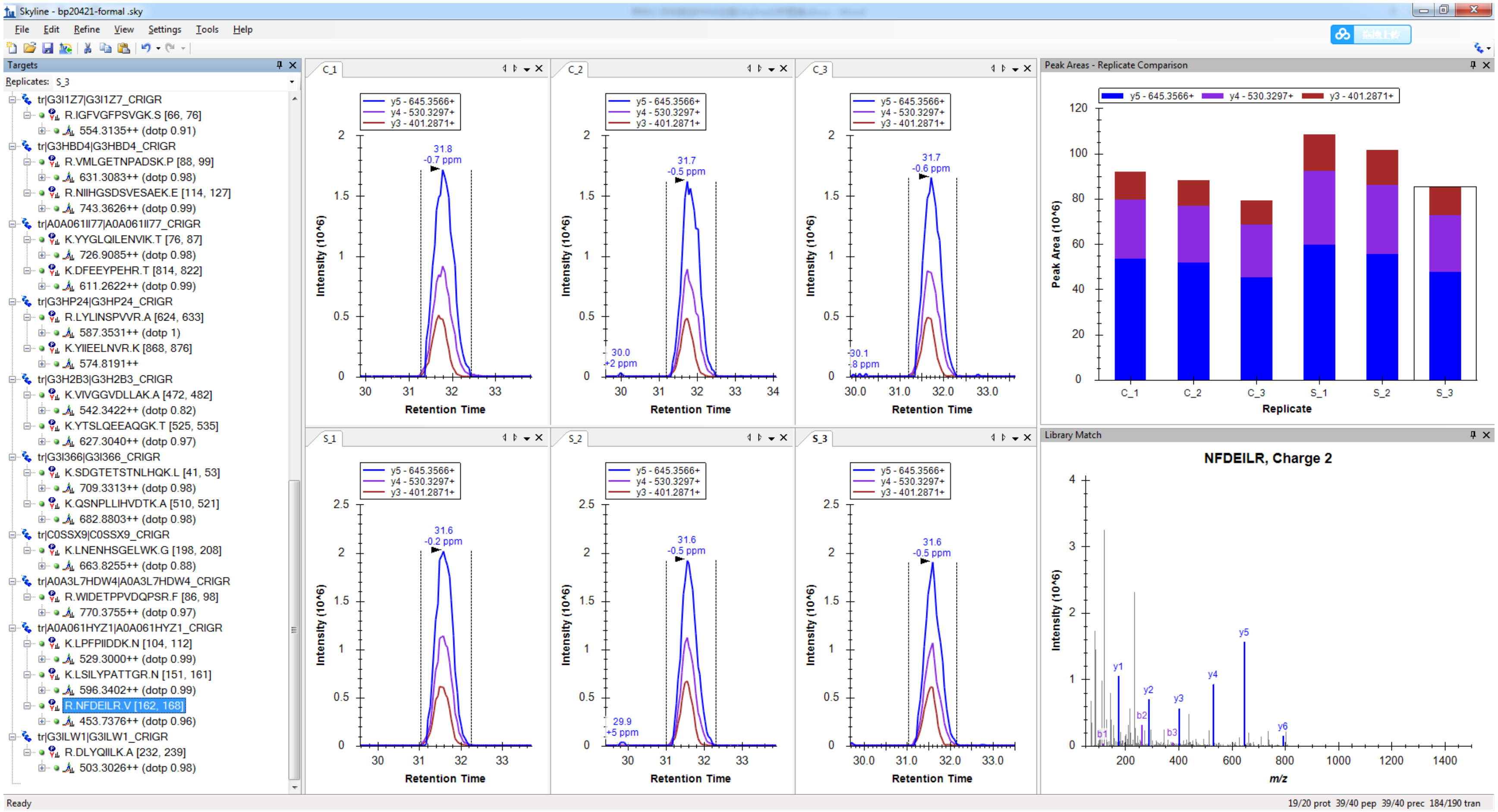
Skyline - bp20421-formal .sky
File
Edit
Refine
View
Settings
Tools
Help
Targets
Replicates: S_3
tr|G3I1Z7|G3I1Z7_CRIGR
R.IGFVGFPSVGK.S [66, 76]
554.3135++ (dotp 0.91)
tr|G3HBD4|G3HBD4_CRIGR
R.VMLGETNPADSK.P [88, 99]
631.3083++ (dotp 0.98)
R.NIIHGSDSVESAEK.E [114, 127]
743.3626++ (dotp 0.99)
tr|A0A061I177|A0A061I177_CRIGR
K.YYGLQILENVIK.T [76, 87]
726.9085++ (dotp 0.98)
K.DFEEYPEHR.T [814, 822]
611.2622++ (dotp 0.99)
tr|G3HP24|G3HP24_CRIGR
R.LYLINSPVVR.A [624, 633]
587.3531++ (dotp 1)
K.YIIEELNVR.K [868, 876]
574.8191++
tr|G3H2B3|G3H2B3_CRIGR
K.VIVGGVDLLAK.A [472, 482]
542.3422++ (dotp 0.82)
K.YTSLQEEAQGK.T [525, 535]
627.3040++ (dotp 0.97)
tr|G3I366|G3I366_CRIGR
K.SDGTETSTNLHQK.L [41, 53]
709.3313++ (dotp 0.98)
K.QSNPLLIHVDTK.A [510, 521]
682.8803++ (dotp 0.98)
tr|C0SSX9|C0SSX9_CRIGR
K.LNENHSGELWK.G [198, 208]
663.8255++ (dotp 0.88)
tr|A0A3L7HDW4|A0A3L7HDW4_CRIGR
R.WIDETPPVDQPSR.F [86, 98]
770.3755++ (dotp 0.97)
tr|A0A061HYZ1|A0A061HYZ1_CRIGR
K.LPFPIIDDK.N [104, 112]
529.3000++ (dotp 0.99)
K.LSILYPATTGR.N [151, 161]
596.3402++ (dotp 0.99)
R.NFDEILR.V [162, 168]
453.7376++ (dotp 0.96)
tr|G3ILW1|G3ILW1_CRIGR
R.DLYQIILK.A [232, 239]
503.3026++ (dotp 0.98)
C_1
C_2
C_3
S_1
S_2
S_3
y5 - 645.3566+
y4 - 530.3297+
y3 - 401.2871+
Intensity (10^6)
Retention Time
Peak Areas - Replicate Comparison
Peak Area (10^6)
Replicate
Library Match
NFDEILR, Charge 2
m/z
Ready
19/20 prot 39/40 pep 39/40 prec 184/190 tran

DLYQIILK

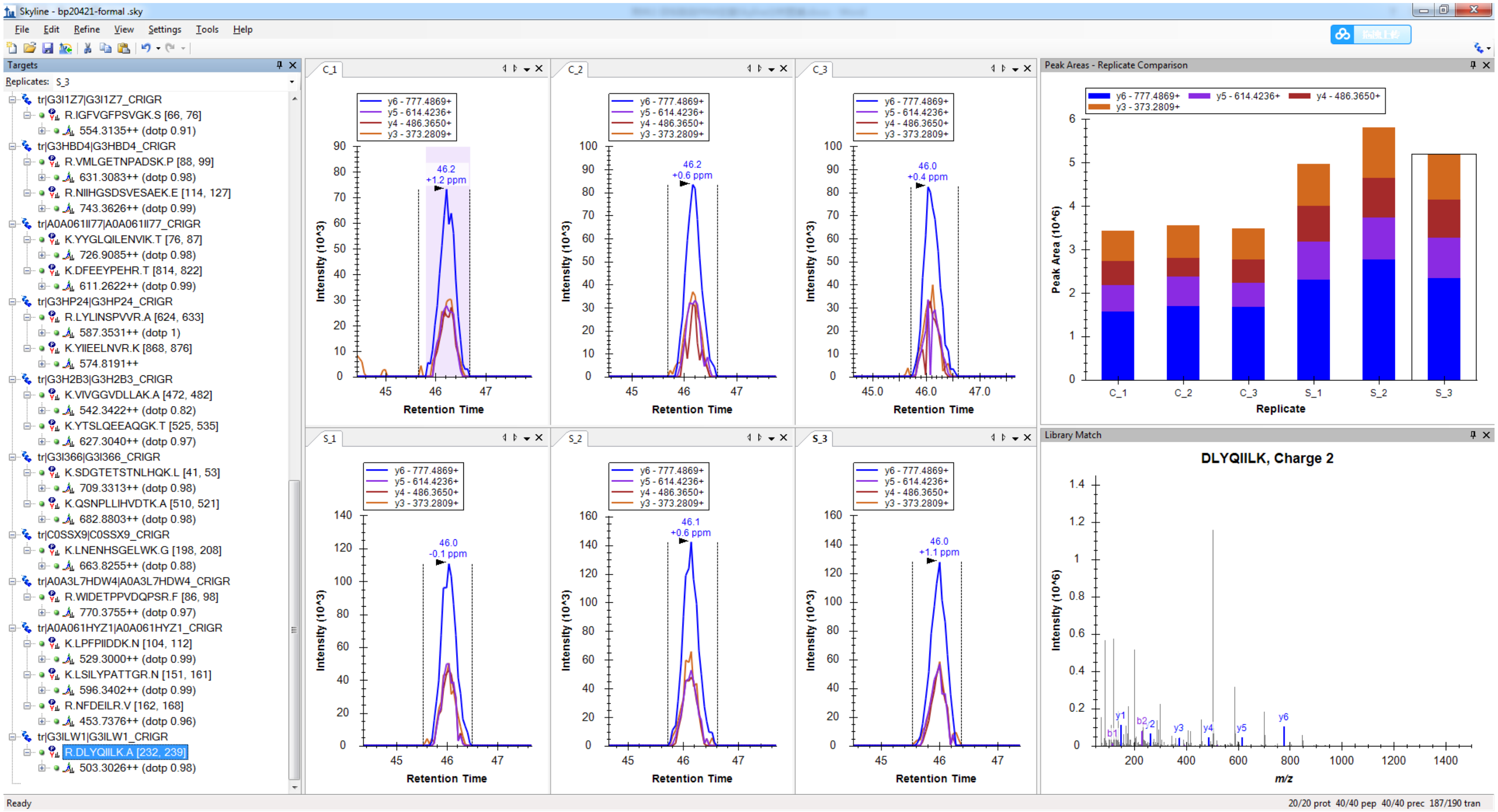

Supplement: Supplementary file 1 [file viruses-13-01036-s001.zip › viruses-1201879-supplementary/Supplementary S11.pdf]
